# Dereplication of New Saponins from *Agave bracteosa*

**DOI:** 10.3390/plants13182570

**Published:** 2024-09-13

**Authors:** Francesca Guzzo, Alexandra G. Durán, Laura L. Rostoll, Francisco A. Macías, Ana M. Simonet

**Affiliations:** 1 Allelopathy Group, Department of Organic Chemistry, Institute of Biomolecules (INBIO), School of Science, University of Cádiz, 11510 Puerto Real, Cádiz, Spain; francesca.guzzo@uca.es (F.G.); alexandra.garcia@uca.es (A.G.D.); laura.lopezros@alum.uca.es (L.L.R.); famacias@uca.es (F.A.M.); 2 Department of Environmental Biological and Pharmaceutical Sciences and Technologies-DiSTABiF, University of Campania “Luigi Vanvitelli”, Via Vivaldi 43, 81100 Caserta, Italy

**Keywords:** saponins, furostane, *Agave bracteosa*, dereplication, HMAI, (25*S*)-cantalasaponin-1, bractofuranosides A–H

## Abstract

The genus *Agave* comprises over 400 species that are known for their diverse applications, which include being sources of fiber, food, and beverages. There has recently been increased interest in exploring the metabolic content of this genus, and in this respect, saponins are the main compounds of interest. Saponins for *Agave bracteosa* have not been described to date, and the current work addresses the dereplication of a saponin-rich fraction to identify the structures of six compounds. The dereplication methods involve the use of UPLC-MS^E^ analysis, NMR spectroscopy and published data for *Agave* saponins. A green extraction and isolation provided ten pure saponins. Remarkably, nine of these saponins have not been reported previously, namely (25*S*)-cantalasaponin-1 and bractofuranosides A–H. These compounds were tested for cytotoxic activity. Bractofuranosides B (**5**) and G (**10**) displayed 57% and 53% cell viability on HeLa cells at 100 µM, respectively.

## 1. Introduction

The Agavaceae family is widely distributed in tropical and subtropical regions and comprises more than 400 species. These plants have been used since ancient times as a source of fiber, food, and drinks, and they are increasingly being investigated for their potential as nutraceuticals, prebiotics, natural sweeteners, and biofuels [1,2]. Specialized metabolites of different chemical classes have been reported in *Agave* species, and these are mainly steroidal saponins [3]. Some biological activities of saponins, such as antimicrobial activity, antidiabetic activity, anticancer activity, and others, have been reported. Quinoa saponins extracted from the husks of *Chenopodium quinoa* are reported to possess antibacterial properties against *Staphylococcus aureus*, *S. epidermidis*, and *Bacillus cereus* as they cause severe damage through bacterial cell wall degradation followed by disruption of the cytoplasmic membrane and membrane proteins [4]. Saponins from *Panax notoginseng* have been reported to have antidiabetic properties as well as an ability to reduce the increased blood plasma glucose [5,6]. Furthermore, numerous steroidal saponins have been explored for their cytotoxic activity, and these have provided interesting research targets for many scientists [7]. Seven timosaponins isolated from the rhizome of *Anemarrhena asphodeloides* showed promising anti-proliferative activity against BEL-7402, HT-29, HeLa, and MDA-MB-468 cell lines in vitro [8]. Spirostanol saponins from flowers of *Allium porrum* were also evaluated in vitro and had a strong cytotoxic effect against mouse peritoneal cells C57BL6 [9]. Steroidal saponins isolated from a methanol extract of whole plants of *Aspidistra letreae* exhibited significant cytotoxicity against five monolayer cancer cell lines, namely LU-1, HeLa, MDA-MB-231, HepG2, and MKN-7 [10].

In most plant species, saponins are often found as a mixture of compounds with very similar polarities. Consequently, the separation and isolation of these mixtures involves a complex and time-consuming process that requires several purification stages that combine different chromatographic techniques and solvents. One of the species belonging to the *Agave* genus yet to be explored is *A. bracteosa*. It is worth noting that only one previous phytochemical study has been carried out on the leaves of *A. bracteosa* [11]. A screening of 13 enriched saponin fractions (SF) of *Agave* species was performed, and this led to the identification of the aglycones using the HMAI method (HMBC Method for Aglycone Identification) [12] in conjunction with the fragmentation obtained from UPLC-MS^E^ analysis. In the case of *A. bracteosa*, the chromatogram showed two peaks at retention times of 0.70 min and 1.38 min. The latter peak corresponded to a saponin with paniculogenin as the aglycone, while the former peak (0.70 min) resulted from the coelution of several furostanic saponins. In addition, saponins with aglycones that are not included in the HMAI method were observed. 

Considering that *A. bracteosa* saponins had unusual structures with strong polarity, the aim of the study reported here was to determine the appropriate extraction method for furostanic saponins and to dereplicate the saponin fraction (SF) by UPLC-MS^E^ analysis, 1D- and 2D-NMR experiments and the HMAI method. Furthermore, in view of the saponins dereplicated in the SF, the isolation and structural characterization of pure compounds was carried out, and the cytotoxic activities were evaluated.

## 2. Results and Discussion

### 2.1. Aqueous Extraction of Saponins from A. bracteosa

The extraction method previously used for *A. bracteosa* [11] was appropriated for spirostanic saponins, although it was observed that this plant also contained a significant proportion of furostanic-type saponins. To identify an extraction method suitable for furostanic saponins, a green and more efficient approach was employed in this work. An ultrasound-assisted extraction was performed on dry leaves, and this involved the use of water as the only extraction solvent [13,14].

The two extraction methods were compared using 0.5 g of dried leaves, and the resulting crude extracts were directly chromatographed on SPE-C18. Different ratios of methanol:water as the mobile phase with the aim of removing the sugar component and the less polar compounds to provide saponin-rich fractions (SF). Comparison of the SF yields in relation to the dry plant showed that the use of the new extraction method (26.5% yield) with water was better than the method in which *n*-butanol:water 1:1 was used (5.5% yield).

### 2.2. UPLC-MS^E^ Analysis of Saponins from A. bracteosa

**Figure 1:**
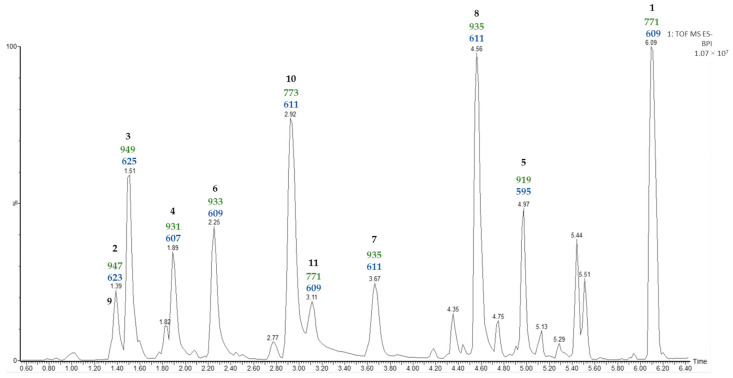
UPLC-MS^E^ chromatogram of the saponin fraction (SF). Values (Da) for [M—H]^−^ (green) and [Aglycone—H + 162]^−^ (blue) fragmentations are displayed for the major saponin peaks.

In the chromatographic method used in the previous study [11], it was found that furostanic-type saponins co-eluted. Consequently, in the study reported here, a new UPLC-MS^E^ analysis was carried out on the SF that considered the high polarity of these saponins (Figure 1). 

The most common ion in negative mode observed in the UPLC-MS^E^ analysis of the SF was [M + HCOO]^−^. Moreover, the second function provides information on the molecular ion [M—H]^–^ and the fragmentations of the saponins. The fragmentations observed for all saponins corresponded to the losses of hexose units, and the last one corresponded to [Aglycone—H + 162]^−^. The sugar unit directly linked to the aglycone should be a hexose (162 Da), and this is consistent with *Agave* saponins [3]. 

In a previous study [11] on the SF of *A. bracteosa* the main saponin (50%) contained paniculogenin as the aglycone and appeared as the last peak in the UPLC-MS^E^ analysis. A detailed examination of the molecular ion, as well as the fragmentation observed from the MS^E^ of the last peak at a retention time of 6.09 min in the water extract analysis, allows the same saponin to be proposed in the case reported here. On the other hand, several of the peaks are likely to correspond to furostanic-type saponins. The value for the last fragment [Aglycone—H + 162]^−^ could indicate the functionalization of the aglycone contained in the SF. For example, *m*/*z* 595 (Rt 4.97 min) indicates a furostanic aglycone without functionalization, but differences of 16 Da suggest oxidation *m*/*z* 611 (Rt 2.92, 3.67, 4.56 min), and differences of 14 Da suggest oxidation and unsaturation *m*/*z* 609 (Rt 2.25, 3.11 min). Finally, two pairs of isomers were detected with identical molecular ion and fragmentation patterns, and subsequent UPLC-MS^E^ analysis did not allow the differentiation of these. 

### 2.3. Dereplication of Aglycones

The dereplication of saponin-rich fractions from *Agave* species has recently been described [15]. The HMAI method (HMBC Method for Aglycone Identification) [12] was proposed for the dereplication of the aglycones, and the second dereplication of the sugar chains was performed separately. The combination of these two parts allowed the identification of the saponins. The tools employed for dereplication are two-dimensional NMR spectra, UPLC-MS^E^ analysis of the enriched fraction, and the spectroscopic data reported in the literature for the genus *Agave.*

**Figure 2:**
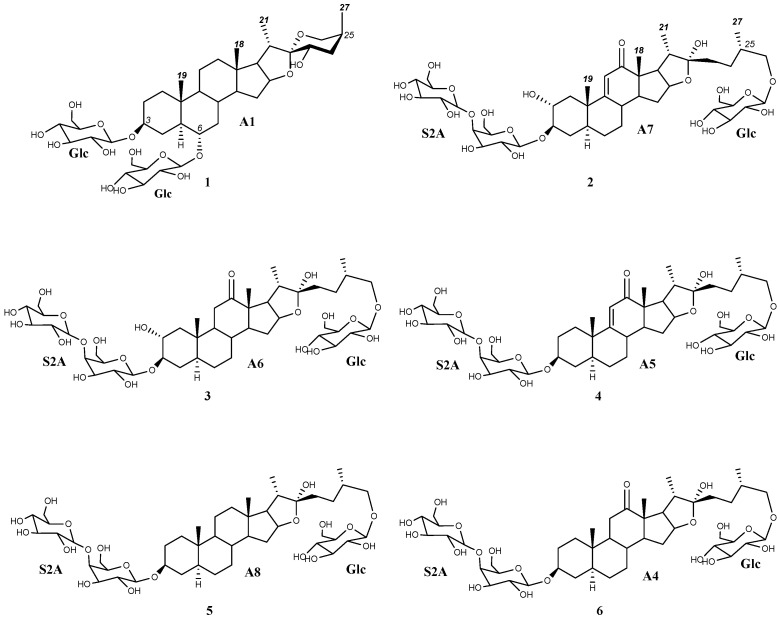
Dereplicated saponins (**1**–**6**) with aglycones (A1 and A4–A8) arising from the application of the HMAI method to *A. bracteosa* SF.

Regarding the aglycone dereplication, the HMAI method is based on the spectroscopic data previously reported for *Agave* saponins by evaluating the influence of the functional groups and structural characteristics of the aglycones on the ^1^H and ^13^C NMR signals. It is proposed that this information is obtained by considering the HMBC correlations of methyl groups based on their intensity and their easy identification in the ^1^H NMR spectrum. The methyl signals are usually shielded between 0.5 and 1.5 ppm, and the signal shape is also characteristic: singlet signals for H-18 and H-19 and doublet signals for H-21 and H-27 (Figure 2). To identify the structural characteristics and functionalization of the aglycones, several decisions included in two flowcharts must be made (Supplementary Materials, Figures S1 and S2), thus allowing each methyl to be assigned and the corresponding information to be obtained. Finally, the proposed structures are checked using the data tables included in the method (Supplementary Materials, Tables S1 and S2) [11]. 

The HMBC correlations of four major doublets and singlets of the SF (Table 1, Supplementary Materials, Figures S1 and S2) were the same as those previously described for paniculogenin (A1) from *A. bracteosa* (Figure 2) [11]. In the cases of the *n*-butanol:water 1:1 and ultrasound water extraction methods, the saponin with paniculogenin as the aglycone was the most abundant. Even though some saponins with this aglycone have been reported in *Solanum hispidum* [16], this is the first time that paniculogenin as aglycone has been described for the genus *Agave*. Paniculogenin is the epimer at C-25 to hongguanggenin, which has already been found in this genus, especially as part of cantalasaponin-1 [15].

Moreover, other correlations have been observed for the remaining saponins in the UPLC-MS^E^ analysis (Figure 1). Specifically, a group of doublet signals found between 1.01 and 0.98 ppm (Table 1) were the only ones to be identified for methyl C-27, as they exhibited two correlations at 28.2 and 75.1 ppm (decision D1 in the doublet flowchart). Discarding the presence of spirostanic-type saponins (D3), it was proposed that all other saponins were furostanic. Furthermore, the presence of a singlet at 3.22 ppm in the ^1^H NMR spectrum (D4) indicates that a significant proportion of the furostanic saponins were methoxylated due to the use of methanol [17] to obtain the SF with a C-18 SPE, which confirmed the presence of this type of saponin. All the signals assigned to methyl C-19 (0.88, 0.70, 0.67 ppm) corresponded to saponins with the H-5α stereochemistry, as usually observed in *Agave* species.

**Figure 3:**
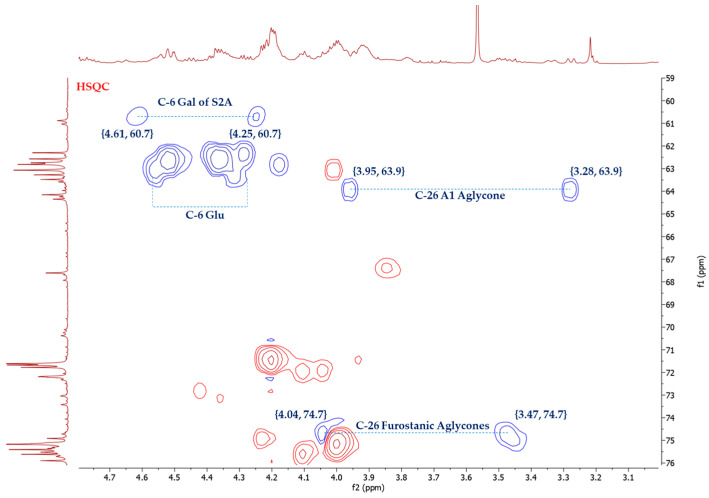
Selected area of HSQC spectrum of *A. bracteosa* SF.

The stereochemistry of C-25 in furostanic aglycones was defined by applying the Agrawal method [18], which is based on the difference between the signals (Δ_ab_ = δa − δb) of glycosylated methylene H_2_-26. In the case of 25*S*, this difference is usually >0.57 ppm, and for 25*R*, it is <0.48. The HSQC spectrum (Figure 3) of the SF provided the chemical shifts of H-26, δ_H_ 4.04, and δ_H_ 3.47. Thus, Δ_ab_(H_2_-26) = 0.57 ppm, and the configuration of this carbon was, therefore, *S*. (25*S*)-5α-furostane-type saponins are not common in the *Agave* [3]. 

Two singlet signals at 1.21 and 0.97 ppm, which correspond to H-18 or H-19, showed correlations that did not match with any structural or functional features included in the HMAI method (Table 1). Therefore, the isolation and structural elucidation of the saponins containing these aglycones (A2 and A3) was necessary. It is interesting to note that both signals showed a correlation at around 80 ppm and this is indicative of an oxygenated position up to three bonds away. Regarding this type of functionalization in *Agave* saponins, only a hydroxyl group at C-12 has been reported. However, this possibility was ruled out since it would have been detected in the HMAI method.

Correlations for furostanic-type saponins with a carbonyl at C-12 and their combination with a double bond between C9 and C11, as well as a hydroxyl group at C-2, have been observed (Table 1. Supplementary Materials, Figure S4). The combination of these functionalizations led to four possible aglycones that could belong to four [Agl + 162—H]^−^ fragments found in the UPLC-MS^E^ spectrum: (25*S*)-3β,22,26-trihydroxy-5α-furostan-12-one (A4, 609 Da), (25*S*)-3β,22,26-trihydroxy-9(11)-dehydro-5α-furostan-12-one (A5, 607 Da), and (25*S*)-2α,3β,22,26-tetrahydroxy-5α-furostan-12-one (A6, 625 Da) and (25*S*)-2α,3β,22,26-tetrahydroxy-9(11)-dehydro-5α-furostan-12-one (A7, 623 Da) (Figure 2).

Another aglycone that was dereplicated was (25*S*)-5α-3β,22,26-trihydroxyfurostane, as this would explain the fragment [Agl + 162—H]^−^ at 595 Da (A8) (Figure 2). Finally, oxygenation was found in three furostanic saponins, corresponding to a fragment with a mass of 611 Da. In the first instance, HMAI information led to the proposed presence of hydroxylated furostanic saponins at position C-2 or C-6. 

It is worth noting that the proposed aglycones arise from the combination of structural features previously described in the HMAI method and that have been dereplicated, even when they had not been used to design the HMAI method [12].

### 2.4. Dereplication of Sugar Chains

Once the dereplication of aglycones had been performed, the sugar chain moieties were explored. The fragmentation observed in the UPLC-MS^E^ analysis revealed that all monosaccharides present in these saponins were hexose units. 

First, application of the HMAI method indicated a d-glucopyranosyloxy bonded at positions C-3 and C-6 for the aglycone paniculogenin (A1). In addition, it has been reported that most furostanic-type saponins contain a d-glucopyranose unit at C-26 and that position C-3 is usually glycosylated. Considering that up to three hexose units were detected for saponins in the UPLC-MS^E^ analysis, the sugar chain in this position (C-3) is considered to have one or two units. 

Disaccharides have not frequently been found in the genus *Agave* and, in fact, they have only been described for *A. utahensis* [19], for which the sugar chains β-d-glucopyranosyl-(1 → 4)-β-d-galactopyranoside (S2A) (Figure 2) and β-d-glucopyranosyl-(1 → 3)-β-d-galactopyranoside (S2B) were reported, and from the fermented leaves of *A. americana*, where a saponin with the S2A chain (Agavoside B) was described [20].

It is worth noting that structural changes or modifications at ring A of the aglycone can affect the chemical shifts of the sugar chain—mainly on the galactopyranoside [21], which has an *O*-glycosidic bond with C-3 of the aglycone. Given that the aglycones proposed in this dereplication strategy for *A. bracteosa* are H-5α, it is essential to compare the spectroscopic data of sugar residues linked to this kind of aglycone. Saponins described for *A. uthaensis* are H-5β, but a comparison of the spectroscopic data of those that have S2A and S2B sugar chains shows notable differences in the ^13^C chemical shifts of C-3 and C-4 of galactose as well as the ^1^H signal of C-1 of glucose (Table 2). These differences enable the differentiation of the two disaccharides.

Once the sugar chains of *A. bracteosa* saponins had been proposed (d-glucopyranose and S2A or S2B), the experimental data from HSQC, HSQC-TOCSY and HMBC experiments on the SF were compared with the literature spectroscopic data of saponins with these sugar chains and the most structurally related aglycones to those dereplicated for *A. bracteosa*. 

Thus, a set of concordant correlations for d-glucopyranoside units at positions C-3, C-6, and C-26, in addition to the disaccharide S2A, are found (Table 3).

The dereplication of the aglycones showed that some of them could be hydroxylated at C-2 (A6, A7). This structural feature affects the chemical shifts of d-galactose units and could also be observed in the HSQC-TOCSY spectrum of the SF. Thus, the signal at δ 4.89 ppm (Table 3) could be assigned to H-1 of galactose from an S2A chain linked to hydroxylated aglycones at C-2. The deshielding of the anomeric proton signals by +0.05 and +0.9 ppm in the ^1^H and ^13^C NMR spectra, respectively, along with the shielding of C-2 by −0.4 ppm (Table 3. Supplementary Materials, Figure S5), is consistent with the data reported [21].

Finally, the doublet at 5.01 ppm in the ^1^H NMR spectrum of the SF showed typical correlations of a monosaccharide in the two-dimensional experiments, and this signal remains unassigned. It can be proposed that this signal is due to a sugar chain of saponins with an A2 aglycone. This anomeric signal and the A2 methyl singlet at 0.97 ppm heterocorrelated with an oxygenated carbon (δ 81.3 ppm) in the HMBC spectrum, which indicates that this monosaccharide forms part of the saponin with the aglycone A2. This aspect requires further elucidation. 

### 2.5. Identification of Saponins from A. bracteosa

The main saponin observed in the UPLC-MS^E^ of the SF (Rt 6.09 min) contained paniculogenin (A1) as the aglycone with two β-glucopyranose units linked to it at the C-3 and C-6 positions. This was confirmed by the 2D-correlations of doublets at δ 5.06 ppm and δ 4.86 ppm (Table 3), which are also the two most intense anomeric signals in the ^1^H NMR spectrum. Furthermore, mass fragments observed in the MS^E^ are also consistent with the proposed structure (Figure 1). Thus, the main saponin was identified as 3,6-di-*O*-β-d-glucopyranosylpaniculogenin (**1**) (Figure 2). This compound is described here for the first time, and it is the epimer of cantalasaponin-1 at C-25 [22]. 

Saponins at Rt 1.39, 1.51, 1.89, and 4.97 min in the UPLC-MS^E^ of the SF were the only ones that possessed fragments [Agl + 162—H]^−^ at *m*/*z* 623, 625, 607 and 595, which correspond to the aglycones A6, A7, A5 and A8, respectively. All of these contain three hexose units that are consistent with d-glucopyranoside at C-26 and the sugar chain S2A linked to C-3, as previously determined in the dereplication of the sugar chains. Thus, these saponins are identified as (25*S*)-26-*O*-β-d-glucopyranosyloxy-2α,3β,22-trihydroxy-5α-9(11)-dehydrofurostan-12-one 3-*O*-β-d-glucopyranosyl-(1 → 4)-*O*-β-d-galactopyranoside (**2**), (25*S*)-26-*O*-β-d-glucopyranosyloxy-2α,3β,22-trihydroxy-5α-furostan-12-one 3-*O*-β-d-glucopyranosyl-(1 → 4)-*O*-β-d-galactopyranoside (**3**), (25*S*)-26-O-β-d-glucopyranosyloxy-3β,22-dihydroxy-5α-9(11)-dehydrofurostan-12-one 3-*O*-β-d-glucopyranosyl-(1 → 4)-*O*-β-d-galactopyranoside (**4**) and (25*S*)-26-*O*-β-d-glucopyranosyloxy-5α-furostan-3β,22-diol 3-*O*-β-d-glucopyranosyl-(1 → 4)-*O*-β-d-galactopyranoside (**5**) (Figure 2).

In addition to the above, there were two saponins with a mass fragment of *m*/*z* 609 [Agl + 162—H]^−^ and at least one of them should contain the aglycone A4. Fragments observed in the UPLC-MS^E^ analysis at 2.25 min show three hexose residues and, considering the proximity of furostan-12-one-type saponins in UPLC analysis, this can be identified as (25*S*)-26-O-β-d-glucopyranosyloxy-3β,22-dihydroxy-5α-furostan-12-one 3-*O*-β-d-glucopyranosyl-(1 → 4)-*O*-β-d-galactopyranoside (**6**).

The structures of the remaining saponins cannot be proposed, and two of the aglycones could not be dereplicated. As a result, the isolation and structural characterization of these saponins were carried out. 

### 2.6. Isolation and Structural Elucidation of Saponins from A. bracteosa

**Figure 4:**
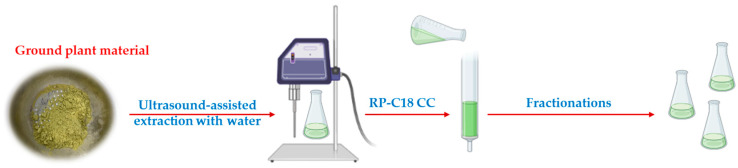
Fast and green extraction and fractionation of *A. bracteosa*.

The furostane-type saponins were obtained in good yield in the ultrasound-assisted aqueous extraction, as deduced after dereplication of the SF, and 1 g of plant material was subsequently used for the isolation of pure compounds (Figure 4).

The aqueous extract was directly loaded onto a C-18 chromatographic column, and the fractionation was carried out with mixtures of acetone:water at different concentrations as the mobile phase. Acetone was used instead of methanol to avoid the formation of methoxy derivatives. The use of methanol was also avoided during the HPLC purification for the same reason. Finally, ten saponins were isolated, namely five from those already dereplicated (**1**–**3**, **5**–**6**) (Figure 2) and another five new saponins (**7**–**11**). The dereplicated structures were confirmed by 1D- and 2D-NMR analysis and this included the *R* configuration of C-22 (Table 4 and Table 5). Compound **1**, (25*S*)-cantalasaponin-1, is described here for the first time in the literature. Regarding furostanic saponins, compound **3**, tribufuroside D, is the only one that has been reported previously—in that case, from *Tribulus terrestris* [23]. Thus, compounds **2**, **5**, and **6** are named bractofuranosides A–C.

The two isomers **7** and **8** appeared in the UPLC-MS^E^ analysis with retention times of 3.67 and 4.56 min, respectively, and they showed a molecular ion peak [M—H]^–^ of 935.48 Da and the same fragmentation pattern until the last fragment was reached [Agl + 162—H]^–^ *m*/*z* 611. This finding is in good agreement with a furostanic aglycone with a hydroxyl substituent. The application of the HMAI method proposed 5α-furostan-3β,6α,22-triol and 5α-furostan-2α,3β,22-triol as aglycones, respectively (Table 6). In the former case it is proposed that the two hydroxylated positions are glucosylated. 

The complete assignment of each of the proton and carbon signals in the ^1^H and ^13^C NMR spectra of compound **7** was achieved using the spectroscopic techniques HSQC, HMBC, COSY, and TOCSY 1D (Table 7 and Table 8). The *R* configuration of C-22 was confirmed due to the observed NOE correlation between the proton of the hydroxyl group of C-22 (6.59 ppm) with H-21 (1.28 ppm). The configuration of C-25 was confirmed by applying the Agrawal method, Δ_ab_(H_2_-26) = 0.61 ppm, and the configuration of this carbon was identified as *S*.

In the most downfield region of the spectrum, three doublets with chemical shifts of 5.13, 4.89, and 4.80 ppm were observed, and this is consistent with the presence of anomeric protons for three sugars. The TOCSY-1D spectrum contained the multiplets corresponding to the spin systems of three units of β-glucopyranose. As one would expect, it was confirmed that the three glucose units were bound to the C-3, C-6 and C-26 positions due to the correlations observed in the 1D NOE and HMBC spectra of δ 5.13 with δ 3.97 (H-3) and δ 74.9 (C-3), of δH 4.89 with δ 3.63 (H-6) and δ 79.3 (C-6), and of δ 4.80 with δ 4.08 and 3.47 (H-26) and δ 75.2 (C-26).

**Figure 5:**
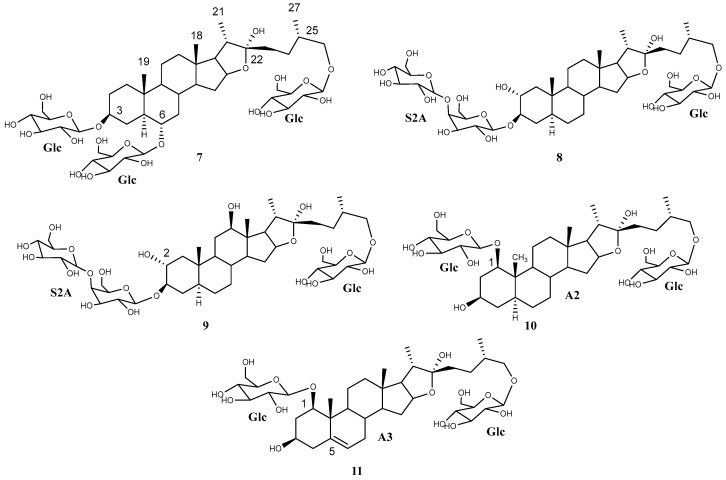
Saponins (**7**–**11**) isolated and elucidated from *A. bracteosa* extract.

Compound **7** was elucidated as (25*S*)-3,6,26-tri-(*O*-β-glucopyranosyl)-5α-furostan-3β,6α,22α,26-tetraol (Figure 5), which is described for the first time and was named bractofuranoside D. This compound is the epimer at carbon 25 of silasaponin C, which was reported in the literature in *Agave sisalana* [24].

Compound **8** had a similar ^1^H NMR profile to compounds **2**–**3** and **5**–**6**. A comparison of the ^1^H and ^13^C NMR data for the sugar chain indicated that all saponins contained the same sugar chains (Table 5 and Table 8). Therefore, compound **8** was elucidated as (25*S*)-26-*O*-β-d-glucopyranosyloxy-5α-furostan-2α,3β,22α-triol 3-*O*-β-d-glucopyranosyl-(1 → 4)-*O*-β-d-galactopyranoside (Figure 5), which is described here as a pure compound for the first time and was named bractofuranoside E. This compound was previously reported in a mixture with its epimer C-25*R*, terrestrosin F, from *Tribulus terrestris* [25].

A minor saponin, **9**, that co-eluted with compound **2** was also isolated. An exhaustive study of the NMR spectra of these compounds showed how they shared the same glycosylation while the aglycone was different. Application of the HMAI method allowed the aglycone structure to be proposed (Table 6), and this included two hydroxyl groups in positions C-2 and C-12, thus confirming that it was 5α-furostan-2α,3β,12β,22,26-pentaol. The configurations of C-22 and C-25 were the same for both saponins, and the C-12 configuration of **9** was also confirmed due to the NOE effect observed between H-12 and H-11ec, H-7ax, H-14 and H-17, all of which are located on the α side of the aglycone. Only two saponins with this aglycone have been tentatively described through UHPLC/Q-TOF MS analysis [26], and therefore, this is the first report on the isolation of a saponin with this aglycone. Finally, the structure of saponin **9** was determined as (25*S*)-26-*O*-β-d-glucopyranosyloxy-5α-furostan-2α,3β,12β,22α-tetraol 3-*O*-β-d-glucopyranosyl-(1→4)-*O*-β-d-galactopyranoside (Figure 5), known as bractofuranoside F.

Saponin **10**, at Rt 2.92 min in the UPLC-MS^E^ analysis, was isolated and showed the HMBC correlations already proposed for the aglycone A2 during the dereplication. The HMAI method (Table 6) gave partial information about the aglycone structure, and this was confirmed to be a furostanic saponin. In the ^1^H NMR spectrum of compound **10**, two doublets were observed at δ 4.79 (*J* = 8 Hz) and δ 5.04 (*J* = 8 Hz). 1D TOCSY experiments carried out by selection of these two signals gave typical subspectra of β-glucopyranose.

The HMAI method could not assign the singlet at 1.00 ppm, but because other methyl groups were assigned, it was supposed that this corresponded to C-19. In the HMBC experiment, this signal showed hetero-correlations at δ 41.6, 54.6, and 81.1. The latter correlation, with a chemical shift for an oxygenated carbon, correlated with δ 3.97 in the HSQC experiment. The only position at a distance of three bonds that could be an oxygenated methyne (–CH–) was C-1. In addition, in the ^1^H NMR spectrum, the signal 3.97 ppm appeared as a doublet of doublets (*J* = 12, 4 Hz), which is consistent with a *trans*-diaxial position with H-2_ax_. The COSY experiment showed the homo-correlations between the proton signal at δ 3.97 (H-1_ax_) and signals at δ 1.93 and δ 2.85, belonging to H-2, which in turn showed a correlation with a signal at δ 3.86 (H-3).

The full assignment of the ^1^H and ^13^C NMR signals was made (Table 7 and Table 8), and the NOESY 2D experiment showed correlations between the proton at δ 0.99 (H-5), and protons δ 3.97 (H-1_ax_), 3.86 (H-3_ax_) and 1.71 (H-4_ec_), thus confirming that they are located on the α side of the molecule. The configurations of C-22 and C-25 were the same as those of the other isolated saponins. Finally, the aglycone was (25*S*)-5α-furostan-1β,3β,22α,26-tetraol. This aglycone has not been reported previously for the genus *Agave*, and a saponin with a hydroxyl at the C-1 position has only been identified for *A. decipiens* [27].

The correlations in the HMBC and 1D NOESY experiment between proton δ 5.04 with 81.2 ppm (C-1) and 3.97 ppm (H-1) indicated that the position C-1 was glucosylated.

Compound **10** was elucidated as 1,26-di-(*O*-β-d-glucopyranosyl)-(25*S*)-5α-furostan-1β,3β,22α,26-tetraol (Figure 5), which is described here for the first time and is named bractofuranoside G.

The HMBC spectrum of compound **11** showed correlations for a furostanic saponin (Table 6) and uncommon correlations for the H-19 singlet, in agreement with the presence of an oxygenated carbon (83.1 ppm) and a double bond (139.4 ppm) and with the aglycone A3 in the dereplication. As for compound **10**, the former functionalization was assigned to C-1. The carbon signal at 139.4 ppm did not show any correlations in the HSQC experiment. This finding indicates its quaternary nature and this carbon corresponds to either C-5 or C-9, both of which are located three bonds from H-19. In the ^13^C NMR spectrum, a signal was observed at 124.8 ppm and this can be assigned to the double bond, which correlated with 5.54 ppm in the HSQC spectrum. Analysis of the COSY and TOCSY spectra placed this signal in a B ring, thus locating it between the C-5 and C-6 positions.

The complete assignment of the ^1^H NMR and ^13^C NMR spectra was performed using the spectroscopic techniques HSQC, HMBC, COSY, NOESY, and TOCSY 1D, and it was confirmed that the C-22 and C-25 configurations were the same as for furostanic saponins.

Finally, it was deduced that the aglycone of compound **11** was (25*S*)-furost-5-en-1β,3β,22α,26-tetraol. The sugar chain moieties and their positions are the same as for **10**, and compound **11** was therefore identified as 1,26-di-(*O*-β-d-glucopyranosyl)-(25*S*)-furost-5-en-1β,3β,22α,26-tetraol (Figure 5). The 22-methoxy derivative of this saponin has been described for *Dracaena surculosa* [28], although this is the first time that it has been described in its natural form, and it has been named bractofuranoside H.

### 2.7. Cytotoxicity of Isolated Saponins

The cell viabilities of the isolated saponins from the SF of *A. bracteosa* were assayed on HeLa cells at 100 µM for 24 h. Etoposide was used as the positive control. Significant activity was not observed for the saponins assayed, with the exception of compounds **5** and **10**, which displayed moderate activity. All compounds were less active than the positive control (27.6% cell viability).

The saponins that are diglucosylated at C-3 and C-6 (**1** and **7**) did not show cytotoxic activity, as previously reported in the literature for the epimer of compound **1** (cantalasaponin-1 (25*R*)) [29]. Furthermore, it has been established that either C-6 hydroxylation or *O*-glucosylation in steroidal saponins drastically reduces their cytotoxic activities [30].

The remaining compounds can be divided into two groups, one with an S2A sugar chain at C-3 (**2**, **3**, **5**, **6**, **8**, **9**) and the other with glucopyranose at C-1 (**10**, **11**). 

In the first group, compound **5** showed moderate activity since it reached a cell viability value of 57.09% ± 1.47. Nevertheless, the other saponins from this group, which have oxygenation in their aglycone (carbonyl at C-12 and hydroxyl groups at C-2 and/or C-12 positions), were inactive. This trend has previously been described for furostane saponins [31].

It is also worth mentioning the difference in activity observed for 1-glucopyranosylsaponins **10** and **11**. Compound **10** displayed certain cytotoxicity and showed a percentage cell viability of 53.39 ± 9.08 at 100 µM, whereas saponin **11** was inactive. The main difference was found in the double bond at C-5(6) in this last compound, and this feature led to a drastic loss of activity. 

## 3. Materials and Methods

### 3.1. General Experimental Procedures

Optical rotations were measured on a JASCO p-2000 polarimeter using methanol as solvent. Accurate mass was measured on a UPLC-QTOF ESI (WatersXevo G2, Manchester, UK) high-resolution mass spectrometer (HRESI-TOFMS). The 1D and 2D NMR spectra were recorded on Agilent INOVA-600 and Bruker AVANCE NEO 700 MHz spectrometers with a 5 mm helium-cooled cryoprobe. Pyridine-*d*_5_ (Eurisotop Saint Aubin, Saint-Aubin, France) was used as a reference solvent, and ^1^H and ^13^C NMR experiments were carried out at 25 °C. The chemical shifts are given on the δ scale and are referred to as the residual pyridine signals (δH 8.70, 7.55, 7.18 and δC 149.84, 135.60, 123.48). *n*-Butanol was supplied by Panreac Química S.A. (Castellar del Vallés, Barcelona, Spain). Methanol, acetone, and acetonitrile were obtained from VWR International (Radnor, PA, USA). SPE Strata-X 33 µm polymeric reversed-phase cartridges (Phenomenex) were used to obtain the SFs. TLC silica 60 F254 and TLC Si gel F254S RP-18 plates were purchased from Merck (Darmstadt, Germany) and were used to monitor the isolation processes. The compounds were visualized by spraying the plate with H_2_SO_4_/H_2_O/AcOH (4:16:80 *v*/*v*/*v*). LiChroprep RP-18 (40–63 μm) from Merck (Darmstadt, Germany) was used for vacuum column chromatography for the first fractionation. Further purification was carried out using analytical HPLC using Kromasil RP-18 (10 lm, 250 4.6 mm i.d., Phenomenex Ltd., Aschaffenburg, Germany). Preparative TLC silica gel 60 F_254_ (0.25 mm) was also used, and they were supplied by Merck (Darmstadt, Germany).

### 3.2. Plant Material 

Leaves of *Agave bracteosa* S. Watson (1039 g) were supplied in March 2023 by Desert City S.L. (CIF B86691474, Madrid, Spain). Reference samples of powdered plant material, *n*-ButOH, and water extracts are available in our laboratory at the University of Cadiz, Puerto Real, and are labeled DC2023-M1.

### 3.3. Extraction of Plant Material

Leaves from *A. bracteosa* were washed with ethanol and they were then sliced up and dried in an oven at 50 °C until a constant weight was obtained. The dried leaves were then ground in a mill to give 300.6 g of material. 

#### 3.3.1. Saponin Extraction with Water

In total, 500 mg of dried plant material was extracted for 10 min by ultrasound and by addition of 20 mL of water (ratio 40:1 (*v*/*w* water:plant material)). After the extraction, the vial was centrifuged to obtain the supernatant and the solvent was removed on a rotatory evaporator to give the crude extract.

#### 3.3.2. Saponin Extraction with *n*-Butanol:Water 1:1

Dried plant material was extracted according to the protocol reported by Durán et al. 2021 [11]. A total of 500 mg of plant material was first moistened for 2 h with water in a ratio 2:1 (*v*/*w*, water:plant material). *n*-Butanol was then added in a 1:1 ratio (*v*/*v*) water:*n*-butanol to give the biphasic solvent *n*-butanol:water. After maceration for 24 h at room temperature, the recovery of the organic phase was achieved by increasing the volume of water, and the sample was gently agitated magnetically for a further 24 h. Finally, the supernatant was collected and the two phases were separated in a separating funnel. The solvent was removed from the organic phase under vacuum.

#### 3.3.3. Saponin-Rich Fraction (SF) Preparation

Ultrasound-assisted water extraction and *n*-butanol:water 1:1 maceration extracts (26 and 32 mg, respectively) were dissolved in 4 mL of deionized water and then chromatographed on a C-18 SPE cartridge previously conditioned with 10 mL of methanol and 10 mL of deionized water. The eluents utilized were 4 mL of deionized water (to obtain the sugar fraction), 4 mL of methanol:water 8:2 (to obtain the enriched saponin fraction), and 8 mL of methanol (to obtain a less polar fraction). Yields from the dry weight of *A. bracteosa* were 26.6% and 5.5%, respectively.

#### 3.3.4. Extraction of Plant Material and Isolation of Pure Compounds

Extraction of 1 g of leaves of *A. bracteosa* was carried out using the ultrasound-assisted water extraction technique. 

The aqueous extract (40 mL) was directly loaded onto a C18 column, which was previously conditioned with 300 mL of methanol and 300 mL of water. Subsequently, 50 mL samples were collected in vials using the following mobile phases: 350 mL of water (vials 1–7), 450 mL of 15% acetone:water (vials 8–16), 250 mL of 20% acetone:water (vials 17–21), 1100 mL of 25% acetone:water (vials 22–43), 550 mL of 30% acetone:water (vials 44–54), 450 mL of 35% acetone:water (vials 55–63), 250 mL of 40% acetone:water (vials 64–68), 400 mL of 50% acetone:water (vials 69–76). The following fractions were obtained by TLC: A (vials 17–22), B (vials 24–28), C (vials 30–36), D (vials 47–55), E (vials 64–68), and F (vials 69–76). Fraction A (57.0 mg) was subjected to RP-18 HPLC using ACN:H_2_O (2:8) as the mobile phase to give pure compounds **2** (2.0 mg), **3** (2.0 mg), and fraction A_1_ (8.7 mg). This fraction was purified by preparative TLC on silica gel 60 F_254_ plates with butanol:acetic acid:water (5:1:5) as the solvent, which led to the isolation of compound **9** (2.0 mg). Fraction B (59.6 mg), purified by RP-18 HPLC using ACN:H_2_O (2:8), gave pure compound **6** (2.6 mg). Under the same conditions, Fraction C (56.8 mg) was chromatographed by RP-18 HPLC to give pure compounds **10** (3.3 mg) and **11** (1.0 mg). Fraction D (48.8 mg) was subjected to RP-18 HPLC using ACN:H_2_O (3:7) to give compounds **7** (5.2 mg) and **8** (2.0 mg). In the same way, fraction E (18.0 mg) gave compound **5** (1.9 mg). Finally, fraction F gave compound **1** (44.0 mg) in pure form.

### 3.4. UPLC-QTOF/MS^E^ Analysis

The stock solutions (1000 ppm) of the saponin-rich fractions (SF) were prepared in water:acetonitrile (6:4). All samples were injected as a 1:15 dilution (66.7 ppm) and filtered through a PTFE syringe filter (0.22 μm) prior to analysis.

In accordance with a methodology described in a previous publication [32], SF solutions (5 μL) were injected into an Acquity UPLC HSS T3 1.8 μm, 2.1 × 5 mm VanGuard precolumn attached to an Acquity UPLC HSS T3 1.8 μm, 2.1 × 100 mm column, maintained at 45 °C. The mobile phase consisted of H_2_O (A) and CH_3_CN (B), each containing 0.1% (*v*/*v*) formic acid, with the following gradient: 0–1.50 min, 95% A; 1.50–3.0 min, 95–80% A; 3.0–6.0 min, 80–50% A; 6.0–7.0 min, 50–5% A; 7.0–7.5 min, 5% A, 7.5–8 min, 5–95% A, and maintenance in 95% A (8.0–10.0 min) to condition the column for the next injection. The flow rate was 0.4 mL/min. The temperature in the autosampler was set at 10 °C. 

Electrospray ionization in the negative polarity mode (ESI^–^) was used with the following settings: sample probe capillary voltage 2800 V, sampling cone voltage 30 V, source temperature 120 °C, and desolvation temperature 450 °C. Desolvation and cone gas with flow rates of 850 and 10 L/h were used, respectively. The data were acquired in the centroid mode using MS^E^ (low collision energy, 6 eV; high collision energy ramp, 20–80 eV) over a mass range of *m*/*z* 100–2000 and a retention time range of 0–10.0 min with a 0.5 s scan time. Data acquisition and processing were carried out with MassLynx version 4.1 (Waters Inc. Milford, MA, USA, 2013). 

### 3.5. Identification of Aglycones Using HMAI (HMBC Method for Aglycone Identification) Method

Two flowcharts were used as a tool for the identification of the aglycones of the saponins from *A. bracteosa*, one for the singlet signals and one for the doublet signals, together with tables regarding the HMBC correlations for the different structural characteristics [12]. The decisions (inside diamonds) are denoted with D or S and a number, and they use both ranges of chemical shifts and absolute values in the flowchart. In the latter case, values within the error range established (±0.4 ppm for ^13^C NMR and ±0.04 ppm for ^1^H NMR) for signals were considered. The flowchart for the methyl singlets provides information on rings A–E, while the other one provides information on rings C–F. In some cases, the flowchart indicated that the HMBC values for a specific methyl should be revised. In this situation, taking into consideration the structural features obtained, data tables should be used. If the HMBC correlations did not adjust with data tables, the saponin has other structural characteristics that will require isolation and structural elucidation.

### 3.6. NMR and UPLC-MS^E^ Data of Pure Compounds

**(25*S*)-Cantalasaponin-1 (1)**. [α]_Na_^25^−15.4 (c 0.26, MeOH); ^1^H and ^13^C NMR, see Table 4 and Table 5; UPLC-HRESIMS (negative ion mode) Rt 6.09 min, *m*/*z* 817.4237 [M + CH_3_COO^−^]^−^ (calcd for C_40_H_65_O_17_, 817.4222); MS^E^ *m*/*z* 771 [M—H]^−^, 609 [M—H—162]^−^.

**Bractofuranoside A (2)**. [α]_Na_^25^–11.9 (c 0.20, MeOH); ^1^H and ^13^C NMR, Table 4 and Table 5; UPLC-HRESIMS (negative ion mode) Rt 1.39 min, *m*/*z* 993.4540 [M + CH3COO^−^]^−^ (calcd for C_46_H_73_O_23_, 993.4543); MS^E^ *m*/*z* 947 [M—H]^−^, 785 [M—H—162]^−^, 623 [M—H—162 × 2]^−^.

**Bractofuranoside B (5)**. [α]_Na_^25^–12.96 (c 0.28, MeOH); ^1^H and ^13^C NMR, Table 4 and Table 5; UPLC-HRESIMS (negative ion mode) Rt 4.97 min, *m*/*z* 965.4961 [M + CH_3_COO^−^]^−^ (calcd for C_46_H_77_O_21_, 965.4957); MS^E^ *m*/*z* 919 [M—H]^−^, 757 [M—H—162]^−^, 595 [M—H—162 × 2]^−^.

**Bractofuranoside C (6)**. [α]_Na_^25^–3.5 (c 0.30, MeOH); ^1^H and ^13^C NMR, see Table 4 and Table 5; UPLC-HRESIMS (negative ion mode) Rt 2.25 min, *m*/*z* 979.4761 [M + CH_3_COO^−^]^−^ (calcd for C_46_H_75_O_22_, 979.4750); MS^E^ *m*/*z* 933 [M—H]^−^, 771 [M—H—162]^−^, 609 [M—H—162 × 2]^−^.

**Bractofuranoside D (7)**. [α]_Na_^25^–12.1 (c 0.45, MeOH); ^1^H and ^13^C NMR, see Table 7 and Table 8; UPLC-HRESIMS (negative ion mode) Rt 3.67 min, *m*/*z* 981.4924 [M + CH_3_COO^−^]^−^ (calcd for C_46_H_77_O_22_, 981.4906); MS^E^ *m*/*z* 935 [M—H]^−^, 773 [M—H—162]^−^, 611 [M—H—162 × 2]^−^.

**Bractofuranoside E (8)**. [α]_Na_^25^–10.7 (c 0.14, MeOH); ^1^H and ^13^C NMR, Table 7 and Table 8; UPLC-HRESIMS (negative ion mode) Rt 4.56 min, *m*/*z* 981.4922 [M + CH_3_COO^−^]^−^ (calcd for C_46_H_77_O_22_, 981.4906); MS^E^ *m*/*z* 935 [M—H]^−^, 773 [M—H—162]^−^, 611 [M—H—162 × 2]^−^.

**Bractofuranoside F (9)**. [α]_Na_^25^–2.1 (c 0.25, MeOH); ^1^H and ^13^C NMR, Table 7 and Table 8; UPLC-HRESIMS (negative ion mode) Rt 1.35 min, *m*/*z* 997.4871 [M + CH_3_COO^−^]^−^ (calcd for C_46_H_77_O_23_, 997.4856); MS^E^ *m*/*z* 951 [M—H]^−^, 789 [M—H—162]^−^, 627 [M—H—162 × 2]^−^.

**Bractofuranoside G (10)**. [α]_Na_^25^–16.1 (c 0.28, MeOH); ^1^H and ^13^C NMR, see Table 7 and Table 8; UPLC-HRESIMS (negative ion mode) Rt 2.92 min, *m*/*z* 819.4384 [M + CH_3_COO^−^]^−^ (calcd for C_40_H_67_O_17_, 819.4378); MS^E^ *m*/*z* 773 [M—H]^−^, 611 [M—H—162]^−^.

**Bractofuranoside H (11)**. [α]_Na_^25^–2.7 (c 0.17, MeOH); ^1^H and ^13^C NMR, Table 7 and Table 8; UPLC-HRESIMS (negative ion mode) Rt 3.11 min, *m*/*z* 817.4225 [M + CH_3_COO^−^]^−^ (calcd for C_40_H_65_O_17_, 817.4222); MS^E^ *m*/*z* 771 [M—H]^−^, 609 [M—H—162]^−^.

### 3.7. Cytotoxicity Assay on HeLa Cells

The cytotoxic activity of ten pure saponins was evaluated on human cervix carcinoma (HeLa) cells using the ab112118 cell cytotoxicity assay kit (Assay Solution) (Abcam, Cambridge, UK) to assess cell viability. 

HeLa cells were cultured as monolayers in DMEM (purchased from GIBCO (Paisley, UK)) supplemented with 10% fetal bovine serum, 5% glutamine, 5% nonessential amino acids, 5% penicillin, streptomycin, and 5% sodium pyruvate. Cells were maintained in a HERA Cell 150i (Thermo Scientific, Waltham, MA, USA) incubator at 37 °C, 5% CO_2_ and 95% humidity. 

Briefly, the cells were seeded at a density of 15,000 cells/well for 24 h. Cells were then treated with products dissolved in 0.1% DMSO at 100 µM for 24 h. After the treatment, cells were incubated with 20 µL/well of Assay Solution for 3 h at 37 °C, and the absorbance intensities at 570 nm and 605 nm were measured. The ratio of OD570/OD605 is proportional to the number of live cells. The viability of the cells was measured following the formula described in the literature [33]. Etoposide was used as a positive control at the same concentration. 

All experiments were performed at least in triplicate. The results are expressed as the mean ± S.D.

## 4. Conclusions

The dereplication of an *Agave bracteosa* extract revealed that it has a high content of steroidal-type saponins. The extraction was simplified with the use of water as a solvent, and this eliminated the need for a concentration step until the first separation. This new method provided promising results in terms of yield and a greater quantity of furostanic-type saponins extracted. The method for dereplication led to the identification of six saponins, five of which are reported for the first time, although their structures match characteristics previously described for the genus *Agave*. The saponins of *A. bracteosa* had a C-25*S* configuration, which is not common in this genus. After the purification process, five of the saponins were isolated, and their previous dereplication was confirmed. In addition, another five saponins were obtained and elucidated. Two of them had aglycones with novel structural characteristics in the genus: in this case, it is interesting to note that the HMAI method did not offer false positives and is proposed to carry out the structural elucidation. Overall, nine new saponins were isolated, namely (25*S*)-cantalasaponin-1 and bractofuranosides A–H. The cytotoxic activity of the saponins was proven to be generally non-toxic. 

## Figures and Tables

**Table 1:** HMBC correlations for methyl groups of the SF from *A. bracteosa* and HMAI information.

^1^H NMR Signal	HMBC Correlations				Methyl Assig.	Flowchart Information	Aglycone
Major signals							
1.15 d	36.1	62.4	112.4 (D5)		C21	Check C21	A1
1.09 d	30.5	36.0 (D13)	64.3		C27	Structural elucidation	A1
0.92 s	41.2	56.4	62.4 (S11)		C18	Check C18 → C6 OGlcα	A1
0.59 s *	37.0	50.7 (S13)	53.8 (S9)		C19	C3 OGlcβ; C6 OGlcα	A1
Minor signals							
1.54 d (D7)	41.7	55.4 (D6)	111.0 (D5)		C21	F C12 CO	A4–A7
1.50 d (D7)	41.2	55.1 (D6)	110.8 (D5)		C21	F C12 CO	A4–A7
1.24 d	40.6	63.9	110.8 (D5)		C21	Check C21 → F	A2, A3, A8
1.21 s	42.7	50.2	83.3	139.5 (S11)	-	Check C18 → Structural eluc.	A2
1.09 d	40.6	64.3	112.7 (D5)		C21	Check C21 → FM	A2, A3, A8
1.08 s (S2)	-	55.6	213.0 (S1)		C18	F C12 CO	A4
1.03 s	-	-	204.7 (S3)		C18	C9DB C12CO	A5
1.01–0.98 d (D2)	28.2(D1)	34.4	75.1 (D3)		C27	FM	A2–A8
0.97 s	41.4	43.0	54.9	81.3 (S11)	-	Check C18 → Structural eluc.	A3
0.88 s	-	-	170.5 (S4)		C19	H-5α C2OHα C9DB C12CO	A7
0.85 s	40.7	56.7	63.9 (S11)		C18	Check C18 → F	A8
0.79–0.75 s	40.7	56.7	64.5 (S11)		C18	Check C18 → FM	A8
0.70 s	37.3	44.8	55.4 (S9)		C19	H-5α Check C19 → C12CO	A4
0.67 s	37.0	44.8	54.4 (S9)		C19	H-5α Check C19	A8

* With a signal in the ^1^H NMR spectrum at 3.34 ppm; brd; 12 Hz (S15). Flowchart information: F furostanic, FM methoxylated furostanic structure, DB double bond, CO carbonyl group, OH hydroxyl group, OGlc glucopyranosyloxy group, α, β chiral center configuration, C# position in the aglycone, D# and S# decisions in the doublet or singlet flowchart.

**Table 2:** Literature HSQC-TOCSY data for anomeric positions of sugar chains S2A and S2B *O*-bonded at C-3 of saponins H-5α (*) and H-5β (^†^).

NMR Data	S2A * [19]	S2A ^†^ [20]	S2B ^†^ [20]
** Gal-1 **	** 4.85 **	** 4.76 **	** 4.85 **
** Gal-1 **	102.6	103.6	103.1
** Gal-2 **	73.5	73.5	71.7
** Gal-3 **	75.5	75.2	85.1
** Gal-4 **	80.0	80.1	69.9
** Glc-1 **	** 5.26 **	** 5.27 **	** 5.44 **
** Glc-1 **	107.1	107.1	106.7
** Glc-2 **	75.1	75.9	75.9
** Glc-3 **	78.5	78.7	78.4
** Glc-4 **	72.4	72.3	71.5
** Glc-5 **	78.7	78.5	78.7
** Glc-6 **	63.2	63.1	62.6

**Table 3:** Comparison of the literature NMR data for d-glucopyranoside units linked at C-3, C-6, and C-26, and the disaccharide S2A linked at C-3 with those obtained for the SF of *A. bracteosa*.

NMR Data	HSQC-TOCSY SF Data	Agavoside B [19]	25*S*-Furostane Saponin [21]	Cantalasaponin-1 [22]	HMBC SF Data
**S2A**					
**Gal-1 (S2)**	4.84/4.89 *	**4.85**			76.9/84.6 (C-3Agl)
**Gal-1 (S2)**	102.3/103.2	102.6			
**Gal-2 (S2)**	73.2/72.8	73.5			
**Gal-3 (S2)**	75.3/75.0	75.5			
**Gal-4 (S2)**	79.6/80.0	80.0			
**Gal-6 (S2)**	60.7	61.1			
**Glc-1 (S2)**	5.26/5.24 *	**5.26**			80.2 (C-4Gal)
**Glc-1 (S2)**	106.8	107.1			
**Glc-2 (S2)**	75.6	75.1			
**Glc-3 (S2)**	78.4	78.5			
**Glc-4 (S2)**	72.0	72.4			
**Glc-5 (S2)**	78.4	78.7			
**Glc-6 (S2)**	62.6–63.3	63.2			
**Glc-1 (C-26)**	**4.81–4.77**		**4.81**		75.3 (C-26 Fur)
**Glc-1 (C-26)**	104.9		105.2		
**Glc-2 (C-26)**	75.0		75.3		
**Glc-3 (C-26)**	78.3		78.7		
**Glc-4 (C-26)**	71.4		71.7		
**Glc-5 (C-26)**	78.3		78.4		
**Glc-6 (C-26)**	62.6–63.3		62.9		
**Glc-1 (C-3)**	5.06			5.11	76.9 (C-3 Agl)
**Glc-1 (C-3)**	101.3			101.6	
**Glc-2 (C-3)**	75.2			75.4	
**Glc-3 (C-3)**	78.1			78.6	
**Glc-4 (C-3)**	71.4			71.7	
**Glc-5 (C-3)**	78.1			78.0	
**Glc-6 (C-3)**	62.3			62.6	
**Glc-1 (C-6)**	4.83			4.86	80.0 (C-6 Agl)
**Glc-1 (C-6)**	106.0			106.1	
**Glc-2 (C-6)**	75.3			75.6	
**Glc-3 (C-6)**	78.3			78.4	
**Glc-4 (C-6)**	71.6			71.7	
**Glc-5 (C-6)**	78.3			77.9	
**Glc-6 (C-6)**	62.6–63.3			62.9	

* Pairs of values are for saponins without and with hydroxyl groups in aglycone at C-2.

**Table 4:** ^13^C and ^1^H NMR data (*J* in Hz) for the aglycone moieties of compounds **1**–**3**, **5**, **6** (pyridine-*d*_5_) ^a,b^.

	(25*S*)-Cantalasaponin-1 (1)		Bractofuranoside A (2)		Tribufuroside D (3)		Bractofuranoside B (5)		Bractofuranoside C (6)	
	*δ*_C_	*δ*_H_	*δ*_C_	*δ*_H_	*δ*_C_	*δ*_H_	*δ*_C_	*δ*_H_	*δ*_C_	*δ*_H_
1_ax_	37.6	0.78 ddd (13, 13, 4)	43.7	1.54 dd (12, 12)	44.9	1.08 (o)	37.3	0.78 ddd (14, 14, 3)	36.4	0.71 ddd (13, 13, 4)
1_eq_		1.41 (o)		2.23 dd (13, 5)		2.03 (o)		1.52 brd (14)		1.31 (o)
2_ax_	29.9	1.63 (o)	72.4	4.01 ddd (12, 9, 5)	69.9	3.90 (o)	30.1	1.59 ddd (14, 13, 11, 4)	29.8	1.53 dddd (13, 13, 11, 4)
2_eq_		2.00 d (13)		-		-		2.02 brd (13)		1.98 brd (13)
3	77.0	3.95 dd (11, 3)	83.8	3.81 ddd (11, 9, 5)	84.2	3.79 ddd (11, 9, 5)	77.3	3.93 dddd (12, 11, 6, 6)	77.0	3.86 dddd (11, 11, 5, 5)
4_ax_	28.6	1.41	33.8	1.46 ddd (12, 12, 12)	33.6	1.41 ddd (13, 13, 11)	34.9	1.32 ddd (12, 12, 12)	34.7	1.30 ddd (13, 12, 11)
4_eq_		3.36 d (12)		1.89 brd (12)		1.83 ddd (13, 5, 3)		1.78 brd (12)		1.79 brd (13)
5	50.9	1.21 ddd (13, 11, 3)	42.6	1.18 m	44.2	0.95 m	44.7	0.88 brdd (12, 12)	44.5	0.84 brdd (12, 12)
6_ax_	80.1	3.52 ddd (11, 11, 5)	27.2	1.24 dddd (13, 13, 13, 3)	27.6	1.06 dddd (13, 13, 13, 4)	29.1	1.10 (2 H)	28.7	1.11 (2 H)
6_eq_		-		1.18 brd (12)		1.14 brd (13)				
7_ax_	41.6	1.17	32.6	0.88 (o)	31.4	0.73 dddd (13, 13, 12, 4)	32.5	0.77 dddd (13, 12, 11, 4)	31.8	0.74 (o)
7_eq_		2.58 ddd (13, 4, 4)		1.74 m		1.55 (o)		1.51 brd (13)		1.54 (o)
8	34.0	1.50	36.3	2.37 m	33.5	1.71 dddd (11, 11, 11, 6)	35.4	1.40 (o)	34.4	1.73 dddd (11, 11, 11, 4)
9	53.8	0.50 ddd (11, 11, 4)	170.5	-	55.2	0.98 m	54.5	0.49 ddd (13, 11, 4)	55.6	0.88 ddd (13, 11, 5)
10	36.7	-	40.6	-	37.0	-	35.9	-	38.1	-
11_ax_	21.2	1.12	120.3	5.95 d (2)	37.9	2.41 dd (14, 14)	21.4	1.21 dddd (13, 13, 7, 7)	37.2	2.23 dd (14, 5)
11_eq_		1.33 d (13)				2.35 dd (14, 5)		1.38 (o)		2.38 dd (14, 14)
12_ax_	40.4	1.02	204.5	-	212.4	-	40.3	1.05 (o)	213.0	-
12_eq_		1.65						1.69 brd (12)		
13	41.3	-	51.8	-	55.5	-	41.2	-	55.8	-
14	56.4	1.01	52.7	1.70 ddd (14, 10, 6)	55.4	1.35 ddd (12, 12, 6)	56.5	1.02 ddd (14, 11, 6)	55.9	1.35 ddd (14, 11, 6)
15_a_	32.0	1.42 (o)	32.1	1.61 ddd (13, 13, 7)	31.4	1.55 ddd (13, 13, 6)	32.5	1.39 ddd (14, 12, 7)	31.8	1.57 ddd (14, 13, 6)
15_b_		1.95 ddd (12, 7, 6)		2.14 ddd (12, 7, 6)		2.05 ddd (12, 6, 6)		2.00 ddd (12, 7, 7)		2.06 ddd (13, 7, 7)
16	81.7	4.47 ddd (9, 7, 7)	80.3	4.88 ddd (9, 7, 7)	79.5	4.85 ddd (8, 8, 7)	81.2	4.92 ddd (9, 7, 7)	77.8	4.85 ddd (9, 7, 7)
17	62.4	1.81 dd (9, 7)	55.3	2.75 dd (9, 7)	54.8	2.86 dd (9, 7)	64.1	1.91 dd (9, 6)	55.1	2.88 dd (9, 7)
18	16.9	0.93 s	15.5	1.03 s	16.0	1.09 s	16.8	0.86 s	16.3	1.10 s
19	13.4	0.60 s	19.4	0.90 s	12.6	0.73 s	12.4	0.64 s	11.8	0.66 s
20	36.2	2.95 dq. (7, 7)	41.7	2.27 dq (7, 7)	41.1	2.18 dq. (7, 7)	40.8	2.21 dq (6, 7)	41.4	2.18 dq (7, 7)
21	14.6	1.16 d (7)	15.1	1.56 d (7)	15.0	1.51 d (7)	16.6	1.30 d (7)	15.3	1.52 d (7)
22	112.5	-	111.0	-	110.5	-	110.7	-	110.8	-
23_a_	63.4	ax4.03 ddd (12, 11, 5)	37.1	1.96 ddd (12, 12, 4)	36.9	1.95 ddd (13, 13, 4)	37.3	1.95 ddd (12, 12, 3)	36.7	1.95 ddd (12, 12, 3)
23_b_		-		2.09 ddd (12, 12, 4)		2.05 ddd (13, 12, 4)		2.07 ddd (12, 12, 4)		2.06 ddd (12, 12, 3)
24_a_	36.1	ax2.20 ddd (12, 12, 5)	28.4	1.67 dddd (12, 12, 8, 4)	28.1	1.66 m	28.4	1.67 m	28.4	1.66 m
24_b_		eq1.90 ddd (12, 5, 3)		2.03 m		2.03 m		2.04 m		2.04 m
25	30.6	1.83 m	34.5	1.91 m	34.2	1.91 m	34.5	1.91 m	34.5	1.91 m
26_a_	64.3	ax3.97 dd(11, 3)	75.3	3.48 dd (9, 7)	75.1	3.48 dd (9, 7)	75.3	3.46 dd (9, 7)	75.4	3.48 dd (9, 7)
26_b_		eq 3.29 brd (11)		4.06 dd (9, 6)		4.08 dd (9, 6)		4.07 dd (9, 6)		4.06 dd (9, 6)
27	17.6	1.10 d (7)	17.6	1.01 d (7)	17.2	1.00 d (7)	17.6	1.01 d (7)	17.6	1.01 d (7)

^a^ Assignments were confirmed by ^1^H-^1^H-COSY, 1D and 2D-TOCSY, HSQC, HSQC-TOCSY and HMBC experiments. ^b^ o: overlapped with other signals.

**Table 5:** ^13^C and ^1^H NMR data (*J* in Hz) for the sugar chains of compounds **1**–**3**, **5**, **6** (pyridine-*d*_5_) ^a,b^.

	(25*S*)-Cantalasaponin-1 (1)		Bractofuranoside A (2)		Tribufuroside D (3)		Bractofuranoside B (5)		Bractofuranoside C (6)	
	*δ*_C_	*δ*_H (C-H)_	*δ*_C_	*δ*_H (C-H)_	*δ*_C_	*δ*_H (C-H)_	*δ*_C_	*δ*_H (C-H)_	*δ*_C_	*δ*_H (C-H)_
C3										
		β-d-Glc		β-d-Gal		β-d-Gal		β-d-Gal		β-d-Gal
1	101.7	5.09 d (8)	103.5	4.90 d (8)	103.5	4.90 d (8)	103.6	4.88 d (8)	102.6	4.86 d (8)
2	75.6	4.04 dd (8, 9)	73.1	4.45 dd (8, 8)	72.9	4.44 dd (8, 8)	73.2	4.37 dd (9, 9)	73.6	4.37 dd (8, 8)
3	78.7	4.23 (o)	75.9	4.25 m	74.9	4.24 m	75.3	4.25 dd (10, 4)	75.5	4.23 dd (7, 4)
4	71.8	4.24 (o)	80.3	4.67 dd (4, 2)	80.3	4.67 dd (4, 2)	80.2	4.71 dd (4, 2)	80.2	4.70 dd (4, 2)
5	78.2	3.81 ddd (8, 6, 3)	76.1	4.11 dd (7, 7)	76.1	4.11 dd (7, 7)	76.1	4.13 dd (9, 9)	75.6	4.08 dd (7, 7)
6	63.2	4.42 dd (12, 3)	61.0	4.62 m	60.9	4.62 m	61.0	4.67 dd (12, 3)	61.2	4.66 (o)
		4.27 dd (12, 5)		4.26 (o)		4.26 (o)		4.28 dd (12, 5)		4.27 (o)
1				β-d-Glc		β-d-Glc		β-d-Glc		β-d-Glc
2			107.3	5.26 d (8)	107.3	5.26 d (8)	107.3	5.28 d (8)	107.2	5.28 d (8)
3			75.8	4.13 dd (8, 9)	75.8	4.13 dd (8, 9)	75.5	4.13 dd (8, 9)	76.1	4.13 dd (8, 9)
4			78.7	4.23 (o)	78.6	4.22 dd (9, 9)	78.8	4.23 (o)	78.8	4.21 dd (9, 9)
5			71.8	4.07 dd (10, 9)	72.1	4.07 dd (10, 9)	72.4	4.07 dd (10, 6)	72.4	4.06 dd (10, 9)
6			78.7	4.00 dd (10, 7, 3)	78.3	4.00 dd (10, 7, 3)	78.6	4.02 (o)	78.6	4.01 ddd (10, 7, 3)
			63.2	4.57 dd (12, 3)	63.2	4.57 dd (12, 3)	63.2	4.59 dd (12, 5)	63.2	4.59 dd (12, 3)
				4.21 dd (12, 5)		4.20 dd (12, 5)		4.22 dd (12, 3)		4.20 dd (12, 5)
C6/C26										
		β-d-Glc		β-d-Glc		β-d-Glc		β-d-Glc		β-d-Glc
1	106.4	4.85 d (8)	105.3	4.80 d (8)	105.3	4.80 d (8)	105.3	4.80 d (8)	105.2	4.80 d (8)
2	75.7	4.03 dd (8, 8)	75.3	4.01 dd (9, 8)	75.1	4.01 dd (8, 8)	75.8	4.02 dd (9, 8)	75.3	4.01 dd (8, 8)
3	78.5	4.23 (o)	78.6	4.22 (o)	78.4	4.21 (o)	78.7	4.23 (o)	78.4	4.22 (o)
4	71.9	4.24 (o)	71.8	4.21 (o)	71.5	4.22 (o)	71.8	4.22 (o)	71.8	4.21 (o)
5	78.1	3.95 ddd (8, 6, 3)	78.8	3.92 ddd (8, 6, 3)	78.3	3.92 ddd (8, 6, 3)	78.6	3.92 ddd (10, 7, 3)	78.7	3.92 ddd (8, 6, 3)
6	62.7	4.54 dd (12, 3)	62.9	4.53 dd (12, 3)	62.9	4.53 dd (12, 3)	62.9	4.53 dd (12, 3)	62.9	4.53 dd (12, 3)
		4.40 m		4.37 dd (12,5)		4.38 dd (12, 5)		4.39 dd (12, 5)		4.37 dd (12, 5)

^a^ Assignments were confirmed by ^1^H-^1^H-COSY, 2D-TOCSY, HSQC, HSQC-TOCSY and HMBC experiments. ^b^ o: overlapped with other signals.

**Table 6:** Correlations between methyl groups and nearby carbons found in the HMBC spectra of pure compounds **7**–**11** isolated from *A. bracteosa.* HMAI information is also provided.

^1^H NMR Signal	HMBC Correlations				Methyl Assig.	Flowchart Information
Saponin **7**						
1.28 d	40.7	63.7	110.4 (D5)		C21	Check C21 → F
1.02 d	28.1 (D1)	34.3	75.3 (D3)		C27	F
0.80 s (S12)	40.7	56.2	63.7 (S11)		C18	Check C18 → C6 OGlcα
0.70 s *	37.7	51.7 (S13)	53.1 (S9)		C19	C3 OGlcβ; C6 OGlcα
Saponin **8**						
1.29 d	40.7	63.9	110.6 (D5)		C21	Check C21 → F
1.01 d	28.1 (D1)	34.3	75.3 (D3)		C27	F
0.84 s	40.8	56.2	63.9 (S11)		C18	Check C18 → F
0.70 s **	36.8	45.0	54.4 (S9)		C19	Check C19 H-5α C2OHα
Saponin **9**						
1.58 d	41.8	63.9	110.9 (D5)		C21	Check C21 → F
0.99 d	28.7 (D1)	34.6	75.6 (D3)		C27	F
1.12 s	47.0	55.0	63.6	79.4 (S8)	C18	Check C18 → C12 OHβ
0.73 s **	37.0	45.2	53.7 (S9)		C19	Check C19 H-5α C2OHα
Saponin **10**						
1.26 d	40.8	64.0	110.6 (D5)		C21	Check C21 → F
0.99 d	28.3 (D1)	34.5	75.3 (D3)		C27	F
1.00 s	41.6	54.6	81.1 (S11)		C19	Check C18 → Structural elu.
0.89 s	40.7	56.6	64.0 (S11)		C18	Check C18 → F
Saponin **11**						
1.26 d	40.8	63.9	110.6 (D5)		C21	Check C21 → F
1.00 d	28.3 (D1)	34.5	75.3 (D3)		C27	F
1.24 s	42.8	50.3	83.1 (S11)	139.4	C19	Check C18 → Structural elu.
0.93 s	40.5	56.9	64.0 (S11)		C18	Check C18 → F

* 3.40 ppm brd (12 Hz) (S15) ** H-1Gal 4.91/4.90 ppm (S19). Flowchart information: F furostanic structure, OH hydroxyl group, OGlc: glucopyranosyloxy group, α, β chiral center configuration, C# position in the aglycone, D#, and S# decisions in the doublet or singlet flowchart.

**Table 7:** ^13^C and ^1^H NMR data (*J* in Hz) for the aglycone moieties of compounds **7**–**11** (pyridine-*d*_5_) ^a,b^.

	Bractofuranoside D (7)		Bractofuranoside E (8)		Bractofuranoside F (9)		Bractofuranoside G (10)		Bractofuranoside H (11)	
	*δ*_C_	*δ*_H_	*δ*_C_	*δ*_H_	*δ*_C_	*δ*_H_	*δ*_C_	*δ*_H_	*δ*_C_	*δ*_H_
1_ax_	37.6	0.81 ddd (14, 12, 4)	45.8	1.12 dd (12, 12)	45.7	1.12 (o)	81.2	3.97 dd (12, 4)	83.3	3.93 dd (12,4)
1_eq_		1.51 brdd (14, 3)		2.20 dd (13, 5)		2.20 dd (13, 5)		-		-
2_ax_	28.7	1.67 brddd (13, 12, 12)	70.5	3.94 ddd (11, 9, 5)	70.5	3.91 m	37.9	1.93 ddd (12, 12, 12)	37.9	2.10 ddd (12, 12, 12)
2_eq_		2.03 brd (12)		-		-		2.85 brd (12)		2.76 brd (12)
3	77.1	3.96 dddd (12, 12, 6, 6)	84.9	3.82 ddd (11, 9, 5)	84.8	3.81 ddd (10, 10, 5)	67.7	3.86 dddd (11, 11, 5, 5)	68.1	3.82 dddd (12, 12, 5, 5)
4_ax_	28.7	1.45 dddd (13, 12, 12, 12)	34.1	1.42 ddd (12, 12,12)	34.0	1.41 ddd (13, 13, 10)	39.8	1.60 ddd (12, 12, 12)	43.9	2.65 dd (12, 12)
4_eq_		3.40 brd (13)		1.80 brd (12)		1.81 brd (13)		1.71 brd (11)		2.55 ddd (12, 5, 2)
5	51.1	1.22 ddd (13, 11,3)	44.8	0.99 m	44.8	1.00 m	43.0	0.99 dddd (12, 12, 3, 3)	139.6	-
6_ax_	79.8	3.62 ddd (11, 11, 5)	28.2	1.01 (o)	28.2	1.04 (o)	29.0	1.31 dddd (13, 13, 13, 4)	124.8	5.54 brd (6)
6_eq_		-		1.13 brd (11)		1.14 (o)		1.24 brd (13)		
7_ax_	41.6	1.12 ddd (13, 12, 11)	32.4	0.75 (o)	31.9	0.75 (o)	32.5	0.77 dddd (13, 12, 12, 4)	32.0	1.45 (o)
7_eq_		2.54 ddd (13, 4, 4)		1.48 (o)		1.54 brd (12)		1.53 brd (12)		1.86 brd (17)
8	34.1	1.52 dddd (12, 12, 12, 3)	34.5	1.38 dddd (12, 12, 12, 5)	33.8	1.43 dddd (11, 11, 11, 3)	36.5	1.47 (o)	33.1	1.58 ddd (11, 11, 11, 4)
9	53.9	0.51 ddd (12, 11, 4)	54.5	0.58 ddd (12, 11, 4)	53.6	0.75 (o)	54.9	0.92 ddd (12, 10, 4)	50.5	1.50 ddd (11, 11, 4)
10	36.8	-	37.0	-	37.0	-	41.6	-	42.9	-
11_ax_	21.3	1.18 (o)	21.6	1.23 dddd (14, 13, 13, 3)	32.2	1.54 (o)	23.9	1.43 ddd (13, 13, 13)	24.1	1.58 dddd (14, 12, 12, 4)
11_eq_		1.37 brdd (12, 3)		1.47 brd (14)		1.95 (o)		3.04 brdd (14, 3)		2.90 brdd (14, 4)
12_ax_	40.2	1.01 (o)	40.2	1.03 (o)	79.4	3.51 m	40.8	1.24 ddd (13, 13, 4)	40.7	1.43 ddd (12, 12, 3)
12_eq_		1.68 brdd (13, 3)		1.66 brd (12)		-		1.72 ddd (13, 3, 3)		1.74 ddd (12, 12, 3)
13	41.1	-	41.2	-	46.9	-	40.9	-	40.7	-
14	56.4	0.98 m	56.4	1.01 ddd (14, 11, 6)	55.0	1.06 ddd (14, 11, 6)	56.7	1.05 ddd (14, 11, 6)	57.0	1.14 ddd (14, 11, 6)
15_a_	32.4	1.32 ddd (13, 13, 7)	32.5	1.38 ddd (14, 12, 7)	32.2	1.56 (o)	32.8	1.42 ddd (14, 12, 6)	32.7	1.44 ddd (14, 12, 6)
15_b_		1.90 ddd (13, 7, 6)		1.99 ddd (12, 7, 6)		2.06 ddd (12, 7, 7)		2.00 ddd (12, 7, 6)		1.98 ddd (12, 7, 6)
16	81.1	4.78 ddd (9, 7, 7)	81.2	4.91 ddd (8, 7, 7)	81.3	4.99 ddd (9, 7, 7)	81.2	4.90 ddd (9, 7, 7)	81.2	4.88 ddd (9, 7, 7)
17	63.9	1.85 dd (9, 6)	64.0	1.90 dd (8, 6)	63.9	2.27 dd (9, 6)	64.1	1.92 dd (9, 6)	64.1	1.93 dd (9, 6)
18	16.9	0.80 s	16.8	0.84 s	11.5	1.12 s	17.1	0.88 s	17.0	0.93 s
19	13.5	0.70 s	13.5	0.70 s	13.5	0.73 s	8.3	1.00 s	14.9	1.24 s
20	40.8	2.17 dq (6, 7)	40.8	2.20 dq (6, 7)	41.8	2.45 dq. (7, 7)	40.9	2.20 dq. (7, 7)	40.9	2.20 dq (7, 7)
21	16.5	1.28 d (7)	16.5	1.29 d (7)	15.9	1.58 d (7)	16.4	1.26 d (7)	16.5	1.26 d (7)
22	110.6	-	110.7	-	111.0	-	110.7	-	110.7	-
23_a_	37.2	1.94 (o)	37.3	1.94 ddd (13, 13, 3)	37.4	1.99 ddd (12, 12, 3)	37.2	1.93 m	37.3	1.93 m
23_b_		2.05 (o)		2.05 ddd (13, 12, 3)		2.11 ddd (12, 12, 4)		2.04 m		2.03 m
24_a_	28.4	1.66 m	28.4	1.66 m	28.5	1.68 m	28.4	1.65 m	28.4	1.65 m
24_b_		2.04 (o)		2.03 (o)		2.05 m		2.02 m		2.03 m
25	34.5	1.92 m	34.7	1.91 m	34.6	1.91 ddq (7, 7, 6)	34.5	1.90 m	34.5	1.90 m
26_a_	75.5	3.48 dd (9, 7)	76.3	3.46 dd (9, 7)	75.6	3.44 dd (9, 7)	75.5	3.46 dd (9, 7)	75.3	3.45 dd (9, 7)
26_b_		4.08 dd (9, 6)		4.06 dd (9, 6)		4.06 dd (9, 6)		4.07 dd (9, 6)		4.06 dd (9, 6)
27	17.6	1.02 d (7)	17.6	1.01 d (7)	17.5	1.00 d (7)	17.5	0.99 d (7)	17.5	1.00 d (7)

^a^ Assignments were confirmed by ^1^H-^1^H-COSY, 1D and 2D-TOCSY, HSQC, HSQC-TOCSY and HMBC experiments. ^b^ o: overlapped with other signals.

**Table 8:** ^13^C and ^1^H NMR data (*J* in Hz) of the sugar portions of compounds **7**–**11** (pyridine-*d*_5_). ^a,b^.

	Bractofuranoside D (7)		Bractofuranoside E (8)		Bractofuranoside F (9)		Bractofuranoside G (10)		Bractofuranoside H (11)	
	*δ*_C_	*δ*_H (C-H)_	*δ*_C_	*δ*_H (C-H)_	*δ*_C_	*δ*_H (C-H)_	*δ*_C_	*δ*_H (C-H)_	*δ*_C_	*δ*_H (C-H)_
C1/C3										
		β-d-Glc		β-d-Gal		β-d-Gal		β-d-Glc		β-d-Glc
1	101.8	5.13 d (8)	103.6	4.90 d (8)	103.6	4.90 d (8)	100.6	5.04 d (8)	101.8	4.97 d (8)
2	75.5	4.06 dd (8, 9)	73.2	4.45 dd (9, 9)	73.2	4.43 dd (8, 8)	75.7	4.02 dd (9, 8)	75.5	4.03 dd (8, 8)
3	78.1	4.26 dd (9)	75.3	4.25 dd (10, 4)	75.3	4.24 dd (9, 4)	78.7	4.21 dd (9, 9)	78.7	4.22 dd (9,9)
4	71.8	4.27 dd (10, 9)	80.3	4.68 dd (4, 2)	80.3	4.67 dd (4, 2)	72.4	4.14 dd (9, 9)	72.5	4.14 dd (9, 9)
5	78.2	3.86 m	76.1	4.13 dd (9, 9)	76.1	4.11 dd (7, 7)	78.6	3.93 ddd (9, 6, 3)	78.3	3.94 ddd (9, 6, 3)
6	62.8	4.43 dd (12, 3)	61.0	4.64 (o)	60.9	4.62 (o)	63.6	4.58 dd (12, 3)	63.8	4.56 dd (12, 3)
		4.32 dd (12, 5)		4.26 (o)		4.26 (o)		4.35 ddd (12, 6)		4.34 dd (12, 6)
				β-d-Glc		β-d-Glc				
1′			107.3	5.27 d (8)	107.3	5.26 d (8)				
2′			75.8	4.14 dd (8, 9)	75.8	4.13 dd (8, 9)				
3′			78.9	4.22 dd (9, 9)	78.7	4.22 (o)				
4′			72.4	4.07 dd (10, 9)	72.4	4.06 dd (9, 9)				
5′			78.6	4.00 ddd (10, 7, 3)	78.6	3.99 ddd(10, 7, 3)				
6′			63.2	4.58 dd (12, 3)	63.2	4.57 dd (12, 3)				
				4.22 dd (12,5)		4.20 dd (12, 5)				
C6		β-d-Glc								
	106.3	4.89 d (8)								
	75.8	4.03 dd (8, 8)								
	78.6	4.21 dd (9, 9)								
	71.9	4.25 dd (9, 8)								
	78.7	3.91 m								
	63.0	4.48 dd (12, 3)								
		4.37 dd (12, 5)								
**C26**										
		β-d-Glc		β-d-Glc		β-d-Glc		β-d-Glc		β-d-Glc
1	105.3	4.80 d (8)	105.3	4.80 d (8)	105.3	4.80 d (8)	105.3	4.79 d (8)	105.3	4.79 d (8)
2	75.1	4.01 dd (8, 8)	75.3	4.01 dd (9, 8)	75.3	4.00 dd (9, 8)	75.5	4.00 dd (8, 8)	75.5	4.00 dd (8, 8)
3	78.4	4.21 (o)	78.3	4.22 (o)	78.7	4.22 (o)	78.7	4.22 (o)	78.7	4.22 (o)
4	71.5	4.22 (o)	72.0	4.21 (o)	71.8	4.21 (o)	71.8	4.23 (o)	71.8	4.23 (o)
5	78.3	3.92 ddd (8, 6, 3)	78.6	3.92 ddd (10, 7, 3)	78.6	3.92 ddd (8, 6, 3)	78.5	3.92 ddd (8, 6, 3)	78.6	3.92 ddd (8, 6, 3)
6	62.9	4.53 dd (12, 3)	62.9	4.53 dd (12, 3)	62.9	4.53 dd (12, 3)	62.9	4.53 dd (12, 3)	62.9	4.53 dd (12, 3)
		4.38 dd (12, 5)		4.38 dd (12, 5)		4.37 dd (12, 5)		4.38 dd (12, 5)		4.38 dd (12, 5)

^a^ Assignments were confirmed by ^1^H-^1^H-COSY, 1D and 2D-TOCSY, HSQC, HSQC-TOCSY and HMBC experiments. ^b^ o: overlapped with other signals.

## Data Availability

Data are contained within the article and Supplementary Materials.

## Supplementary Materials

The following supporting information can be downloaded at: https://www.mdpi.com/article/10.3390/plants13182570/s1. Tables S1, S2 and Figures S1 and S2: HMAI tools, Figures S3–S7: Selected areas of 2D-NMR spectra of saponin-rich fraction (SF) of *Agave bracteosa* for dereplication. Figures S8–S37: HRESI MS^E^, ^1^H and ^13^C NMR spectra of isolated compounds. Figures S38–S41: TOCSY and NOESY spectra of Bractofuranoside G (10) as an example.
