# Supplementary material for: Dereplication of New Saponins from Agave bracteosa
Source: Plants (Basel). 2024 Sep 13;13(18):2570. doi: 10.3390/plants13182570 (PMC11435350; doi:10.3390/plants13182570)
Supplement: Supplementary file 1 [file plants-13-02570-s001.zip › plants-3160681-supplementary.pdf]

Supporting information

Dereplication of new saponins from *Agave bracteosa*

Francesca Guzzo[1,2], Alexandra G. Durán[1], Laura L. Rostoll[1], Francisco A. Macías[1] and Ana M. Simonet[1]*

[1] Allelopathy Group, Department of Organic Chemistry, Institute of Biomolecules (INBIO), School of Science, University of Cádiz, 11510 Puerto Real, Cádiz, Spain; francesca.guzzo@uca.es (F.G.); alexandra.garcia@uca.es (A.G.D.); laura.lopezros@alum.uca.es (L.L.R.); famacias@uca.es (F.A.M.)

[2] Department of Environmental Biological and Pharmaceutical Sciences and Technologies-DiSTABiF,
University of Campania "Luigi Vanvitelli", Via Vivaldi 43, 81100 Caserta, Italy

* Correspondence: ana.simonet@uca.es

Table of Contents

Table S1. HMAI Table for doublets……………………………………………………………………4

Table S2. HMAI Table for singlets……………………………………………………………………5

Figure S1. HMAI flowchart for doublets ……………………………..……………………………6

Figure S2. HMAI flowchart for singlets ……………………………………………………………7

Figure S3. HMBC of saponin-enriched fraction (SP) of *Agave bracteosa.* Selected area of methyl signals for aglycone (I). (600 MHz, Pyridine-d_5)…………………………………………………………8

Figure S4. HMBC of saponin-enriched fraction (SP) of *Agave bracteosa.* Selected area of methyl signals for aglycone (II). (600 MHz, Pyridine-d_5)……………………………………………………...9

Figure S5. HSQC-TOCSY of saponin-enriched fraction (SP) of *Agave bracteosa.* Selected area of sugar chain signals. (600 MHz, Pyridine-d_5). 10

Figure S6. HSQC of saponin-enriched fraction (SP) of *Agave bracteosa.* Selected area of sugar chain signals. (600 MHz, Pyridine-d_5). 11

Figure S7. HMBC of saponin-enriched fraction of *Agave bracteosa.* Selected area of sugar chain signals. (600 MHz, Pyridine-d_5).. 12

Figure S8. HRESI MS^E (negative mode) of (25*S*)-Cantalasaponin-1 (1) 13

Figure S9. ^{1}H NMR spectrum of (25*S*)-Cantalasaponin-1 (1) (700 MHz, Pyridine-d_5) 14

Figure S10. ^{13}C NMR spectrum of (25*S*)-Cantalasaponin-1 (700 MHz, Pyridine-d_5)........................ 16

Figure S11. HRESI MS^E (negative mode) of Bractofuranoside A (2)... 17

Figure S12. ^{1}H NMR spectrum of Bractofuranoside A (2) (700 MHz, Pyridine-d_5).......................... 18

Figure S13. ^{13}C NMR spectrum of Bractofuranoside A (2) (700 MHz, Pyridine-d_5)......................... 19

Figure S14. HRESI MS^E (negative mode) of Tribufuroside D (3)... 20

Figure S15. ^{1}H NMR spectrum of Tribufuroside D (700 MHz, Pyridine-d_5) 21

Figure S16. ^{13}C NMR spectrum of Tribufuroside D (700 MHz, Pyridine-d_5) 22

Figure S17. HRESI MS^E (negative mode) of Bractofuranoside B (5) 23

Figure S18. ^{1}H NMR spectrum of Bractofuranoside B (5) (700 MHz, Pyridine-d_5) 24

Figure S19. ^{13}C NMR spectrum of Bractofuranoside B (5) (700 MHz, Pyridine-d_5)......................... 25

Figure S20. HRESI MS^E (negative mode) of Bractofuranoside C (6)... 26

Figure S21. ^{1}H NMR spectrum of Bractofuranoside C (6) (700 MHz, Pyridine-d_5).......................... 27

Figure S22. ^{13}C NMR spectrum of Bractofuranoside C (6) (700 MHz, Pyridine-d_5)......................... 28

Figure S23. HRESI MS^E (negative mode) of Bractofuranoside D (7) 29

Figure S24. ^{1}H NMR spectrum of Bractofuranoside D (7) (700 MHz, Pyridine-d_5).......................... 30

Figure S25. ^{13}C NMR spectrum of Bractofuranoside D (7) (700 MHz, Pyridine-d_5)......................... 31

Figure S26. HRESI MS^E (negative mode) of Bractofuranoside E (8) 32

Figure S27. ^{1}H NMR spectrum of Bractofuranoside E (8) (700 MHz, Pyridine-d_5) 33

Figure S28. ^{13}C NMR spectrum of Bractofuranoside E (8) (700 MHz, Pyridine-d_5) 34

Figure S29. HRESI MS^E (negative mode) of Bractofuranoside F (9) 35

Figure S30. ^{1}H NMR spectrum of Bractofuranoside F (9) (700 MHz, Pyridine-d_5).......................... 36

Figure S31. ^{13}C NMR spectrum of Bractofuranoside F (9) (700 MHz, Pyridine-d_5) 37

Figure S32. HRESI MS^{E} (negative mode) of Bractofuranoside G (10) **38**

Figure S33. ^{1}H NMR spectrum of Bractofuranoside G (10) (700 MHz, Pyridine-*d_5*) **39**

Figure S34. ^{13}C NMR spectrum of Bractofuranoside G (10) (700 MHz, Pyridine-*d_5*) **40**

Figure S35. HRESI MS^{E} (negative mode) of Bractofuranoside H (11) **41**

Figure S36. ^{1}H NMR spectrum of Bractofuranoside H (11) (700 MHz, Pyridine-*d_5*) **42**

Figure S37. ^{13}C NMR spectrum of Bractofuranoside H (11) (700 MHz, Pyridine-*d_5*) **43**

Figure S38. Example of 1D TOCSY spectra (120 ms): Sugar chain of Bractofuranoside G (10). (700 MHz, Pyridine-*d_5*)) **44**

Figure S39. Example of 1D TOCSY spectra (120 ms): Aglycone of Bractofuranoside G (10). (700 MHz, Pyridine-*d_5*) **45**

Figure S40. Example of 1D NOESY spectra (300 ms): Sugar chain of Bractofuranoside G (10). (700 MHz, Pyridine-*d_5*) **46**

Figure S41. Example of 2D NOESY spectra (300 ms): Aglycone of Bractofuranoside G (10). (700 MHz, Pyridine-*d_5*) **47**

Table S1. HMAI table for doublets. Reprinted with permission from Simonet *et al.*, *Phytochem. Anal.*, 2021: *32*, 38-61 (DOI: 10.1002/pca.2946). Copyright 2021 John Wiley & Sons. Order Number: 5856460076616

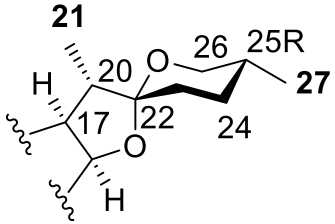

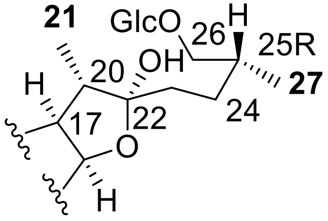

Structural Features						HMBC signals							
C-9	C-12	C-23	C-24	C-22	C-25	**H-27**	C-24	C-25	C-26	**H-21**	C-17	C-20	C-22
				SP	R	0.67	29.1	30.6	66.7	1.12	62.9	41.9	109.3
				SP	DB	-	-	-	-	1.08	63.2	41.9	109.4
				F	R	0.96	28.5	34.4	75.3	1.30	63.9	40.8	110.7
				FM	R	0.98	28.2	34.2	75.2	1.16	64.1	40.5	112.7
		OHα		SP	R	0.72	38.9	31.8	66.0	1.16	62.6	35.9	111.7
			OGlcβ	SP	R*	1.12	81.5	38.2	65.1	1.02	62.3	42.1	111.6
		OHα	OGlcβ	SP	R*	1.19	87.9	37.9	64.1	1.15	62.0	34.6	112.7
	CO			SP	S	1.05	26.2	27.5	65.2	1.35	54.2	43.1	109.8
	CO			SP	R	0.64	29.2	30.5	66.9	1.31	54.3	42.6	109.3
	CO			F	R	0.96	28.4	34.3	75.3	1.53	54.9	41.3	110.9
	CO			F	S	1.01	28.3	34.4	75.3	1.51	54.8	41.3	110.8
DB	CO			SP	R	0.67	29.2	30.5	67.0	1.38	54.5	43.0	109.5
	OHβ			SP	R	0.67	29.4	30.7	66.9	1.41	63.0	43.1	109.6

OH: hydroxyl; DB: double bond; CO: carbonyl; SP: spirostanic; F: furostanic; R/S/α/β: chiral center configuration.

* R is the relative configuration; S is the absolute configuration because a glucopyranosyloxy moiety is at C-24.

Table S2. HMAI table for singlets. Reprinted with permission from Simonet *et al.*, *Phytochem. Anal.*, 2021: *32*, 38-61 (DOI: 10.1002/pca.2946). Copyright 2021 John Wiley & Sons. Order Number: 5856460076616

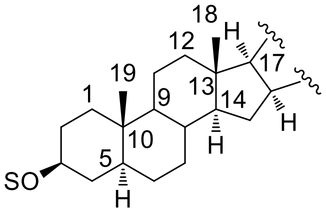

Structural Features								HMBC signals									
C-2	C-5	C-6	C-9	C-12	C-22	C-23	C-24	**H-18**	C-12	C-13	C-14	C-17	**H-19**	C-1	C-5	C-9	C-10
	α				SP			0.80	40.0	40.5	56.1	62.9	0.62	36.9	44.4	54.1	35.6
	α				SP	OH*α*	OGlc*β*	1.01	40.7	41.4	56.6	62	0.75	37.5	45.6	54.6	35.9
	α			CO	SP			1.03	212.8	55.4	55.9	54.3	0.61	36.6	44.4	55.5	36.3
	α		DB	CO	SP			0.98	204.3	51.3	52.7	54.5	0.79	35.0	42.5	171.3	39.5
OH *α*	α		DB	CO	SP			0.97	204.3	51.4	52.7	54.6	0.86	43.5	42.5	170.5	40.6
	α			OH*β*	SP			1.06	79.3	46.6	55.2	63.0	0.64	37.2	44.7	53.6	35.9
OH *α*	α				SP			0.78	40.0	40.6	56.3	63.0	0.69	45.6	44.6	54.3	36.8
	β				SP			0.79	40.3	40.9	56.5	63.1	0.84	30.8	36.9	40.2	35.2
OH *β*	β				SP			0.77	40.2	40.8	56.3	63.1	0.87	40.5	36.4	41.4	36.9
	DB				SP			0.80	39.9	40.5	56.7	62.9	0.85	37.5	141.1	50.3	37.1
	DB			CO	F			1.13	212.9	55.4	56.0	54.9	0.91	37.0	140.9	52.4	37.6
OH *α*	DB				SP			0.78	39.8	40.5	56.5	62.9	0.91	45.8	140.1	50.2	38.0
OH *α*	DB				SP		OGlc*β*	0.71	39.7	40.4	56.5	62.3	0.91	45.7	140.1	50.1	37.9
OH *α*	DB				F			0.85	39.9	40.8	56.5	63.9	0.92	45.8	140.1	50.3	38.0
OH *α*	DB				FM			0.77	39.6	40.8	56.4	64.1	0.91	45.7	140.1	50.2	37.9
	α	OH*α*			SP			0.81	40.0	40.7	56.2	62.9	0.68	37.6	52.1	54.0	36.4
	α	OGlc*α*			SP			0.76	40.0	40.8	56.4	63.0	0.68	37.5	50.9	53.8	36.7
	α	OGlc*α*			SP	OH*α*		0.96	22.7	41.4	56.5	62.6	0.74	37.8	51.3	54.0	36.8

OH: hydroxyl; DB: double bond; CO: carbonyl; SP: spirostanic; F: furostanic; *α*/*β*: chiral center configuration.

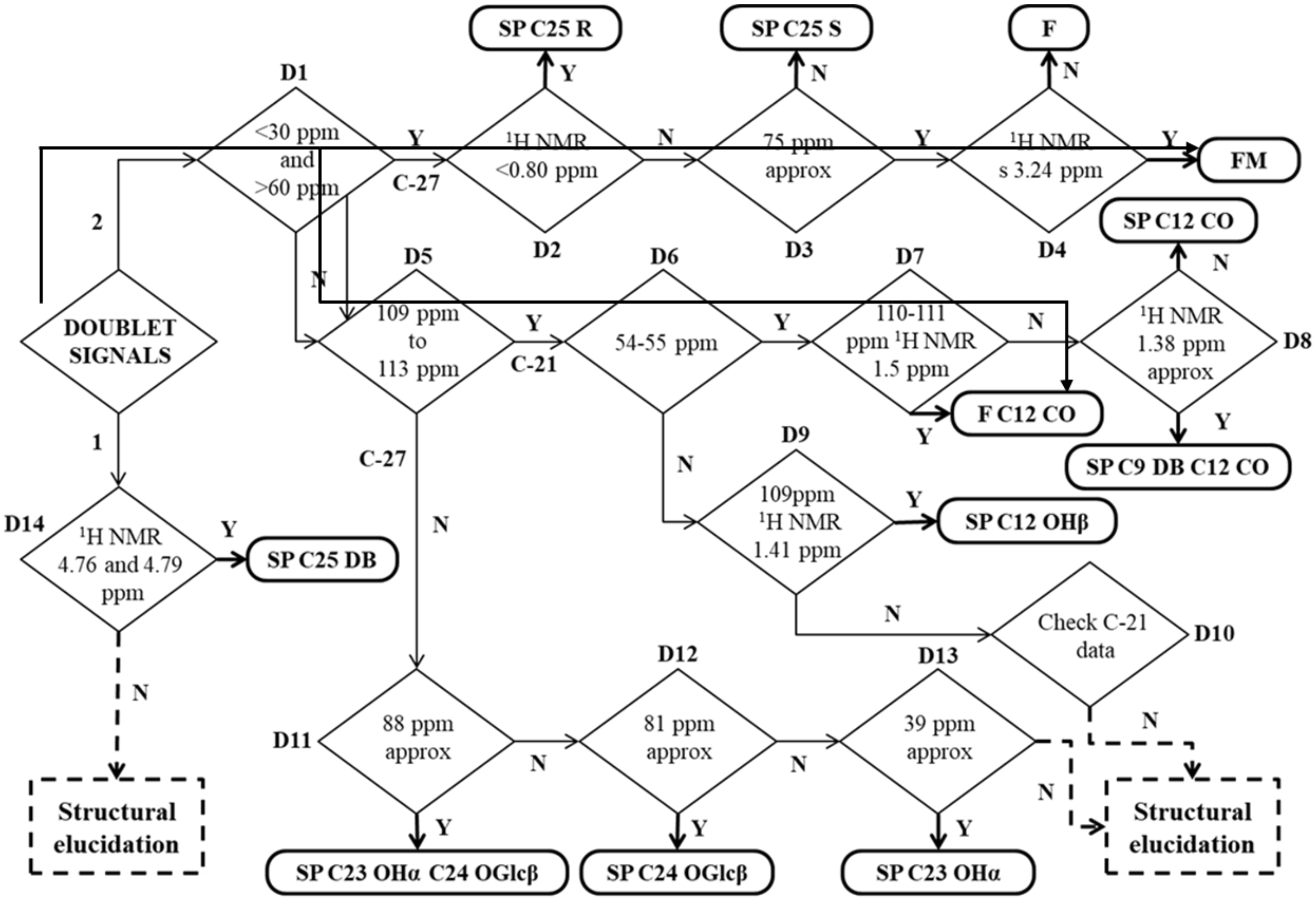

SP: spirostanic; F furostanic; FM: methoxyfurostanic; R/S/α/β quiral configuration; CO: carbonyl group; OH: hydroxyl group; DB: double bound; Oglc: glucopyranosyloxy; Y: Yes; N: Not.

Figure S1. HMAI Flowchart of doublets. v 2024. Example of flowchart informations for furostanic saponins. Adapted with permission from Simonet *et al.*, Phytochem. Anal., 2021: *32*, 38-61 (DOI: 10.1002/pca.2946). Copyright 2021 John Wiley & Sons. Order Number: 5856460076616

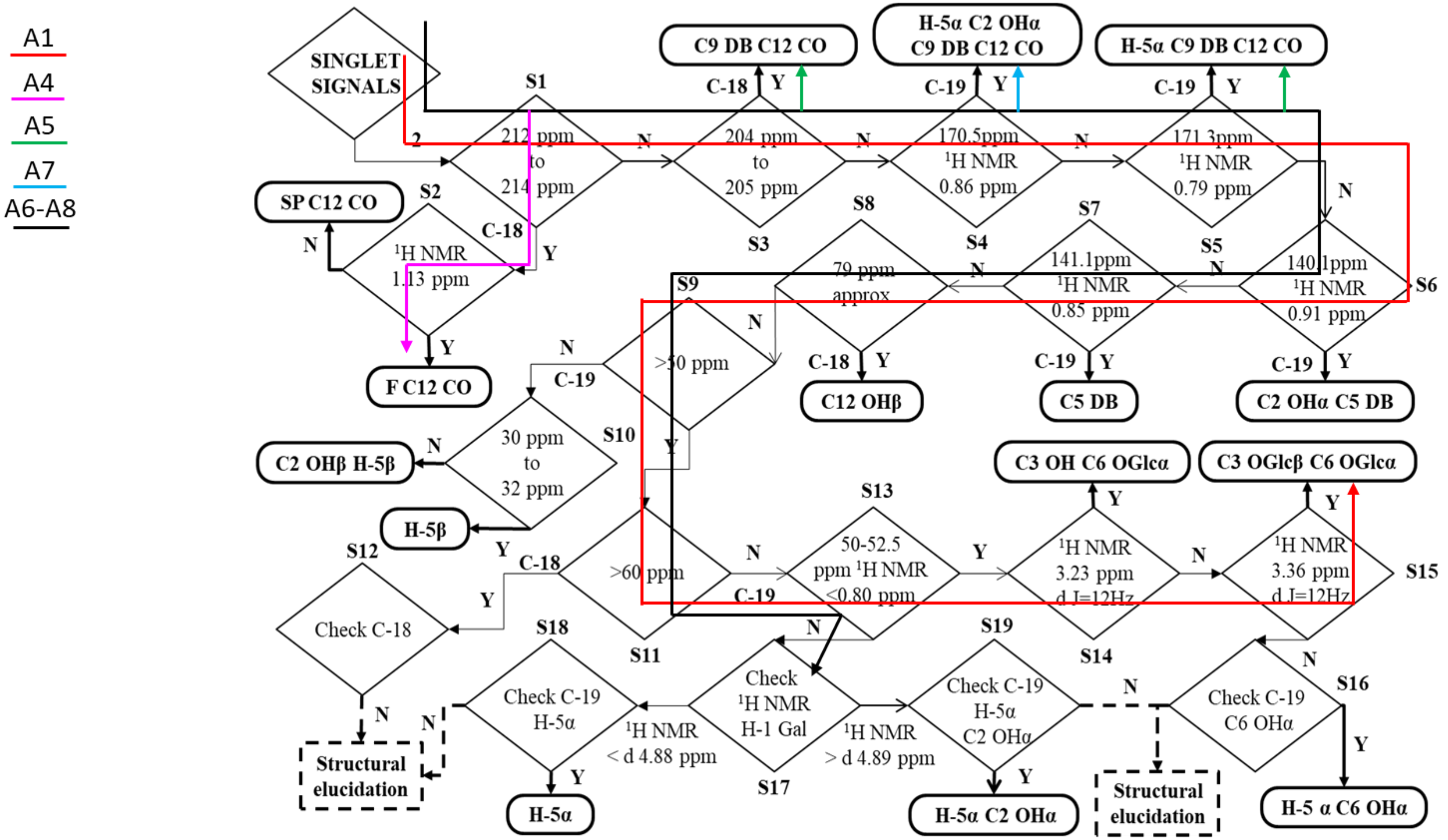

SP: spirostanic; F furostanic; R/S/α/β quiral configuration; CO: carbonyl group; OH: hydroxyl group; DB: double bound; Oglc: glucopyranosyloxy; Y: Yes; N: Not.

Figure S2. HMAI Flowchart of singlets. v 2024. Example of flowchart information to dereplicate A1, A5-A8. Adapted with permission from Simonet *et al*., Phytochem. Anal., 2021: *32*, 38-61 (DOI: 10.1002/pca.2946). Copyright 2021 John Wiley & Sons. Order Number: 5856460076616.

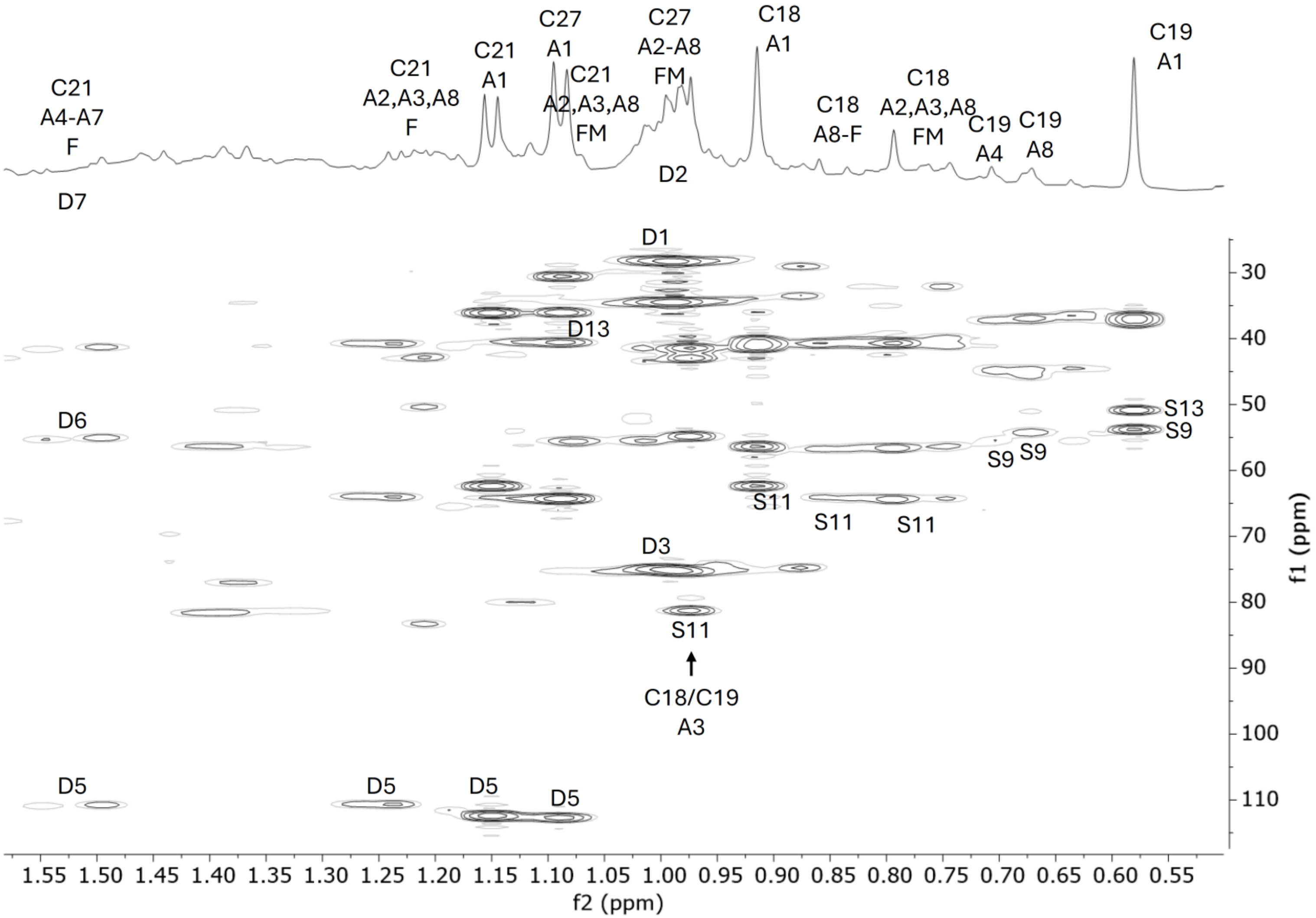

Figure S3. HMBC of saponin-enriched fraction (SP) of *Agave bracteosa.* Selected area of methyl signals for aglycone (I). (600 MHz, Pyridine-d_5).

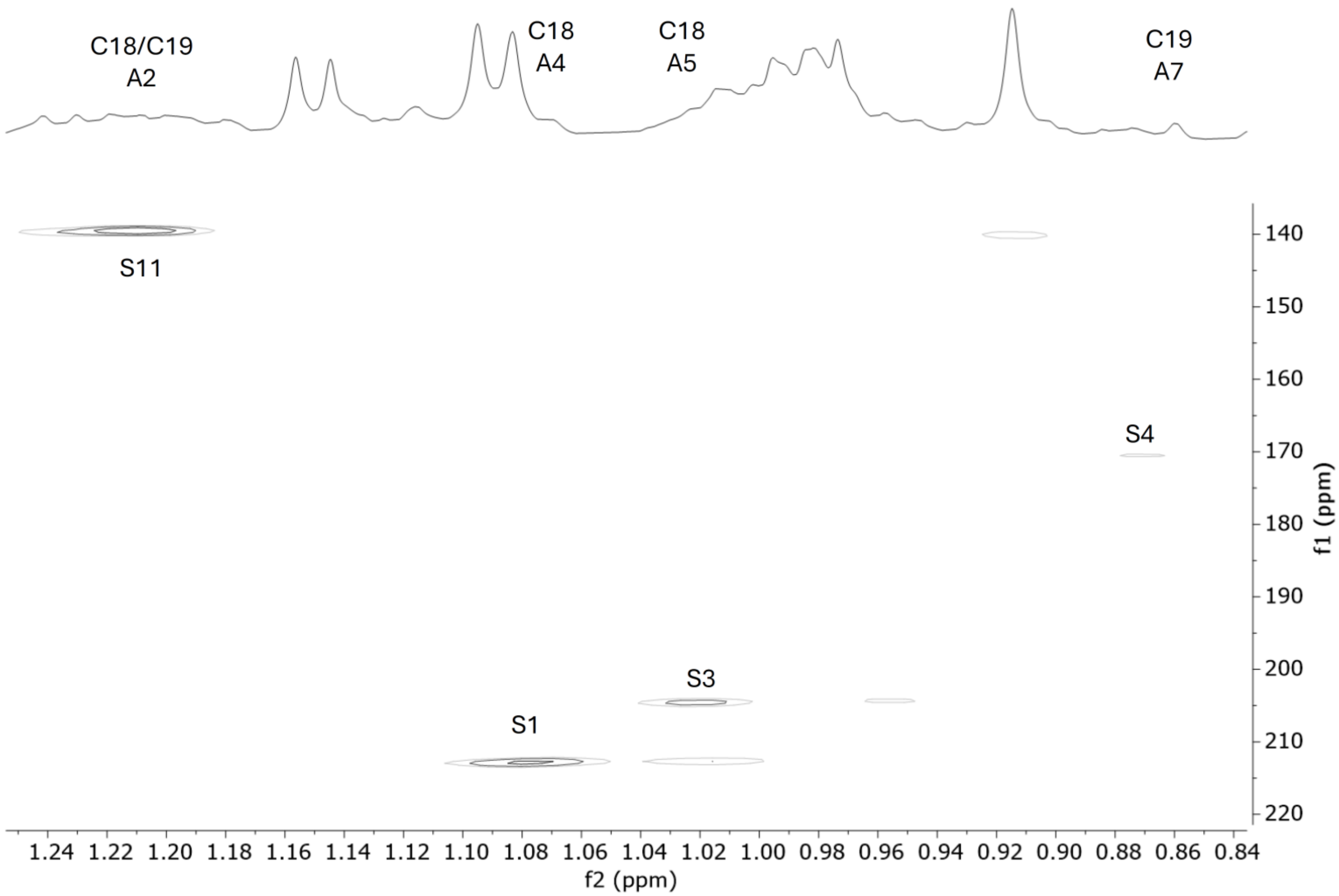

Figure S4. HMBC of saponin-enriched fraction (SP) of *Agave bracteosa.* Selected area of methyl signals for aglycone (II). (600 MHz, Pyridine-d_5).

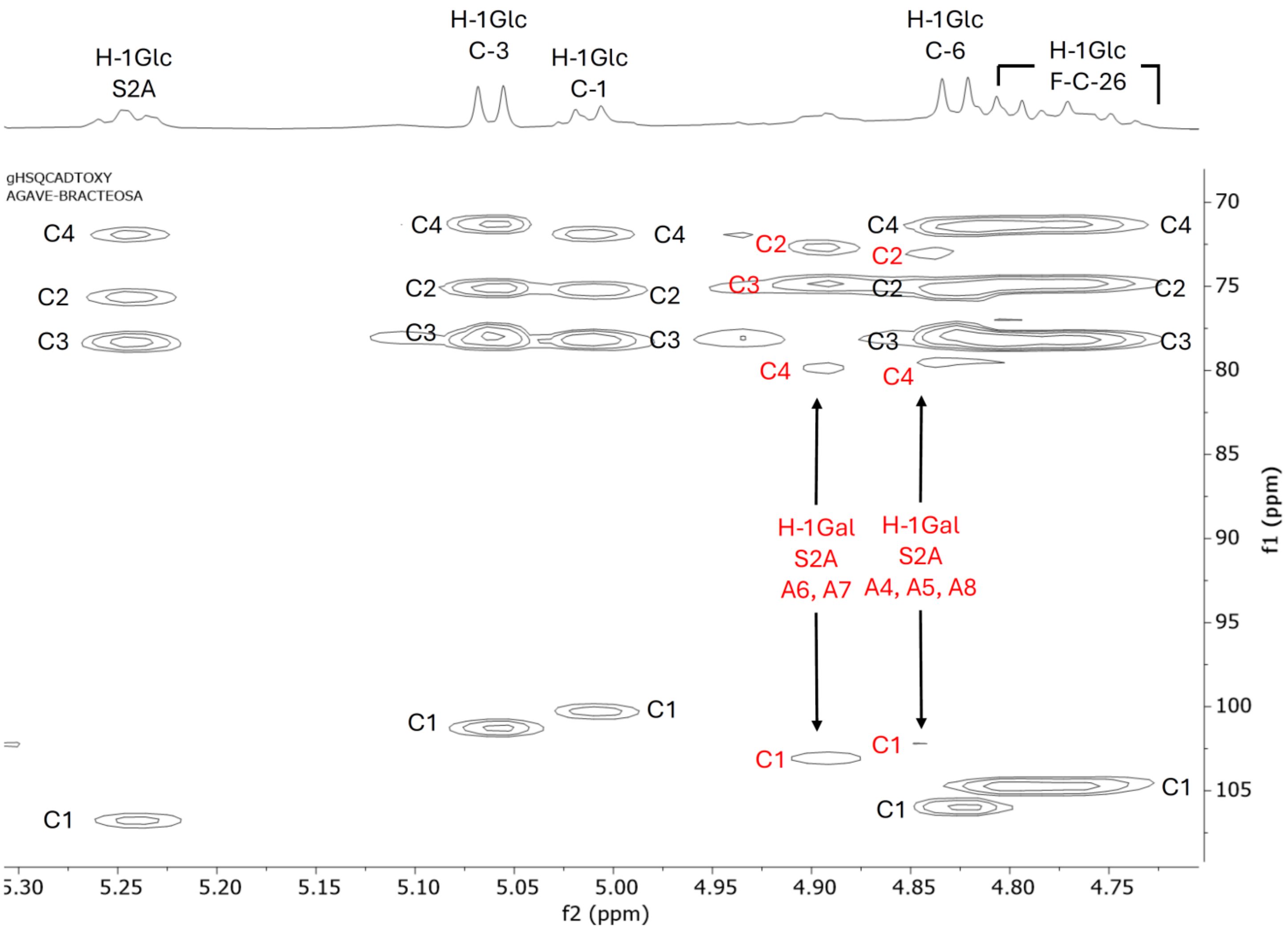

Figure S5. HSQC-TOCSY of saponin-enriched fraction (SP) of *Agave bracteosa.* Selected area of sugar chain signals. (600 MHz, Pyridine-d_5).

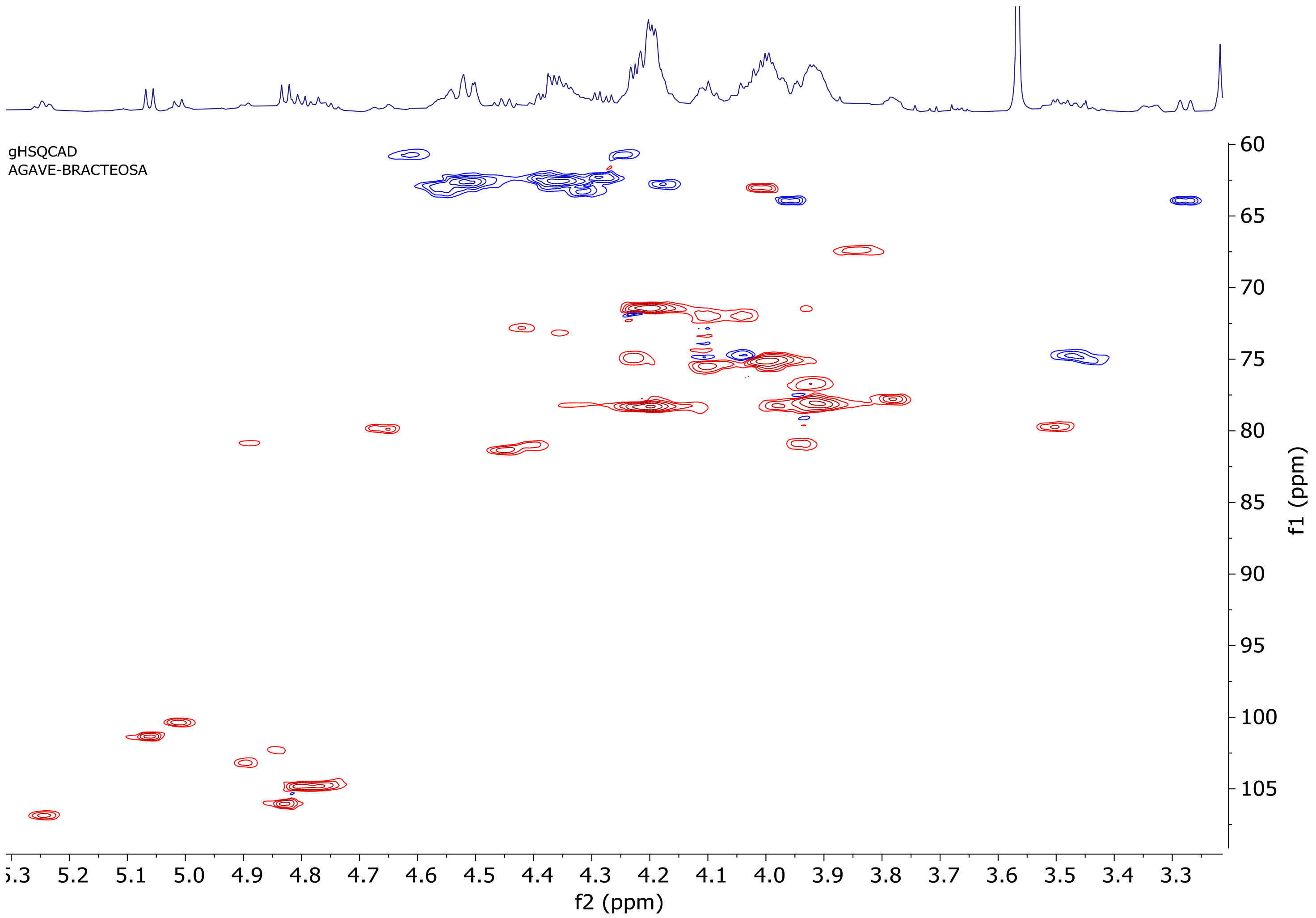

Figure S6. HSQC of saponin-enriched fraction (SP) of *Agave bracteosa*. Selected area of sugar chain signals. (600 MHz, Pyridine-d_5).

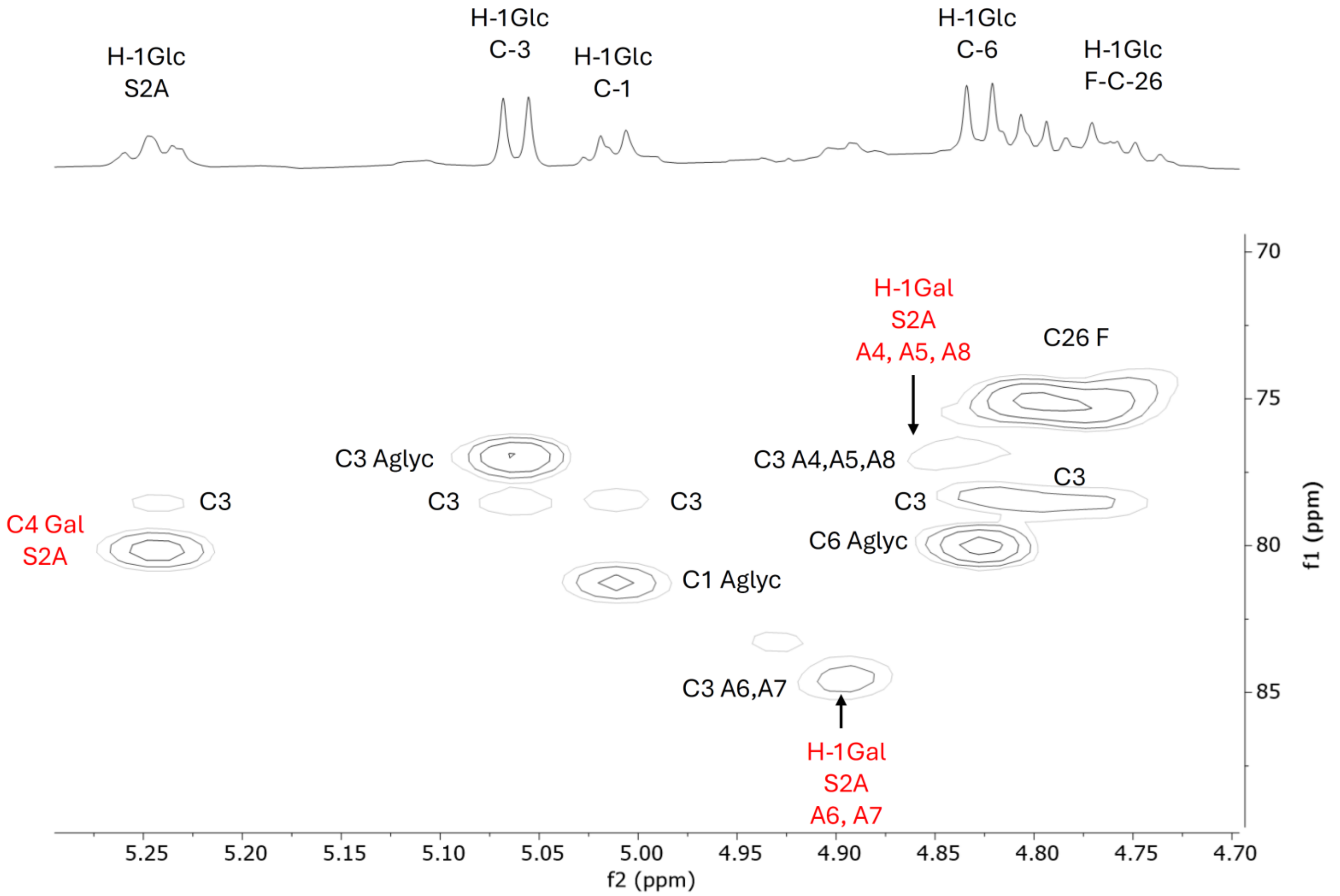

Figure S7. HMBC of saponin-enriched fraction of *Agave bracteosa.* Selected area of sugar chain signals. (600 MHz, Pyridine-d_5).

Elemental Composition Report

Tolerance = 5.0 mDa / DBE: min = -1.5, max = 50.0
Element prediction: Off
Number of isotope peaks used for i-FIT = 3

Monoisotopic Mass, Even Electron Ions
260 formula(e) evaluated with 3 results within limits (up to 50 closest results for each mass)
Elements Used:
C: 0-60 H: 0-100 O: 0-50

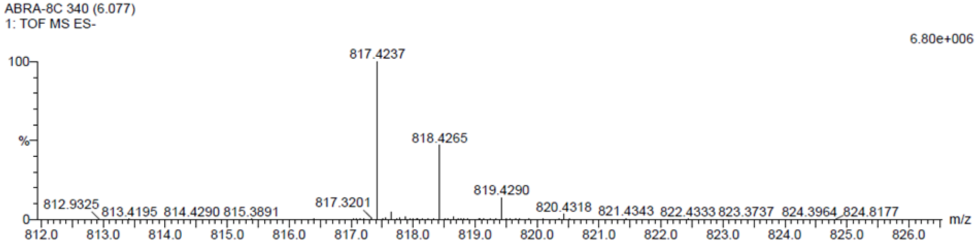

Minimum:	80.00				-1.5				
Maximum:	100.00		5.0	10.0	50.0				

Mass	RA	Calc. Mass	mDa	PPM	DBE	i-FIT	Norm	Conf(%)	Formula
817.4237	100.00	817.4222	1.5	1.8	8.5	712.7	0.000	99.97	C40 H65 O17
		817.4257	-2.0	-2.4	30.5	722.0	9.385	0.01	C58 H57 O4
		817.4280	-4.3	-5.3	-0.5	721.3	8.652	0.02	C33 H69 O22

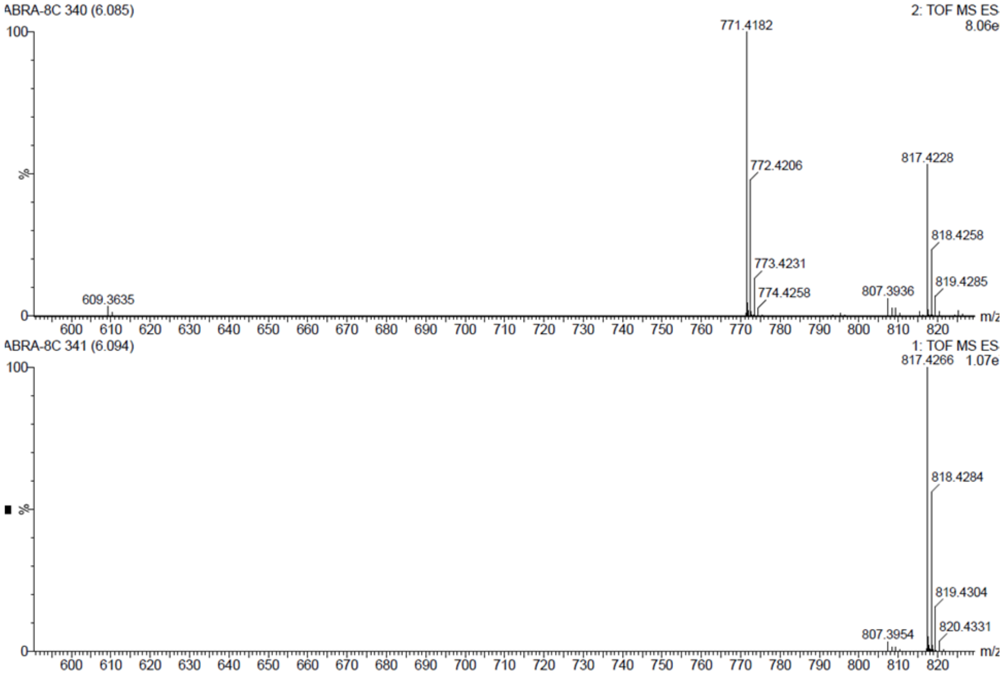

Figure S8. Elemental composition calculated for $C_{40}H_{65}O_{17}$ [M + CH_3COO^-]$^-$ and HRESI MS^E (negative mode) of (25*S*)-Cantalasaponin-1 (1).

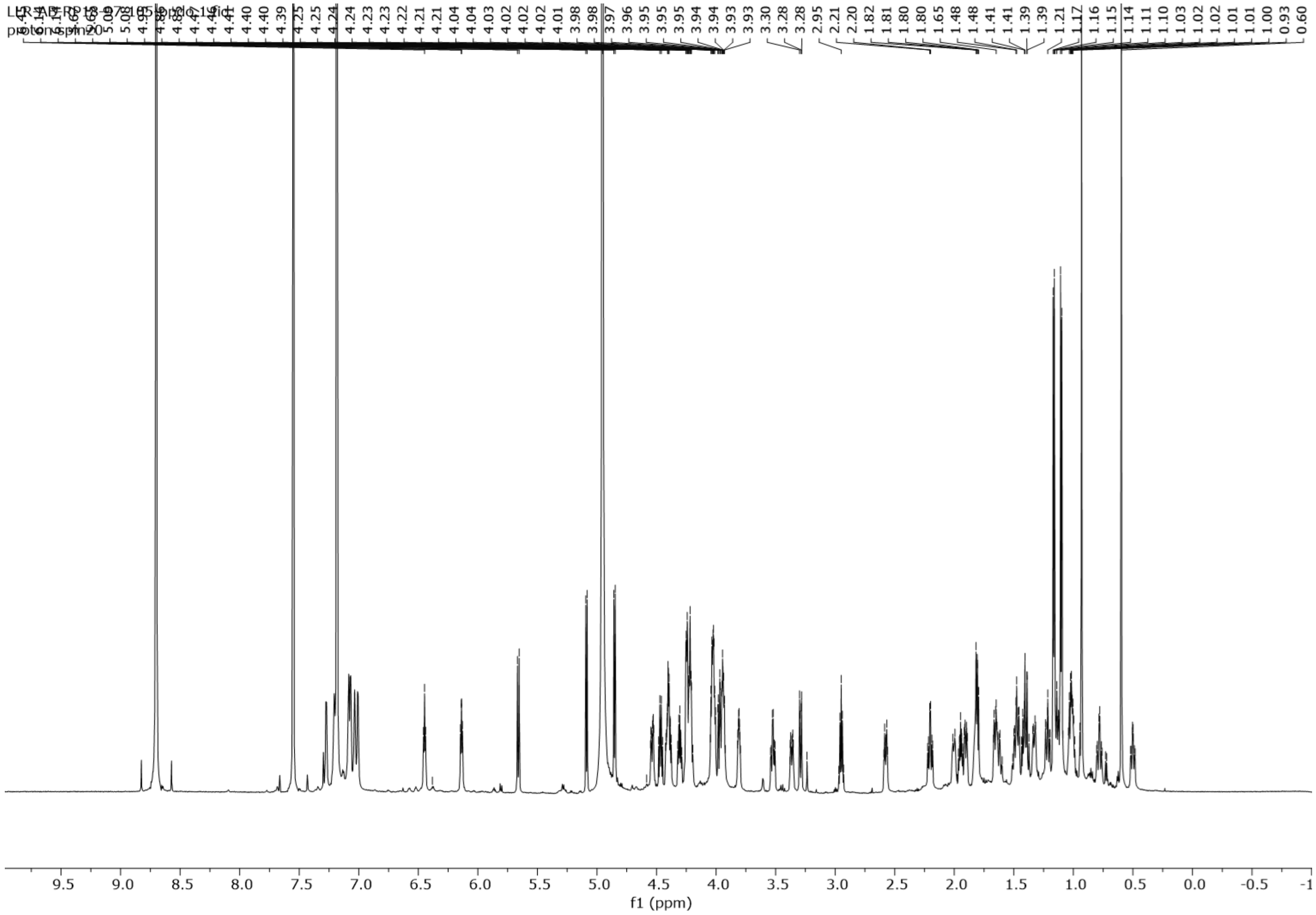

Figure S9. ^{13}H NMR spectrum of (25*S*)-Cantalasaponin-1 (1) (700 MHz, Pyridine-*d*$_5$)

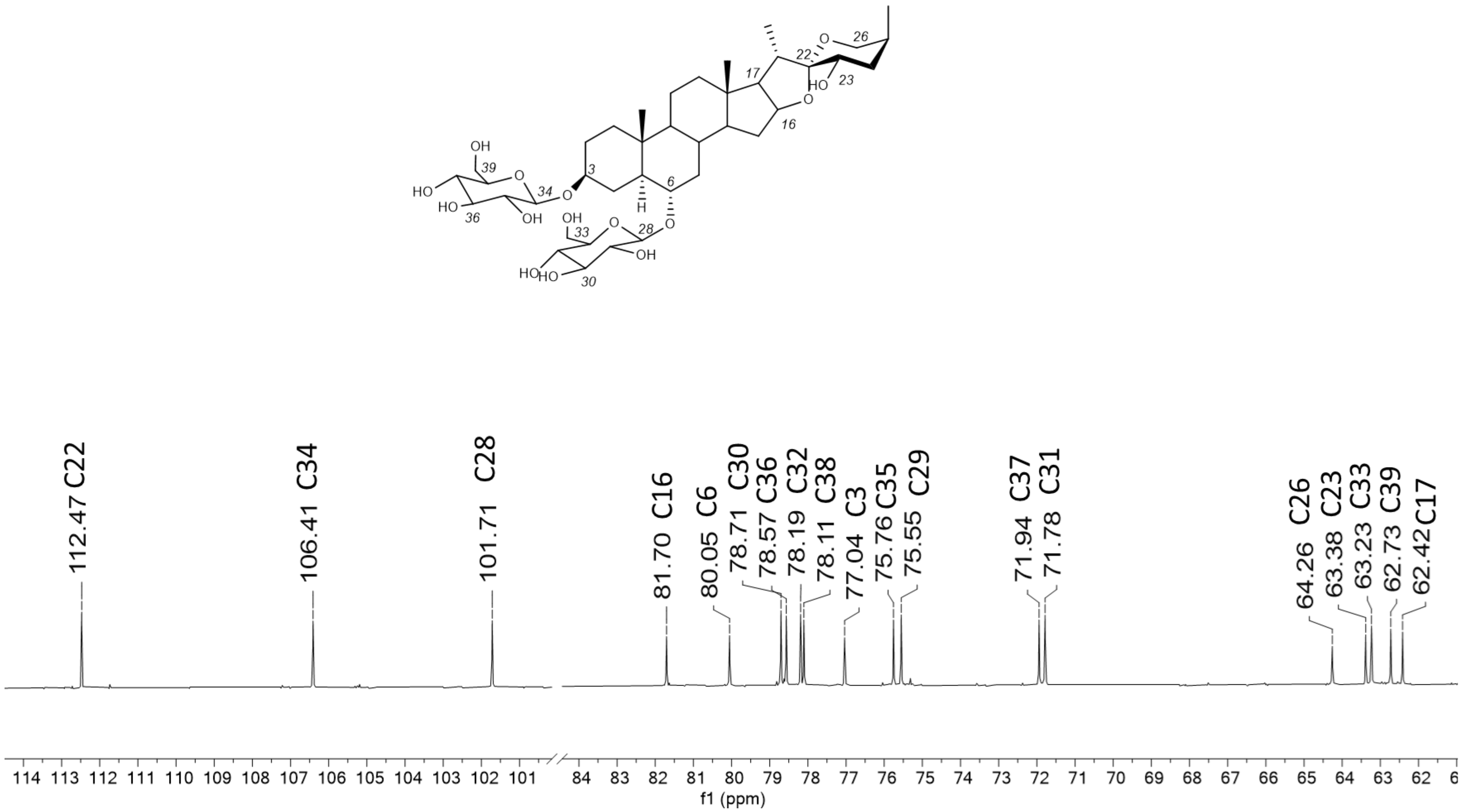

Figure S10. ^{13}C NMR spectrum of (25*S*)-Cantalasaponin-1 (1) (700 MHz, Pyridine-*d*$_5$)

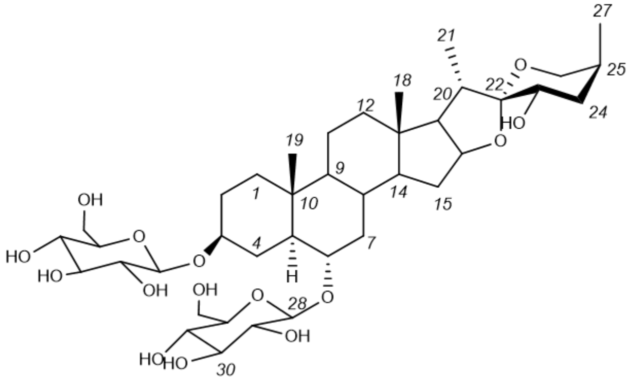

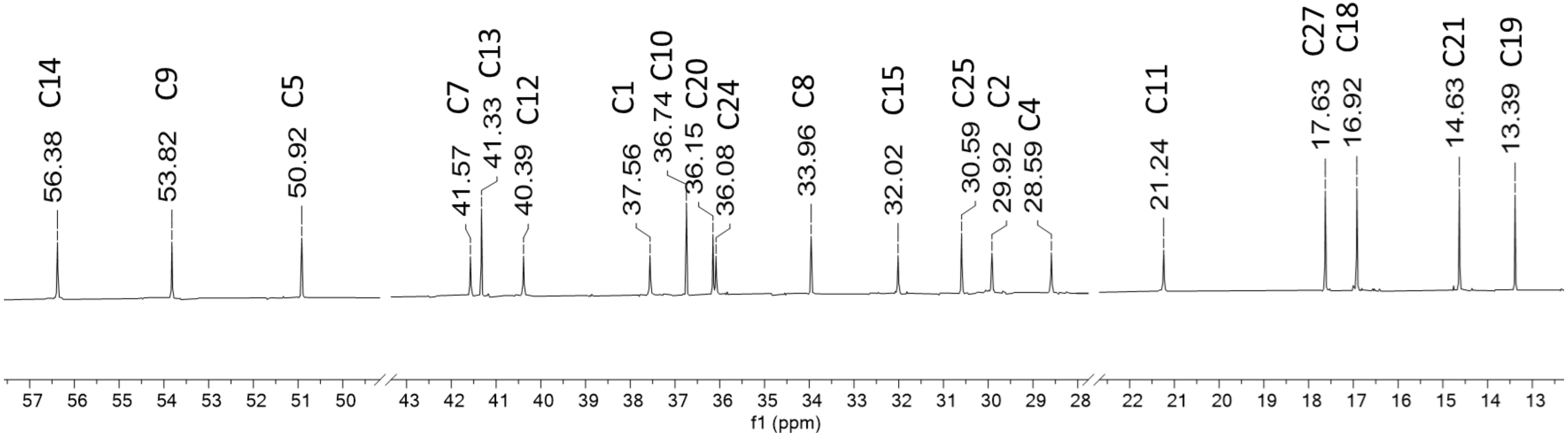

Figure S10bis. ^{13}**C NMR spectrum of (25*S*)-Cantalasaponin-1 (1) (700 MHz, Pyridine-*d*$_5$)**

Tolerance = 5.0 mDa / DBE: min = -1.5, max = 50.0
Element prediction: Off
Number of isotope peaks used for i-FIT = 3

Monoisotopic Mass, Even Electron Ions
246 formula(e) evaluated with 1 results within limits (up to 50 closest results for each mass)
Elements Used:
C: 0-60 H: 0-100 O: 0-50

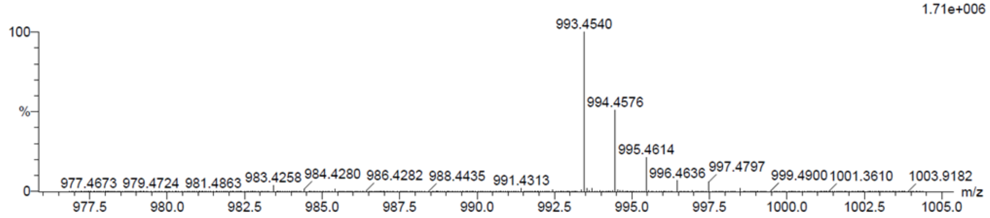

Minimum:	80.00				-1.5				
Maximum:	100.00		5.0	10.0	50.0				
Mass	RA	Calc. Mass	mDa	PPM	DBE	i-FIT	Norm	Conf(%)	Formula
993.4540	100.00	993.4543	-0.3	-0.3	10.5	568.3	n/a	n/a	C46 H73 O23

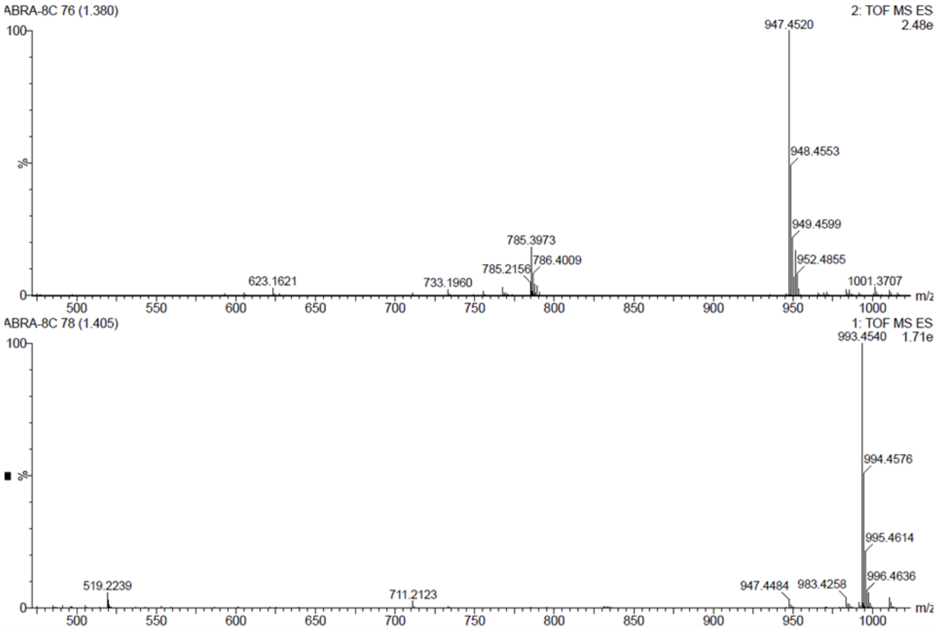

Figure S11. Elemental composition calculated for $C_{46}H_{73}O_{23}$ [M + CH_3COO^-]$^-$ and HRESI MS^E (negative mode) of Bractofuranoside A (2).

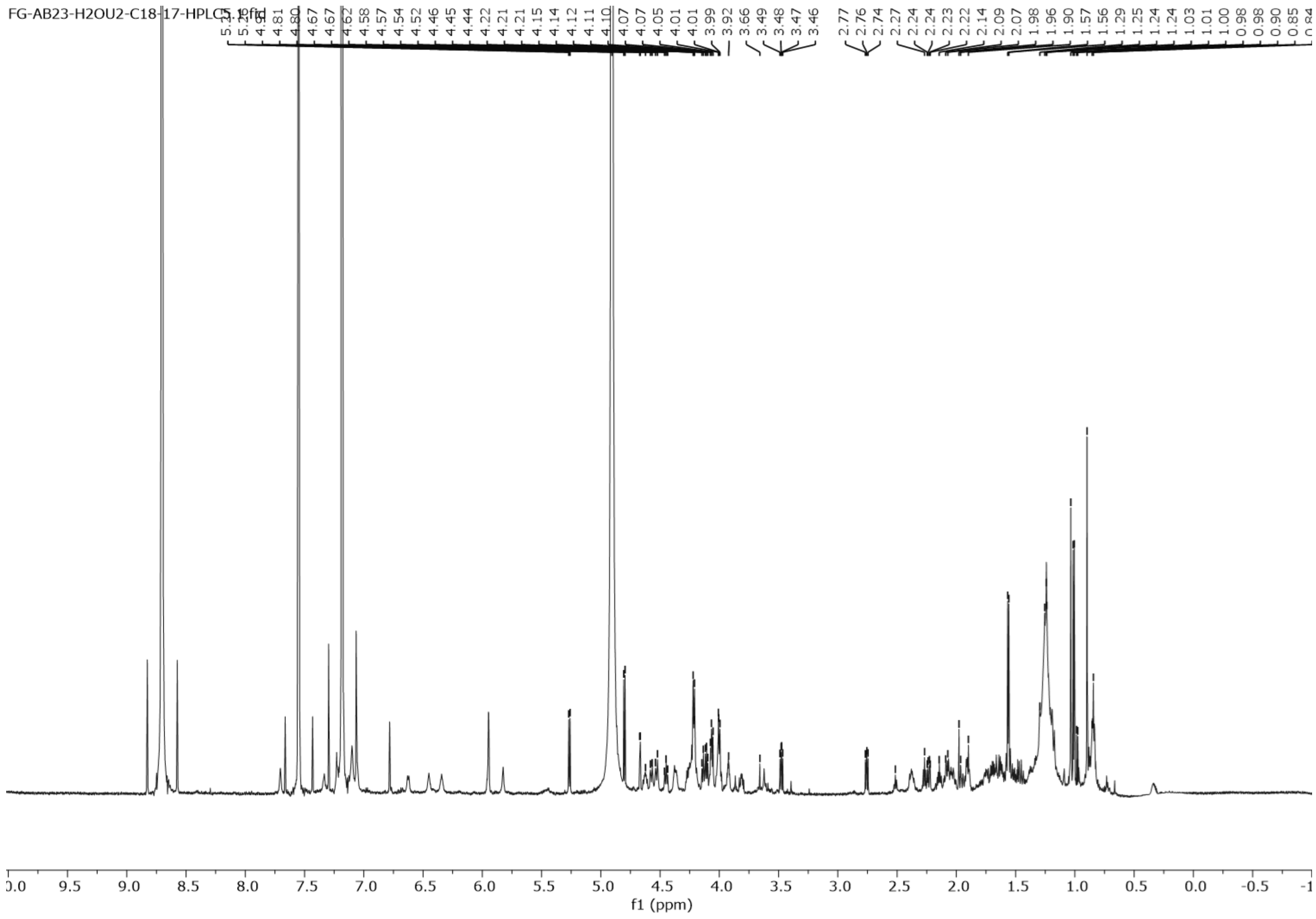

Figure S12. ^{1}H NMR spectrum of Bractofuranoside A (2) (700 MHz, Pyridine-d_5)

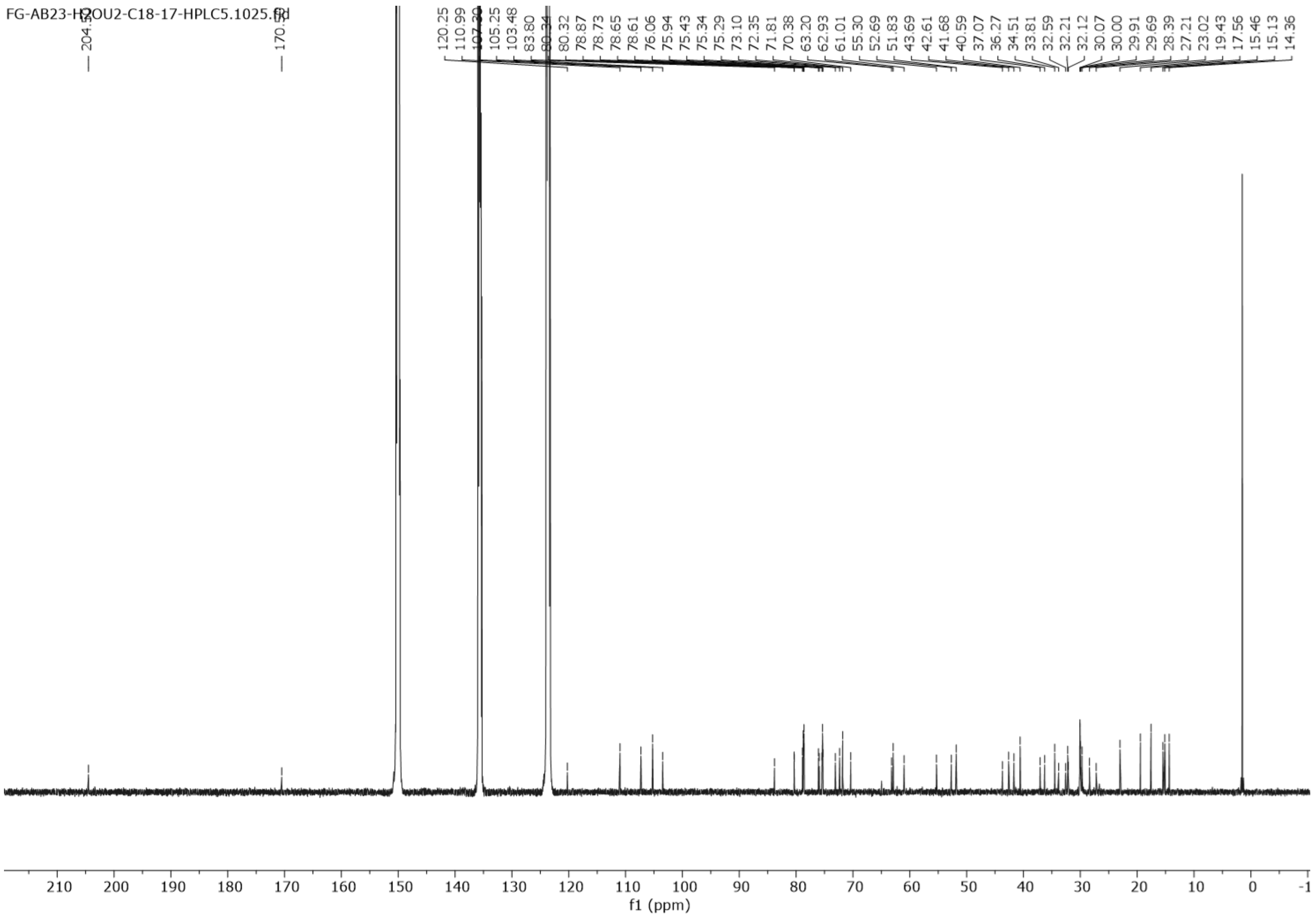

Figure S13. ^{13}C NMR spectrum of Bractofuranoside A (2) (700 MHz, Pyridine-d_5)

Elemental Composition Report **Page 1**

Tolerance = 5.0 mDa / DBE: min = -1.5, max = 50.0
Element prediction: Off
Number of isotope peaks used for i-FIT = 3

Monoisotopic Mass, Even Electron Ions
253 formula(e) evaluated with 2 results within limits (up to 50 closest results for each mass)
Elements Used:
C: 0-60 H: 0-100 O: 0-50
ABRA-8C 84 (1.508)
1: TOF MS ES-

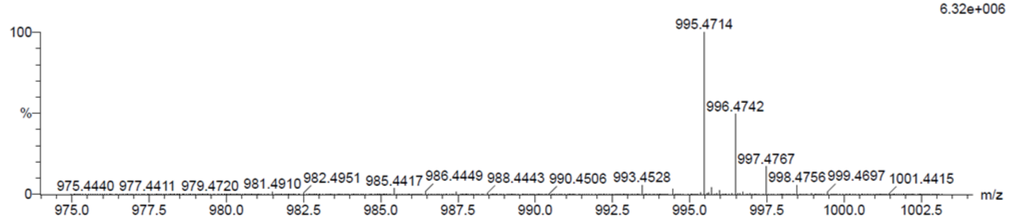

Minimum:	80.00				-1.5				
Maximum:	100.00		5.0	10.0	50.0				
Mass	RA	Calc. Mass	mDa	PPM	DBE	i-FIT	Norm	Conf(%)	Formula
995.4714	100.00	995.4699	1.5	1.5	9.5	584.4	0.001	99.89	C46 H75 O23
		995.4758	-4.4	-4.4	0.5	591.2	6.795	0.11	C39 H79 O28

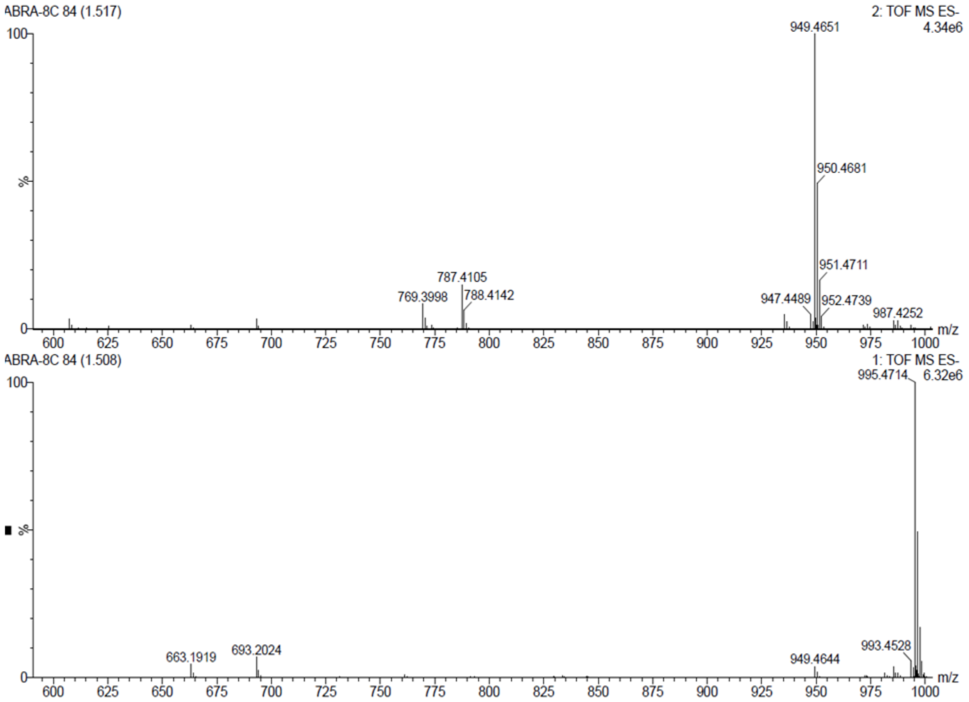

Figure S14. Elemental composition calculated for $C_{46}H_{75}O_{23}$ [M + CH_3COO^-]$^-$ and HRESI MS^E (negative mode) of Tribufuroside D (3).

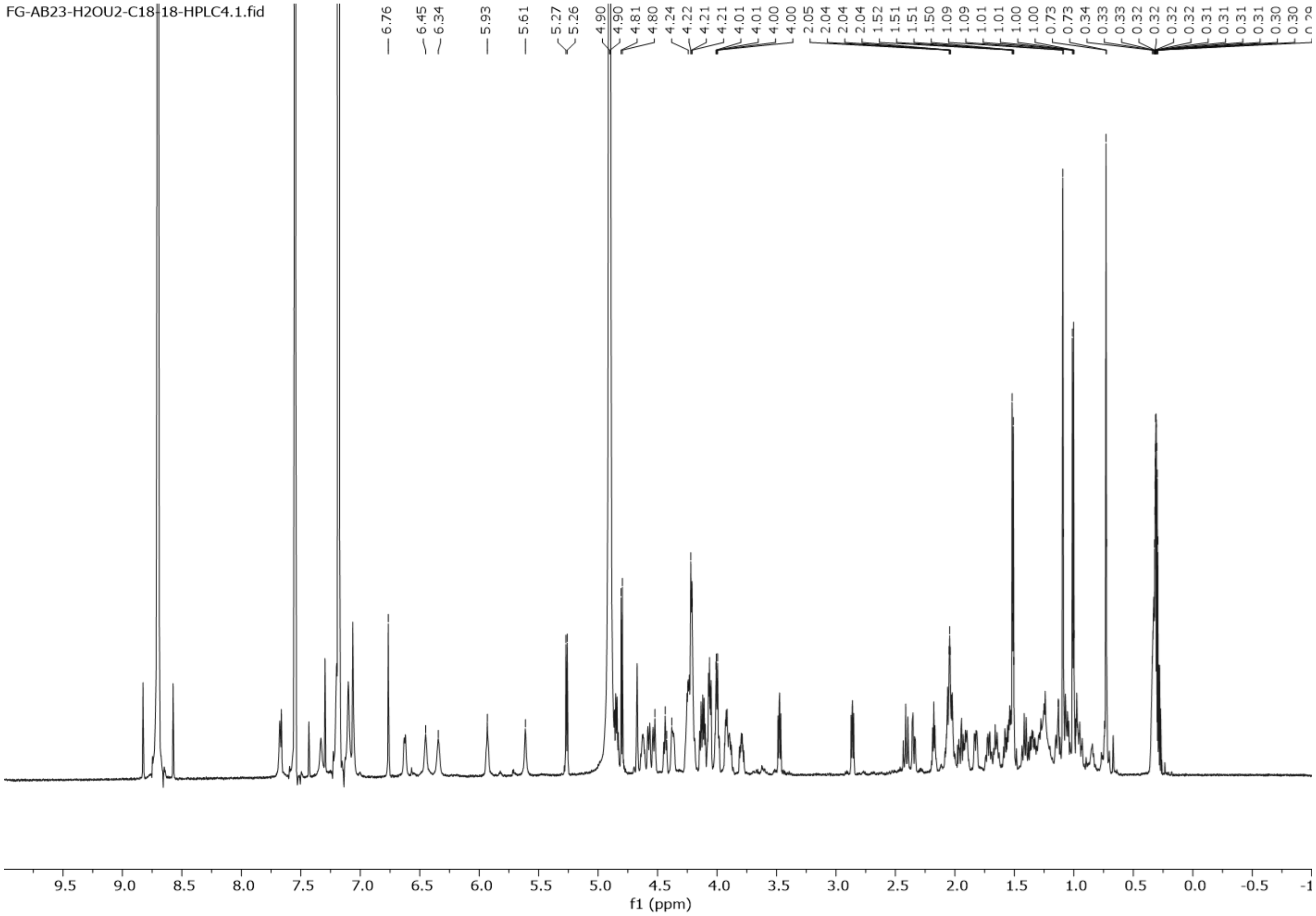

Figure S15. ^{1}H NMR spectrum of Tribuforoside D (3) (700 MHz, Pyridine-d_5)

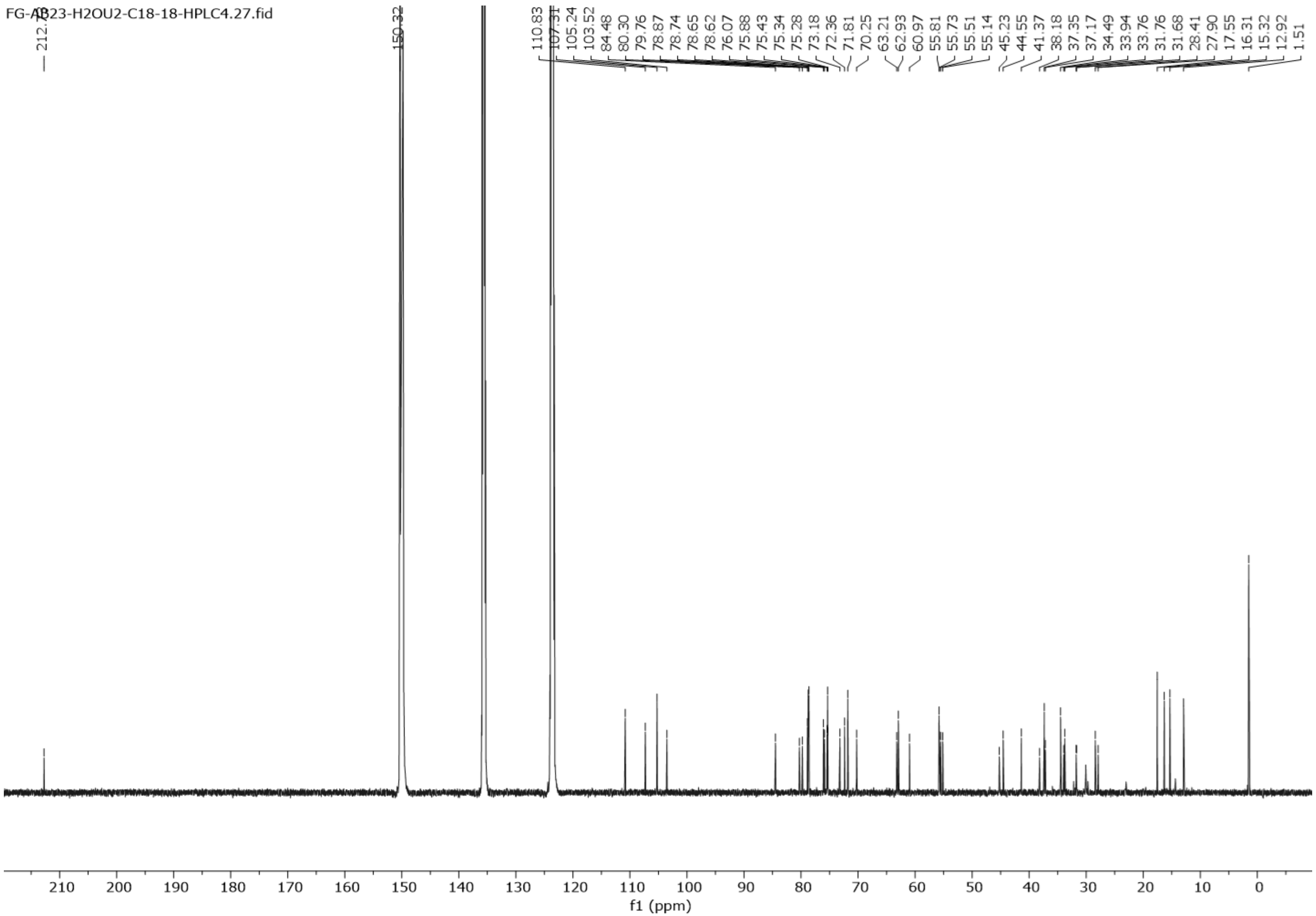

Figure S16. ^{13}C **NMR spectrum of Tribuforoside D (3) (700 MHz, Pyridine-*d*$_5$)**

Elemental Composition Report

Tolerance = 5.0 mDa / DBE: min = -1.5, max = 50.0
Element prediction: Off
Number of isotope peaks used for i-FIT = 3

Monoisotopic Mass, Even Electron Ions
250 formula(e) evaluated with 1 results within limits (up to 50 closest results for each mass)
Elements Used:
C: 0-60 H: 0-100 O: 0-50
ABRA-8C 278 (4.974)
1: TOF MS ES-

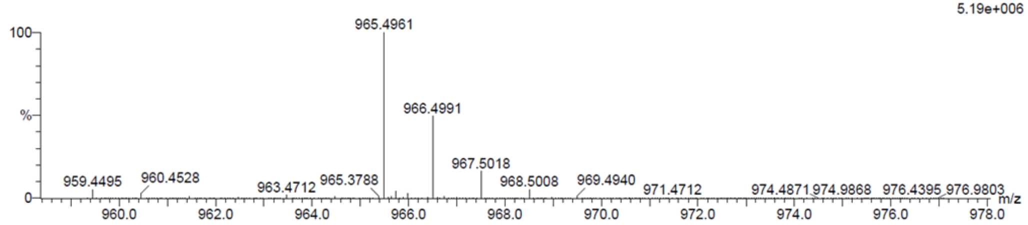

Minimum:	80.00				-1.5				
Maximum:	100.00		5.0	10.0	50.0				

Mass	RA	Calc. Mass	mDa	PPM	DBE	i-FIT	Norm	Conf(%)	Formula
965.4961	100.00	965.4957	0.4	0.4	8.5	627.1	n/a	n/a	C46 H77 O21

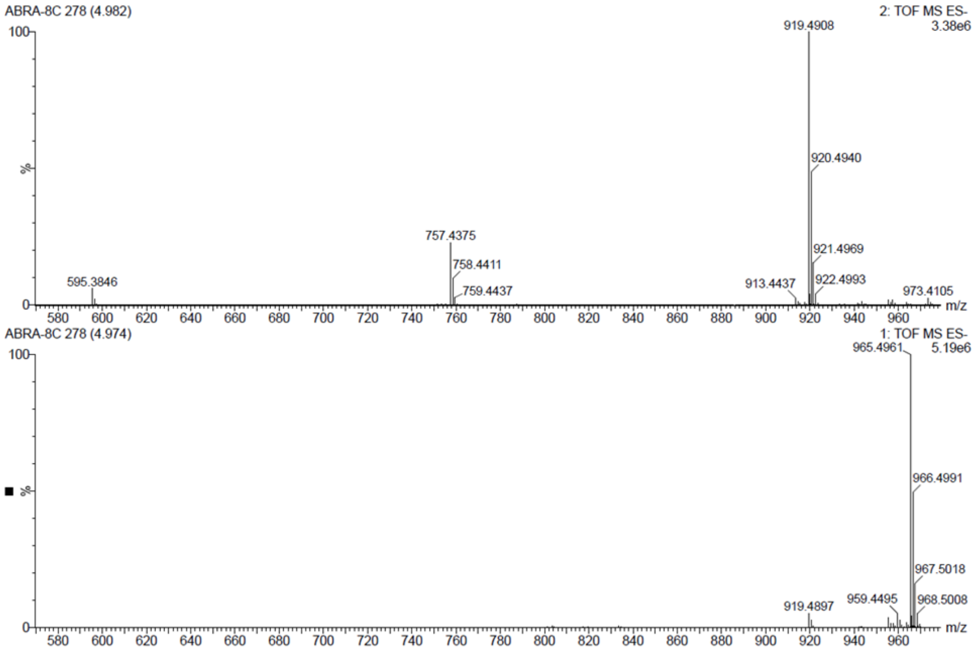

Figure S17. Elemental composition calculated for $C_{46}H_{77}O_{21}$ [M + CH_3COO^-]$^-$ and HRESI MS^E (negative mode) of Bractofuranoside B (5).

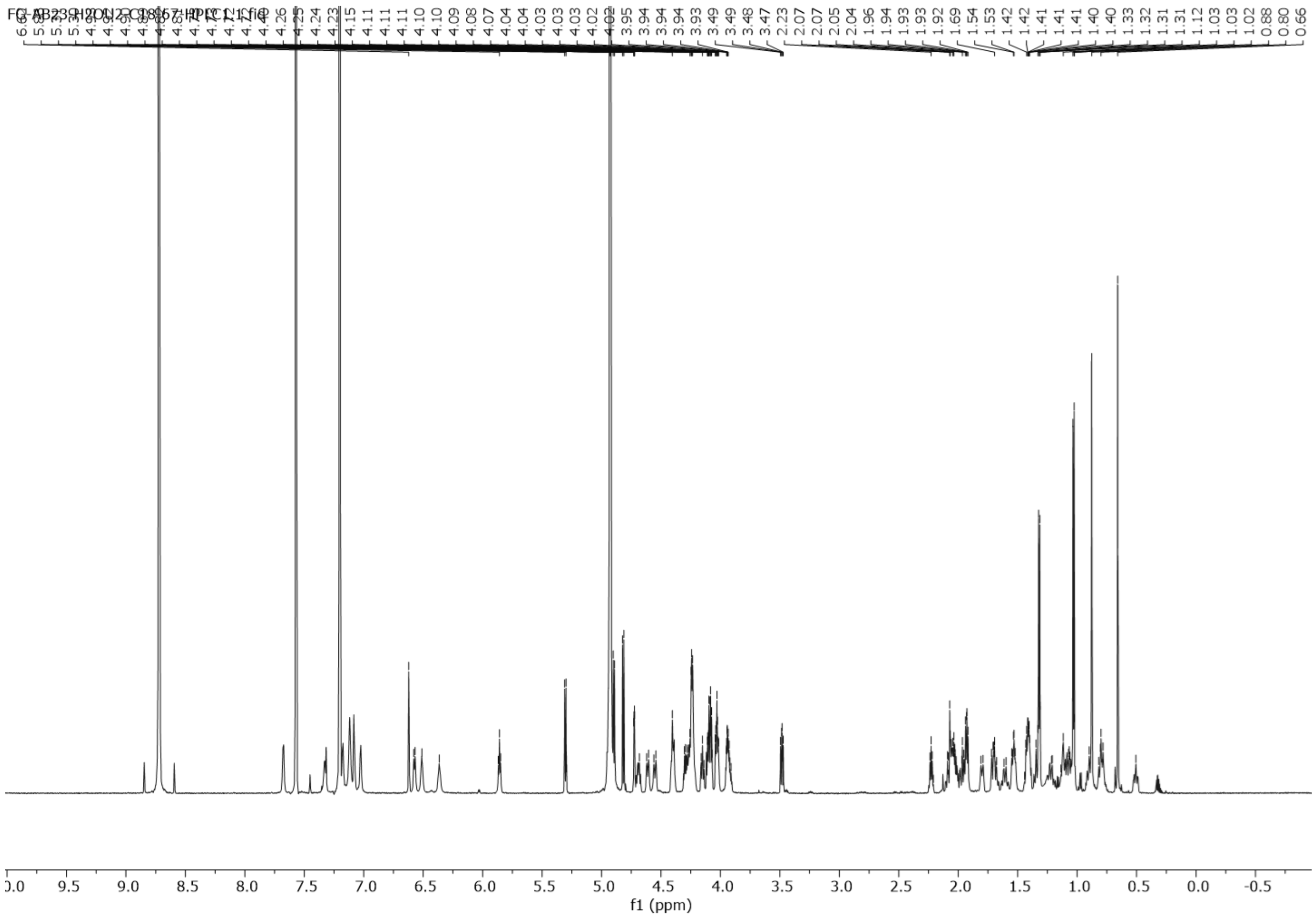

Figure S18. ^{1}H NMR spectrum of Bractofuranoside B (5) (700 MHz, Pyridine-*d_5*)

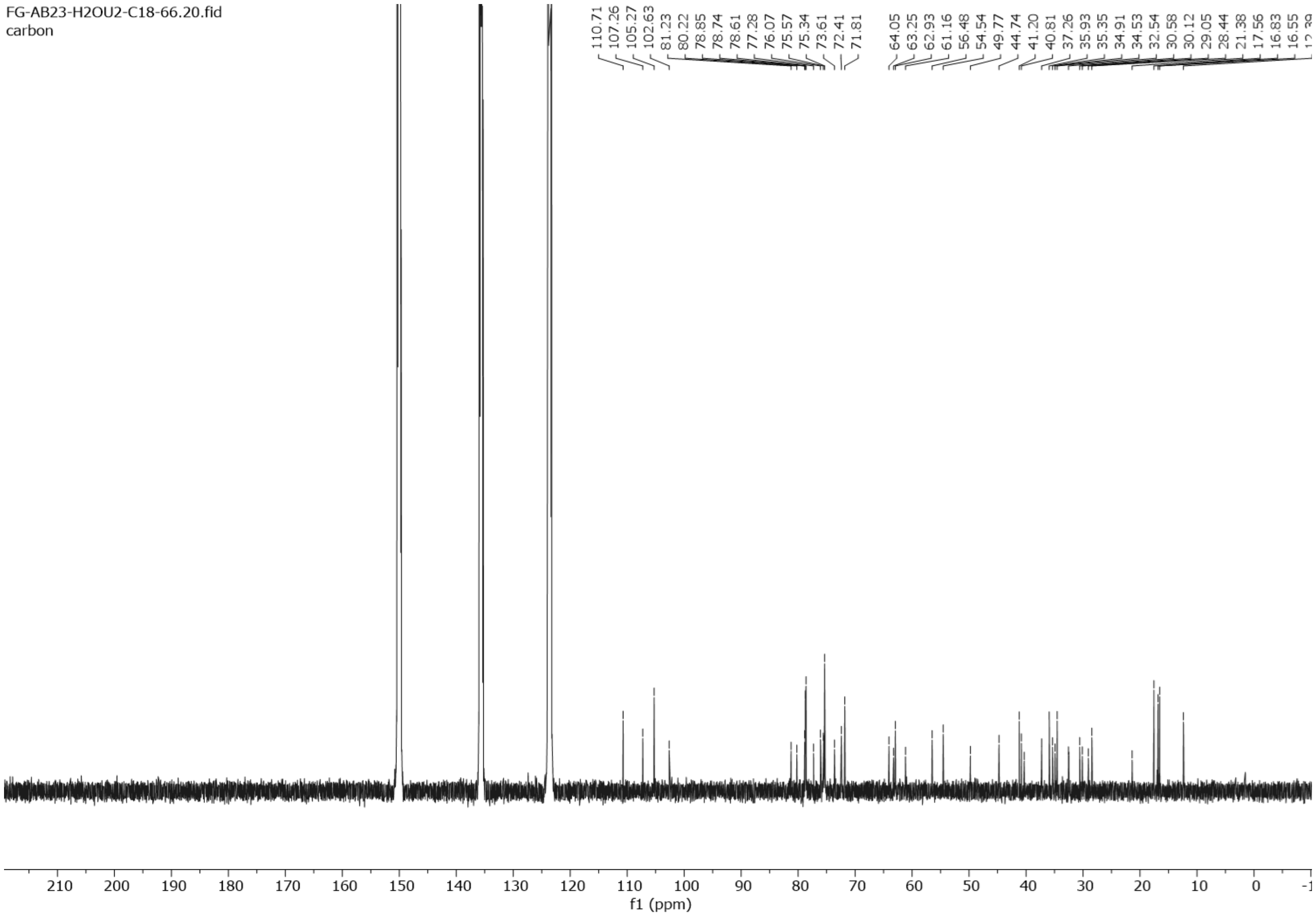

Figure S19. ^{13}**C NMR spectrum of Bractofuranoside B (5) (700 MHz, Pyridine-*d*$_5$)**

Elemental Composition Report **Page 1**

Tolerance = 5.0 mDa / DBE: min = -1.5, max = 50.0
Element prediction: Off
Number of isotope peaks used for i-FIT = 3

Monoisotopic Mass, Even Electron Ions
256 formula(e) evaluated with 2 results within limits (up to 50 closest results for each mass)
Elements Used:
C: 0-60 H: 0-100 O: 0-50
ABRA-8C 124 (2.234)
1: TOF MS ES-

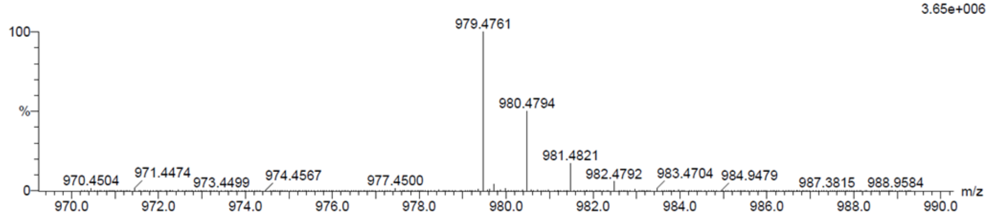

Minimum:	80.00				-1.5				
Maximum:	100.00		5.0	10.0	50.0				
Mass	RA	Calc. Mass	mDa	PPM	DBE	i-FIT	Norm	Conf(%)	Formula
979.4761	100.00	979.4750	1.1	1.1	9.5	603.7	0.000	99.99	C46 H75 O22
		979.4809	-4.8	-4.9	0.5	613.4	9.717	0.01	C39 H79 O27

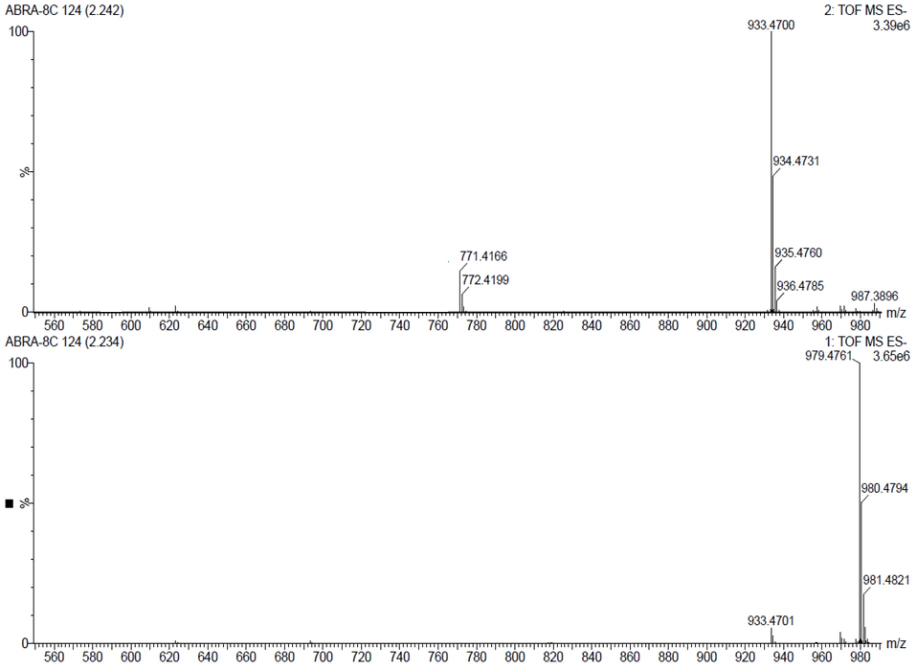

Figure S20. Elemental composition calculated for $C_{46}H_{75}O_{22}$ [M + CH_3COO^-]$^-$ and HRESI MS^E (negative mode) of Bractofuranoside C (6).

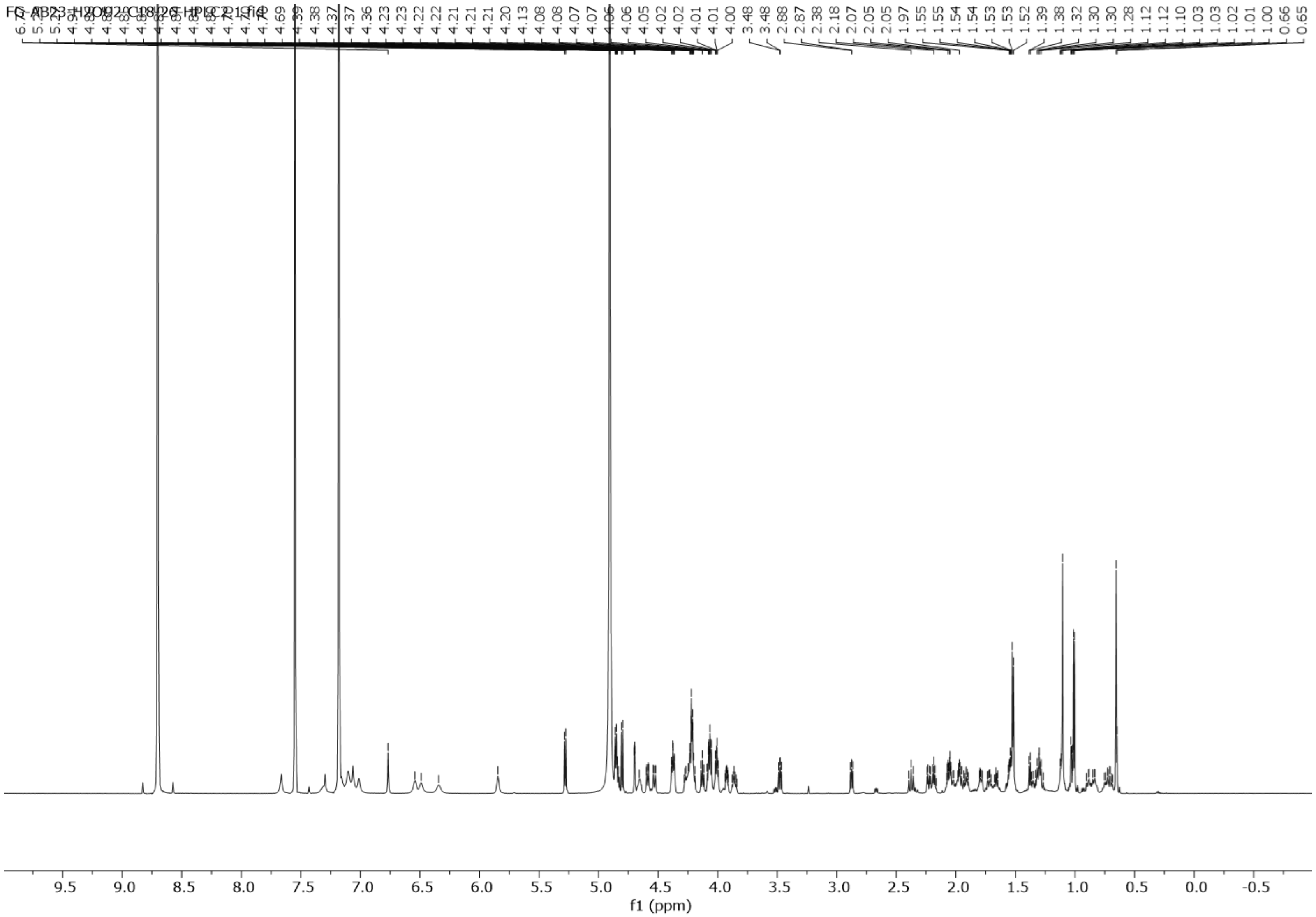

Figure S21. ^{1}H NMR spectrum of Bractofuranoside C (6) (700 MHz, Pyridine-*d_5*)

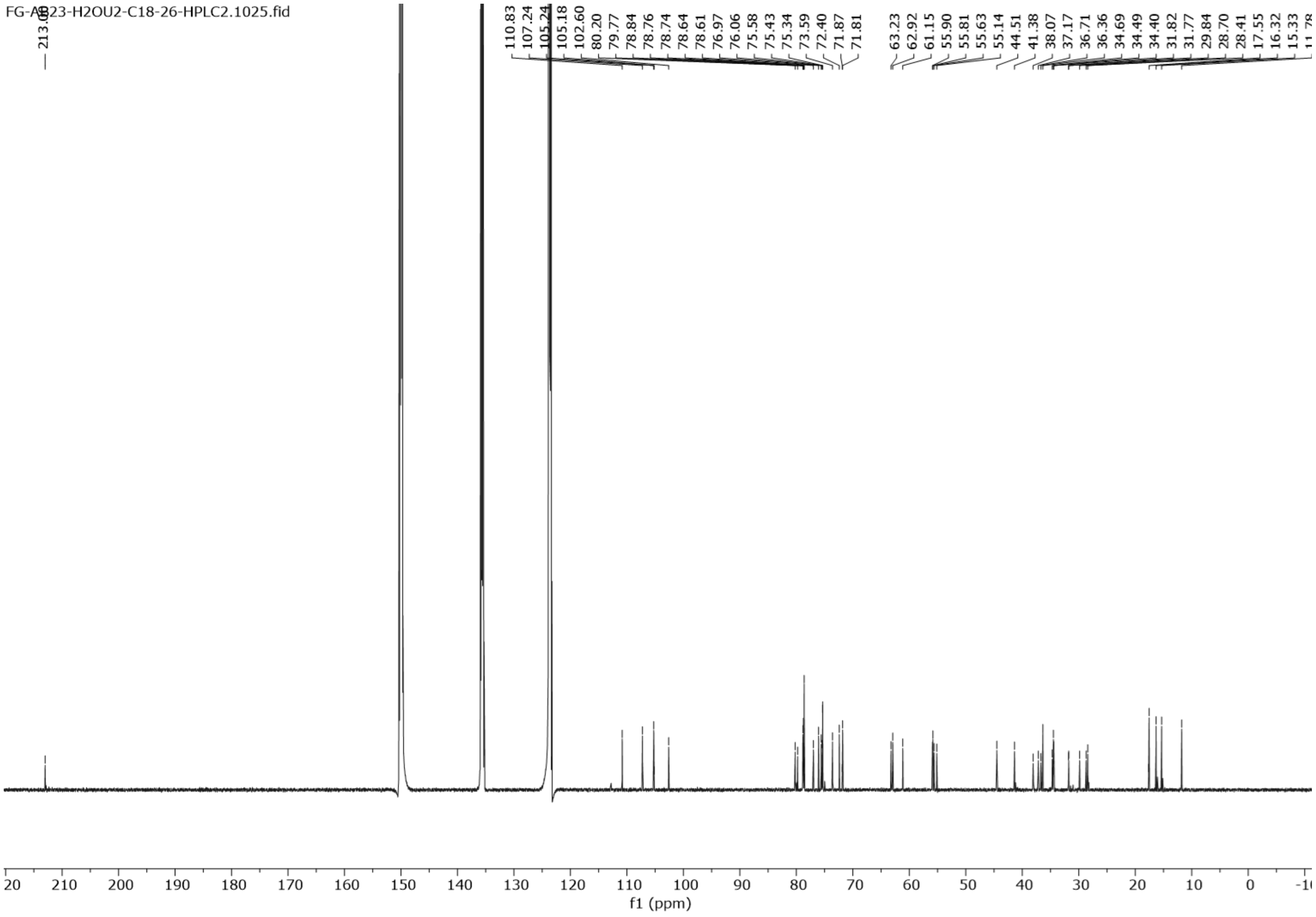

Figure S22. ^{13}**C NMR spectrum of Bractofuranoside C (6) (700 MHz, Pyridine-*d*$_5$)**

Elemental Composition Report **Page 1**

Tolerance = 5.0 mDa / DBE: min = -1.5, max = 50.0
Element prediction: Off
Number of isotope peaks used for i-FIT = 3

Monoisotopic Mass, Even Electron Ions
248 formula(e) evaluated with 2 results within limits (up to 50 closest results for each mass)
Elements Used:
C: 0-60 H: 0-100 O: 0-50

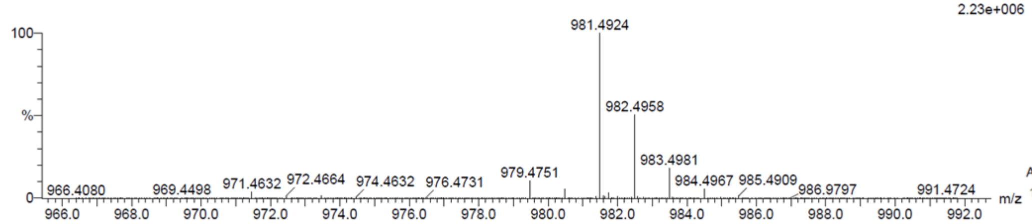

Minimum:	80.00				-1.5				
Maximum:	100.00		5.0	10.0	50.0				
Mass	RA	Calc. Mass	mDa	PPM	DBE	i-FIT	Norm	Conf(%)	Formula
981.4924	100.00	981.4906	1.8	1.8	8.5	628.5	0.000	99.97	C46 H77 O22
		981.4965	-4.1	-4.2	-0.5	636.8	8.249	0.03	C39 H81 O27

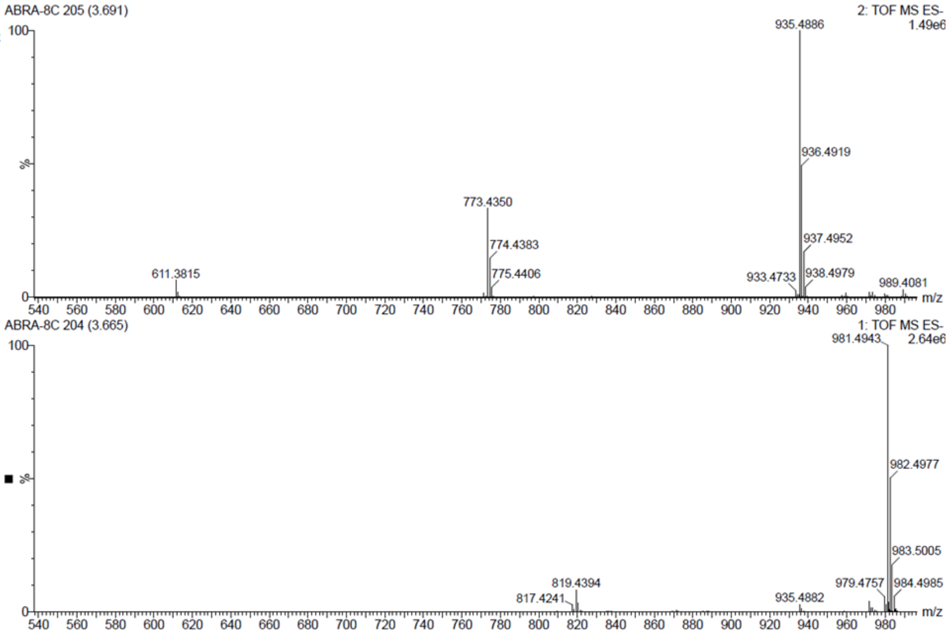

Figure S23. Elemental composition calculated for $C_{46}H_{77}O_{22}$ [M + CH_3COO^-]$^-$ and HRESI MS^E (negative mode) of Bractofuranoside D (7).

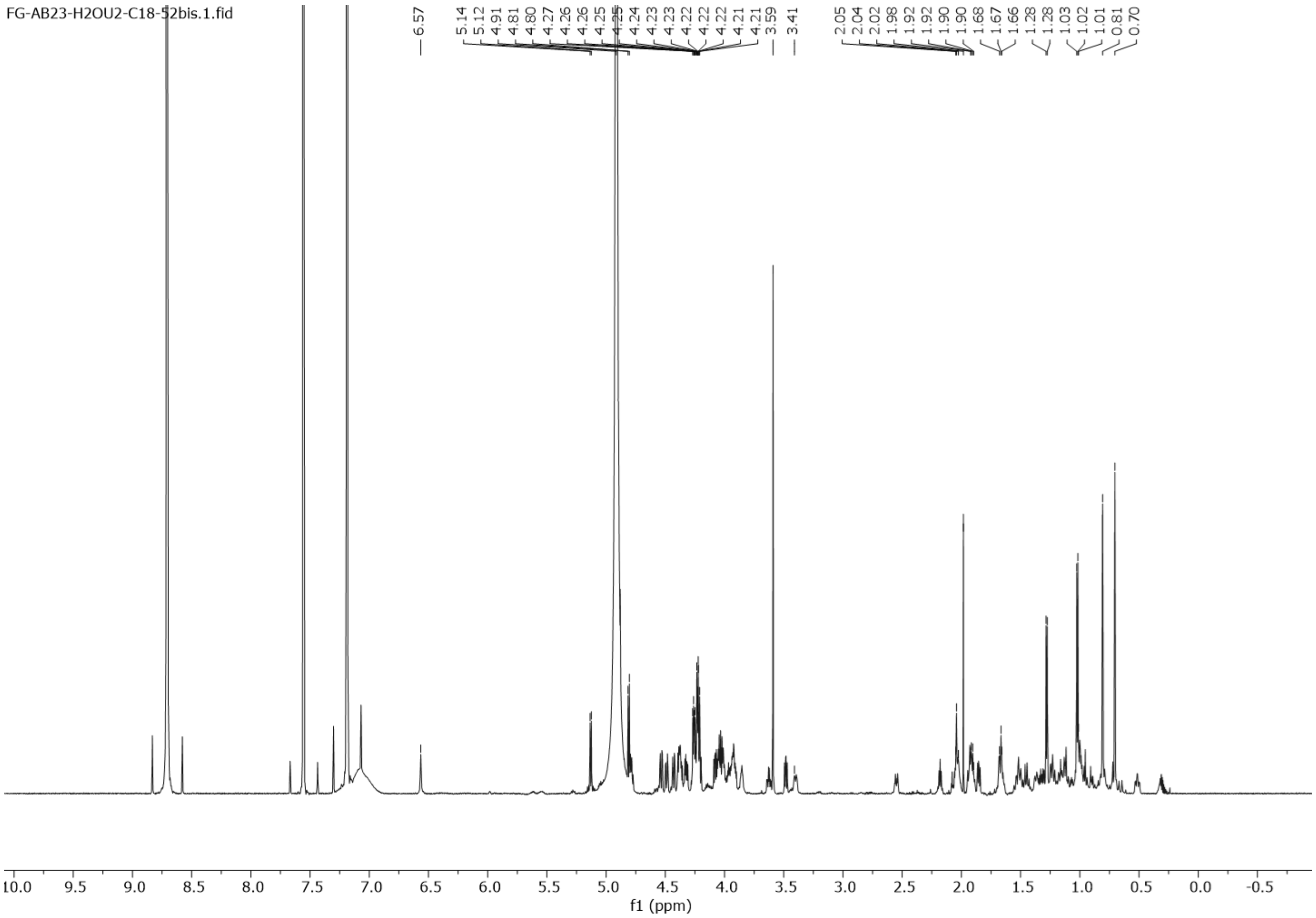

Figure S24. ^{1}**H NMR spectrum of Bractofuranoside D (7) (700 MHz, Pyridine-*****d_5*****)**

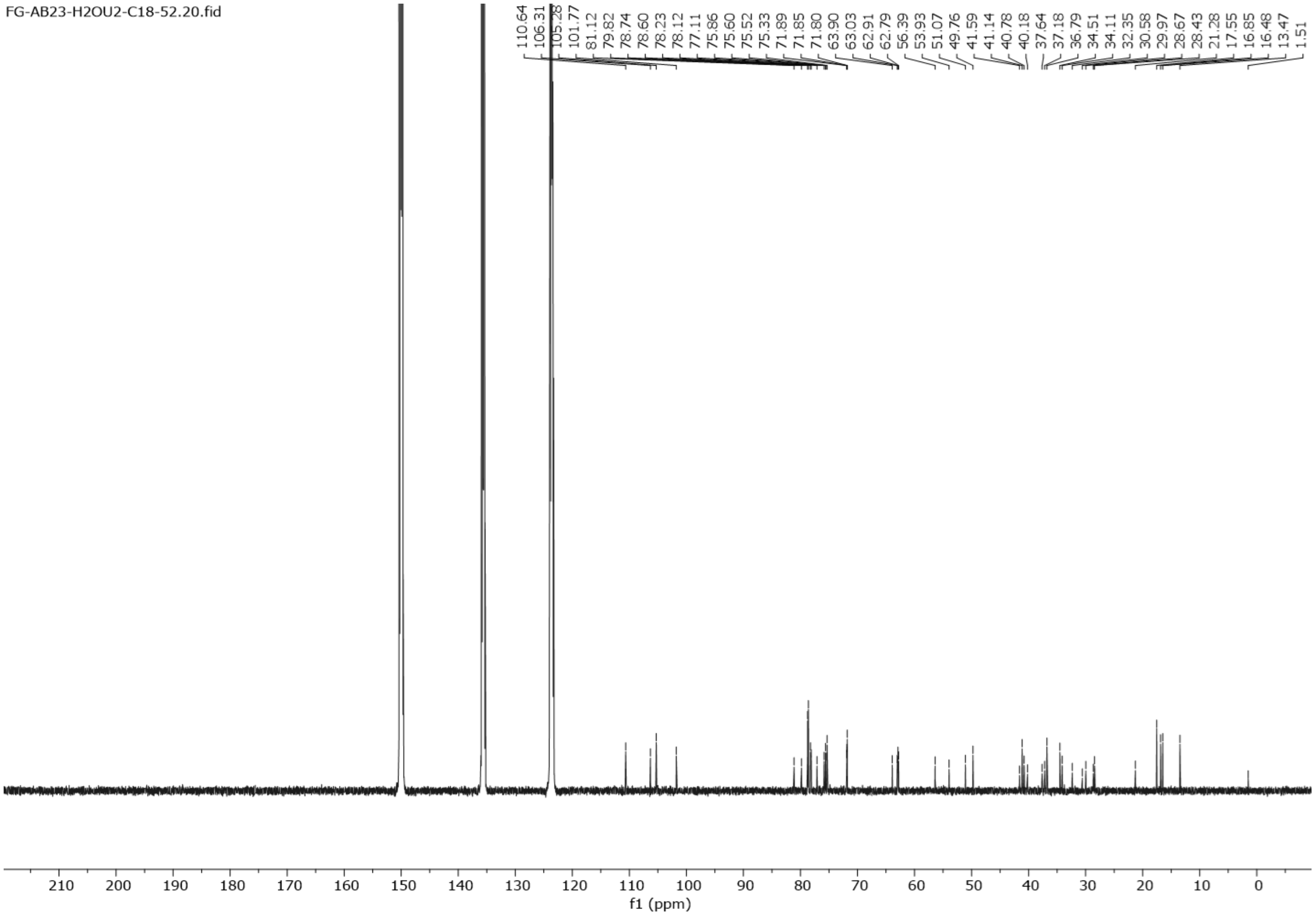

Figure S25. ^{13}**C NMR spectrum of Bractofuranoside D (7) (700 MHz, Pyridine-*d*$_5$)**

Elemental Composition Report **Page 1**

Tolerance = 5.0 mDa / DBE: min = -1.5, max = 50.0
Element prediction: Off
Number of isotope peaks used for i-FIT = 3

Monoisotopic Mass, Even Electron Ions
248 formula(e) evaluated with 2 results within limits (up to 50 closest results for each mass)
Elements Used:
C: 0-60 H: 0-100 O: 0-50

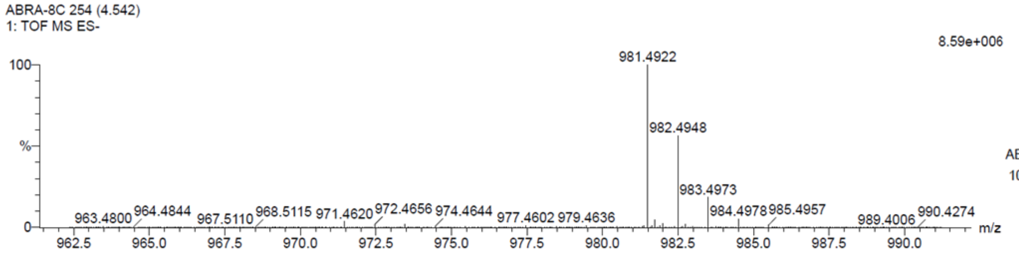

Minimum:	80.00				-1.5				
Maximum:	100.00		5.0	10.0	50.0				
Mass	RA	Calc. Mass	mDa	PPM	DBE	i-FIT	Norm	Conf(%)	Formula
981.4922	100.00	981.4906	1.6	1.6	8.5	619.2	0.001	99.88	C46 H77 O22
		981.4965	-4.3	-4.4	-0.5	625.9	6.764	0.12	C39 H81 O27

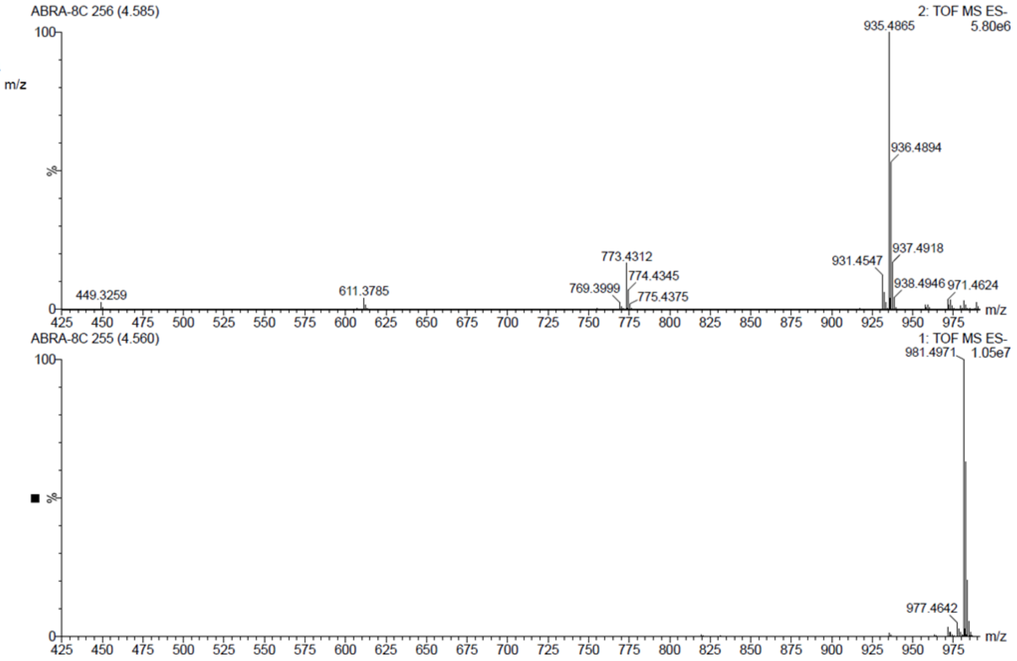

Figure S26. Elemental composition calculated for $C_{46}H_{77}O_{22}$ [M + CH_3COO^-]$^-$ and HRESI MS^E (negative mode) of Bractofuranoside E (8).

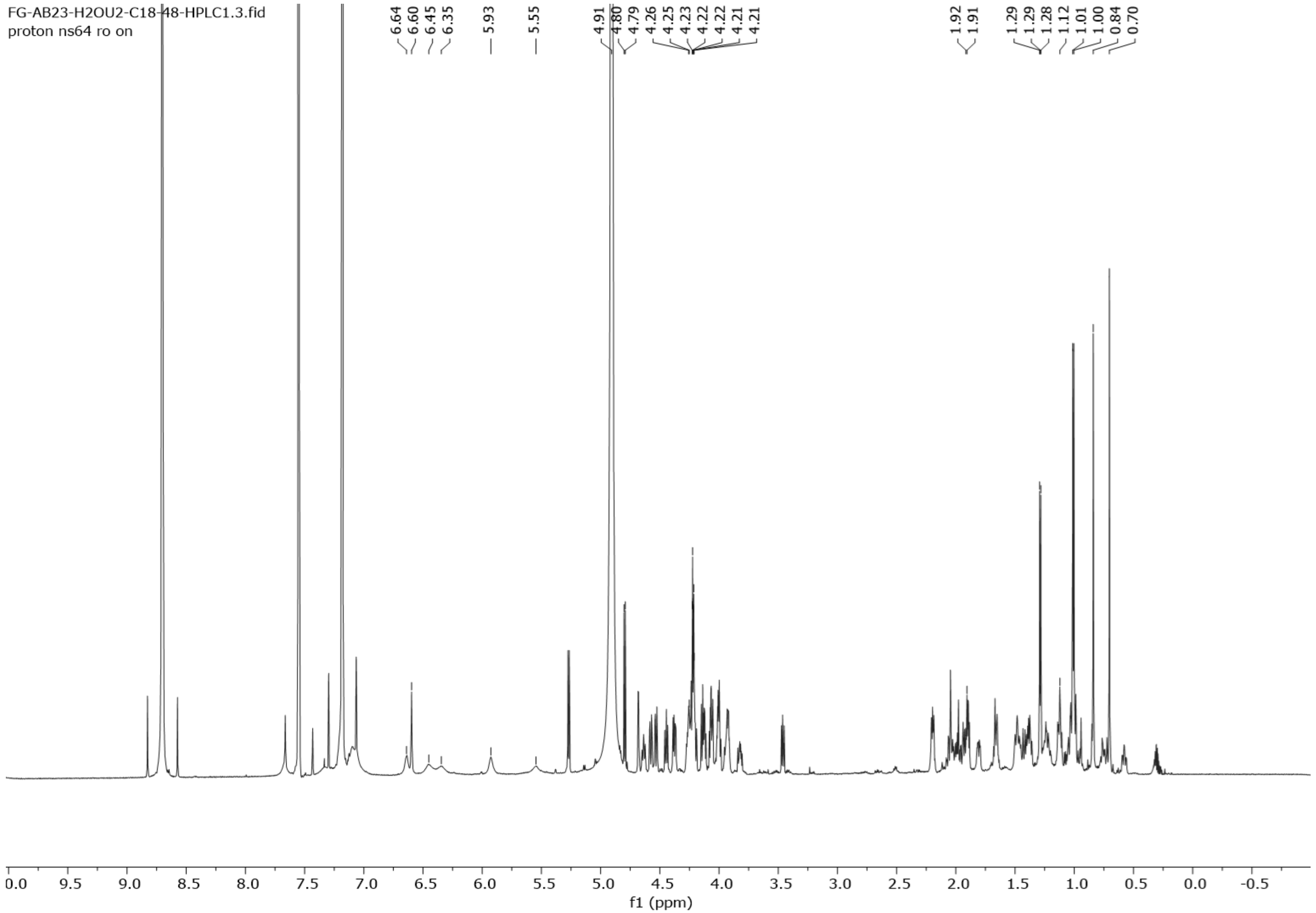

Figure S27. ^{1}H NMR spectrum of Bractofuranoside E (8) (700 MHz, Pyridine-d_5)

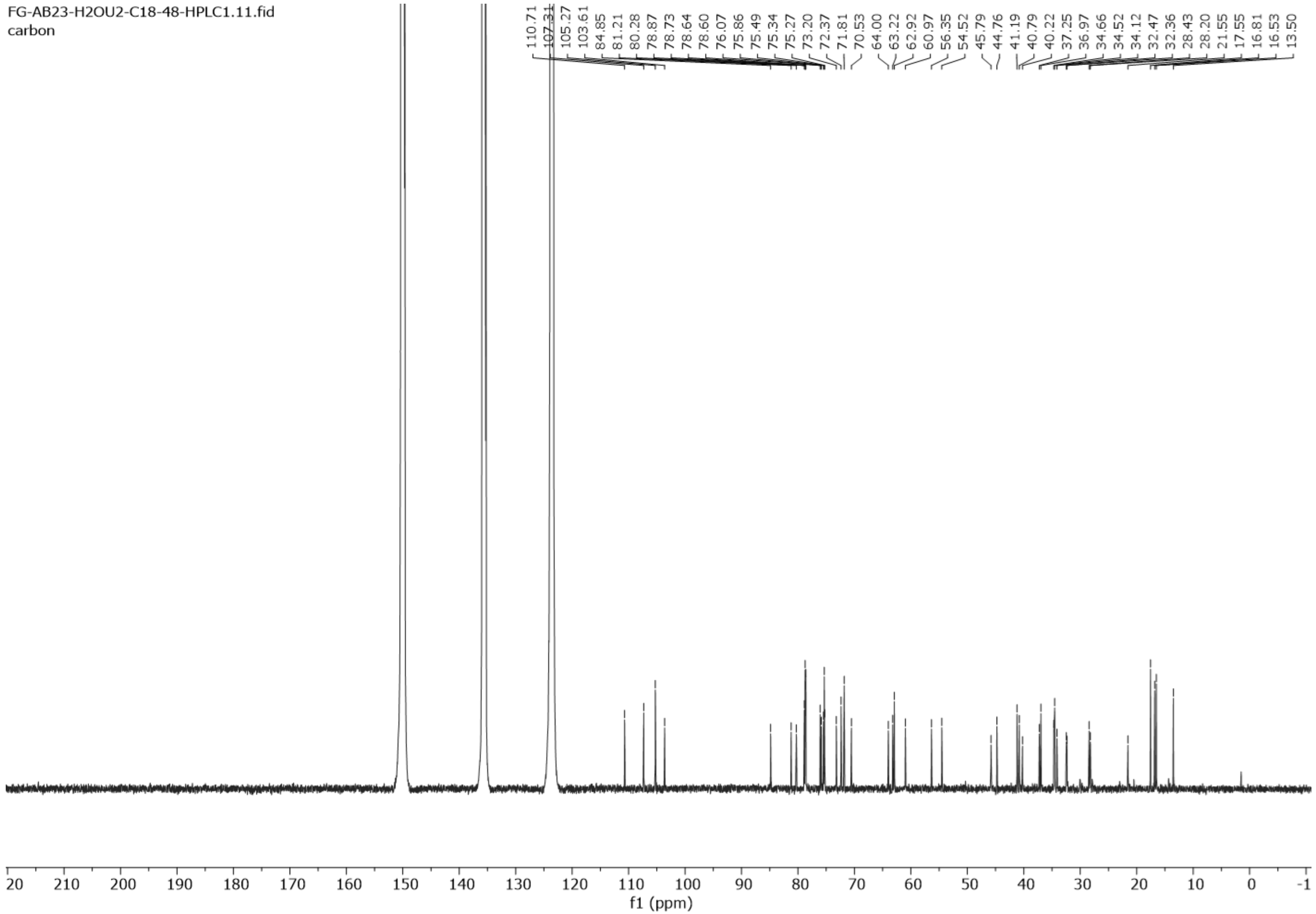

Figure S28. **^{13}C NMR spectrum of Bractofuranoside E (8) (700 MHz, Pyridine-*d*5)**

Elemental Composition Report

Tolerance = 5.0 mDa / DBE: min = -1.5, max = 50.0
Element prediction: Off
Number of isotope peaks used for i-FIT = 3

Monoisotopic Mass, Even Electron Ions
246 formula(e) evaluated with 2 results within limits (up to 50 closest results for each mass)
Elements Used:
C: 0-60 H: 0-100 O: 0-50
ABRA-8C 74 (1.337)
1: TOF MS ES-

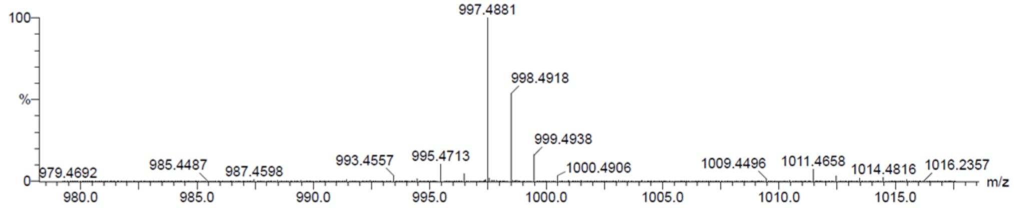

Minimum:	80.00				-1.5				
Maximum:	100.00		5.0	10.0	50.0				
Mass	RA	Calc. Mass	mDa	PPM	DBE	i-FIT	Norm	Conf(%)	Formula
997.4881	100.00	997.4856	2.5	2.5	8.5	537.1	0.022	97.84	C46 H77 O23
		997.4914	-3.3	-3.3	-0.5	540.9	3.837	2.16	C39 H81 O28

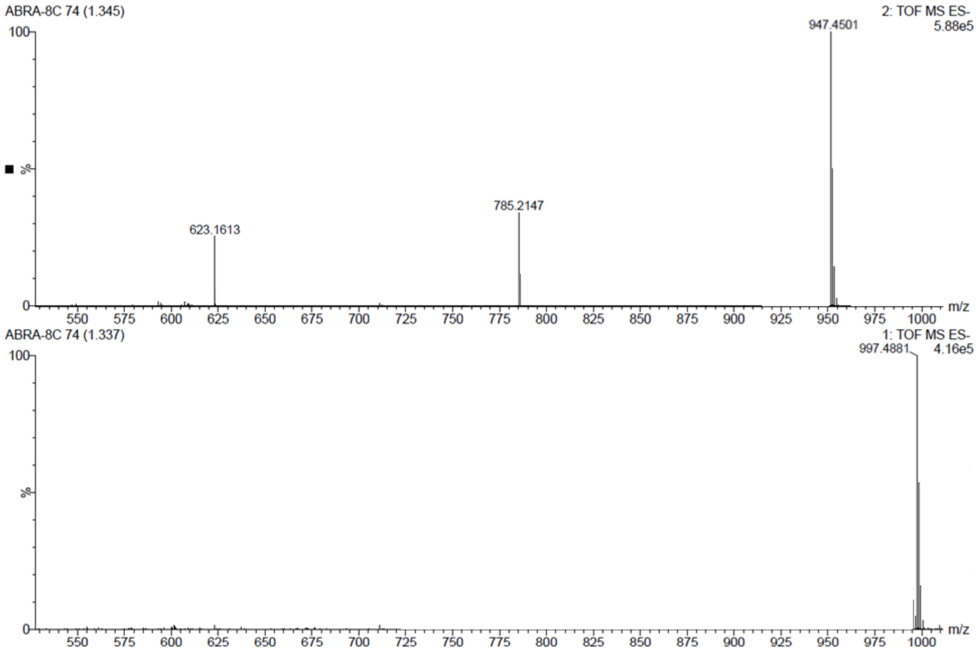

Figure S29. Elemental composition calculated for $C_{46}H_{77}O_{23}$ [M + CH_3COO^-]$^-$ and HRESI MS^E (negative mode) of Bractofuranoside F (9).

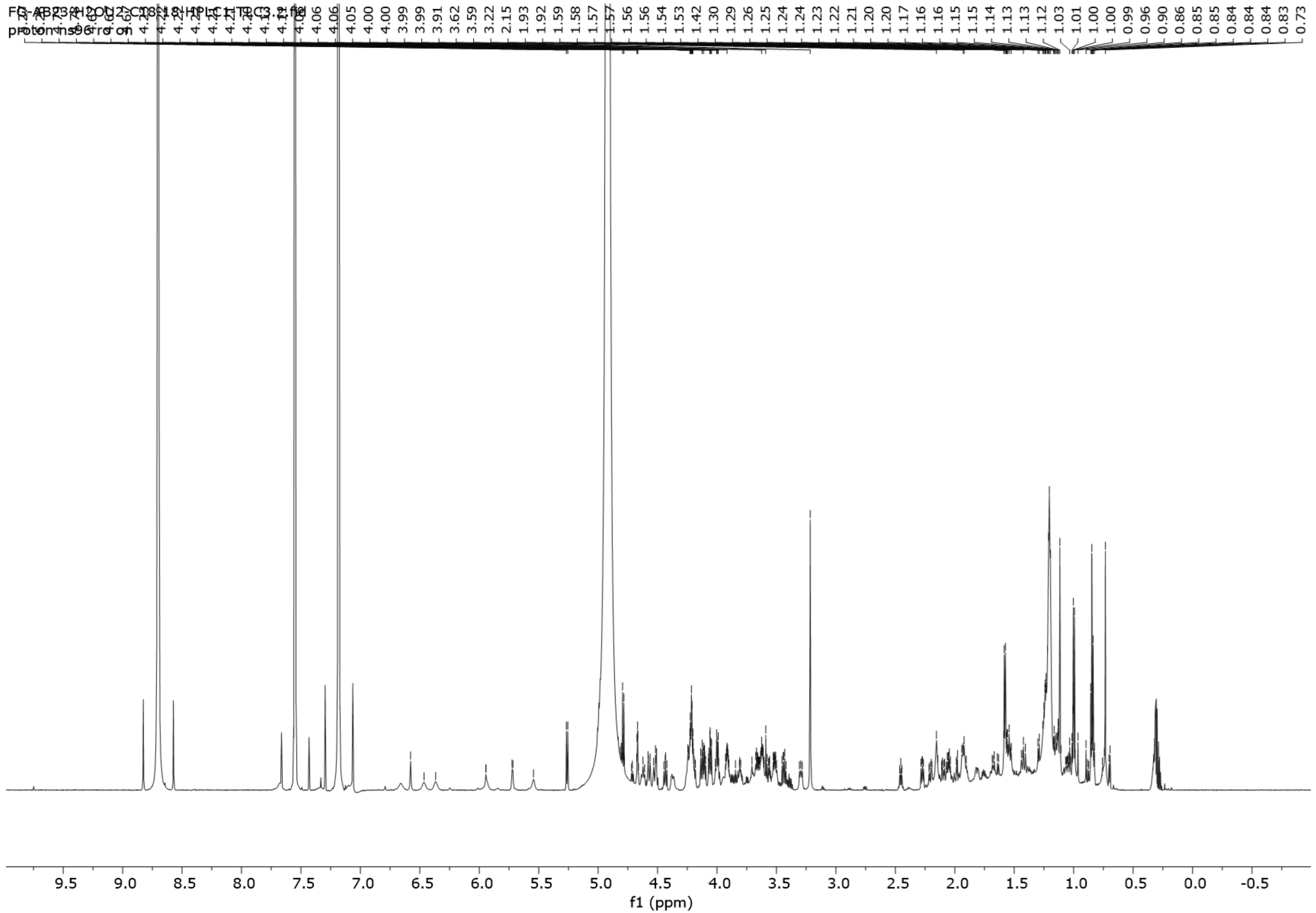

Figure S30. **^{1}H NMR spectrum of Bractofuranoside F (9) (700 MHz, Pyridine-*d₅*)**

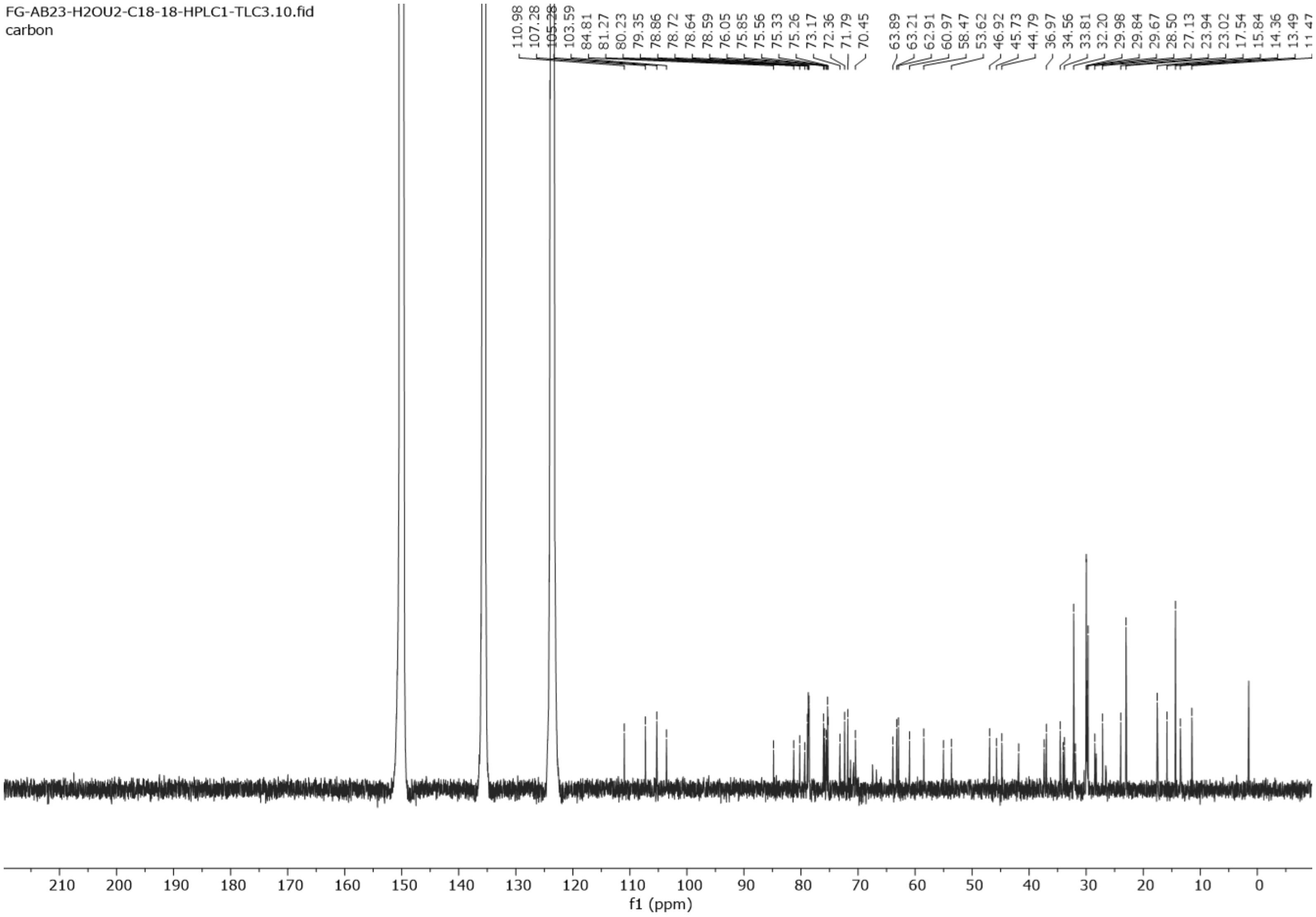

Figure S31. ^{13}C NMR spectrum of Bractofuranoside F (9) (700 MHz, Pyridine-*d_5*)

Elemental Composition Report **Page 1**

Tolerance = 5.0 mDa / DBE: min = -1.5, max = 50.0
Element prediction: Off
Number of isotope peaks used for i-FIT = 3

Monoisotopic Mass, Even Electron Ions
270 formula(e) evaluated with 2 results within limits (up to 50 closest results for each mass)
Elements Used:
C: 0-60 H: 0-100 O: 0-50
ABRA-8C 163 (2.922)
1: TOF MS ES-

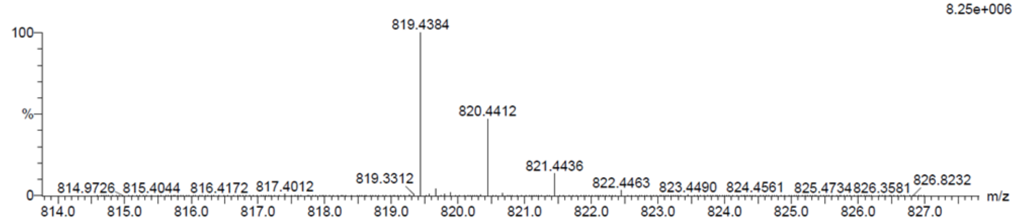

Mass	RA	Calc. Mass	mDa	PPM	DBE	i-FIT	Norm	Conf(%)	Formula
Minimum:	80.00				-1.5				
Maximum:	100.00		5.0	10.0	50.0				
819.4384	100.00	819.4378	0.6	0.7	7.5	751.8	0.000	100.00	C40 H67 O17
		819.4413	-2.9	-3.5	29.5	762.4	10.650	0.00	C58 H59 O4

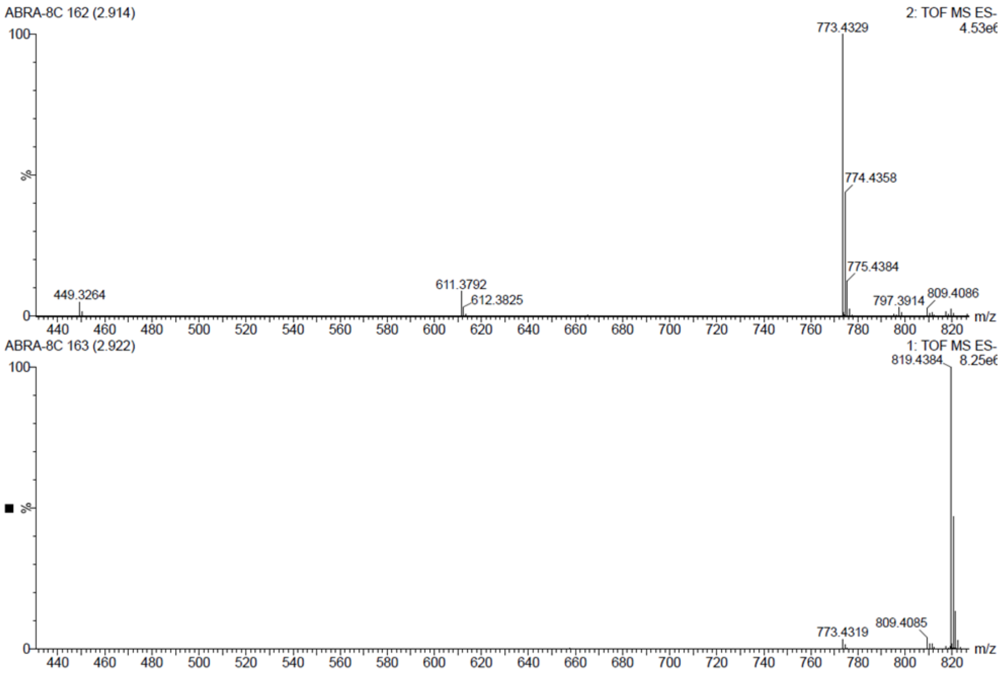

Figure S32. Elemental composition calculated for $C_{40}H_{67}O_{17}$ [M + CH_3COO^-]$^-$ and HRESI MS^E (negative mode) of Bractofuranoside G (10).

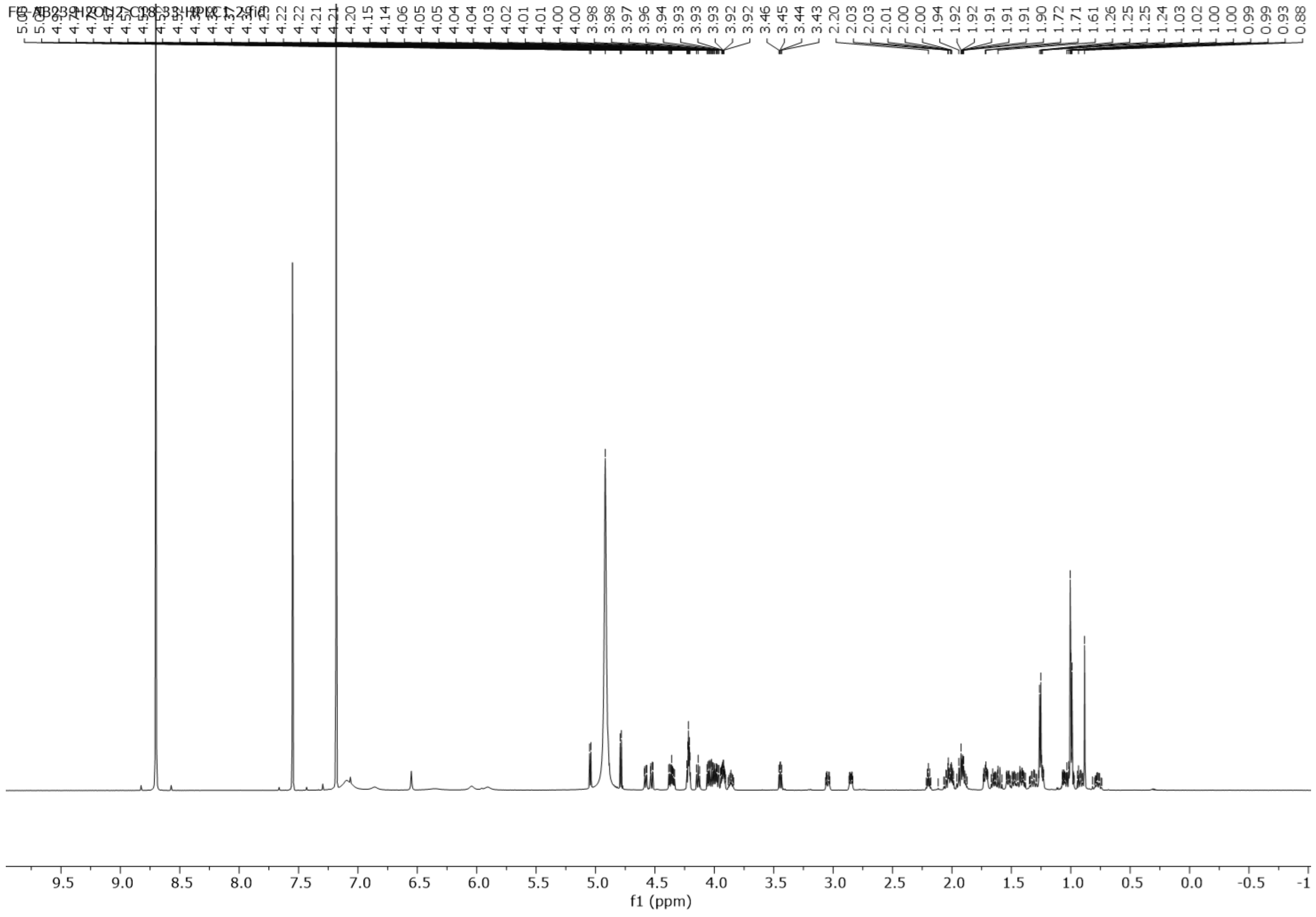

Figure S33. ^{1}H NMR spectrum of Bractofuranoside G (10) (700 MHz, Pyridine-d_5)

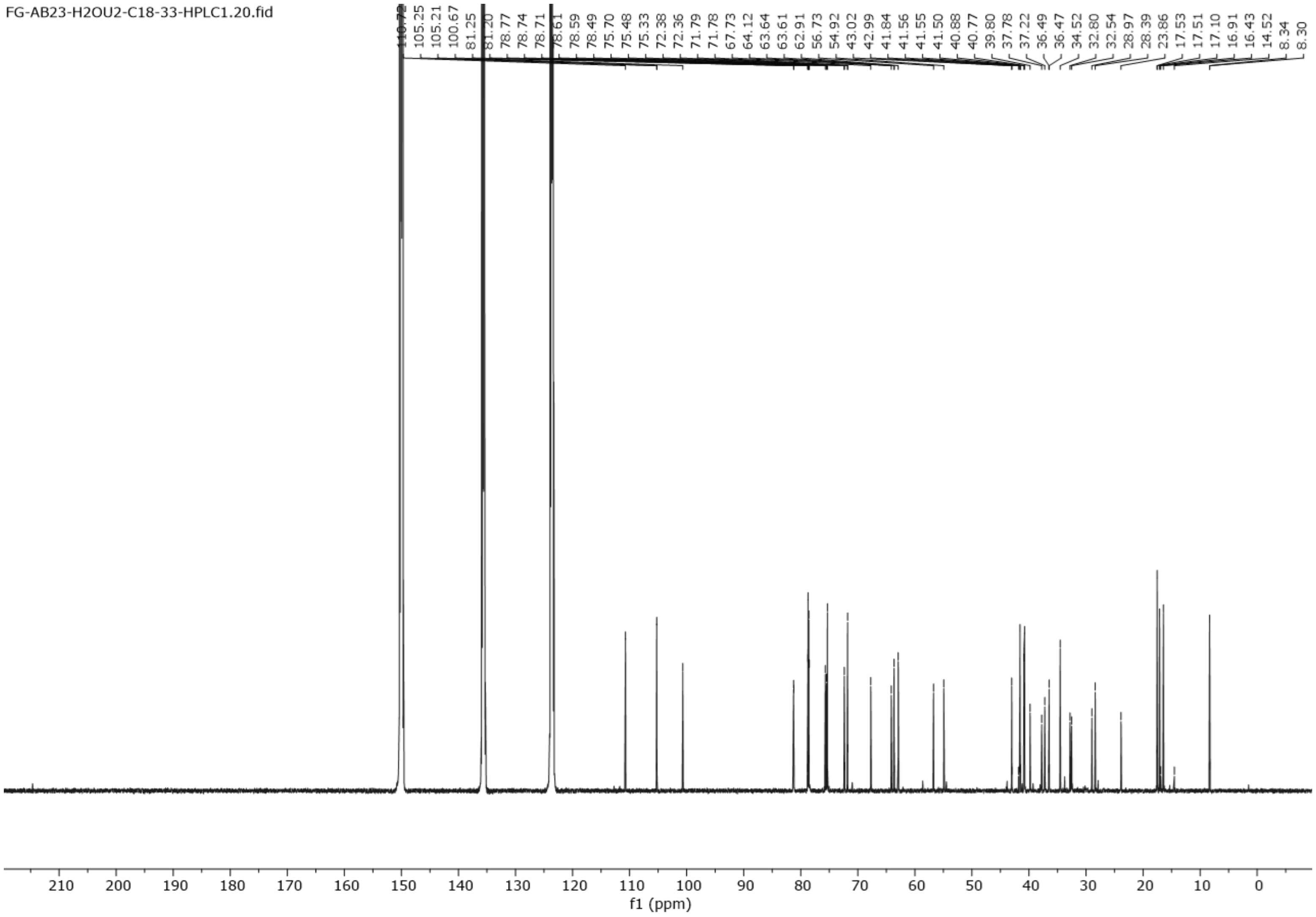

Figure S34. ^{13}C **NMR spectrum of Bractofuranoside G (10) (700 MHz, Pyridine-*d_5*)**

Elemental Composition Report **Page 1**

Tolerance = 5.0 mDa / DBE: min = -1.5, max = 50.0
Element prediction: Off
Number of isotope peaks used for i-FIT = 2

Monoisotopic Mass, Even Electron Ions
260 formula(e) evaluated with 2 results within limits (up to 50 closest results for each mass)
Elements Used:
C: 0-60 H: 0-100 O: 0-50
ABRA-11C 172 (3.097)
1: TOF MS ES-

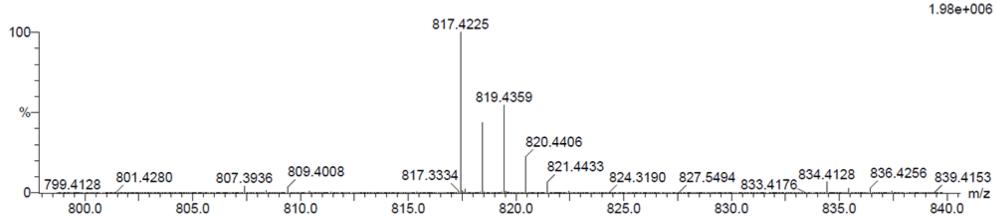

Minimum:	80.00				-1.5				
Maximum:	100.00		5.0	10.0	50.0				
Mass	RA	Calc. Mass	mDa	PPM	DBE	i-FIT	Norm	Conf(%)	Formula
817.4225	100.00	817.4222	0.3	0.4	8.5	459.0	0.000	100.00	C40 H65 O17
		817.4257	-3.2	-3.9	30.5	469.2	10.197	0.00	C58 H57 O4

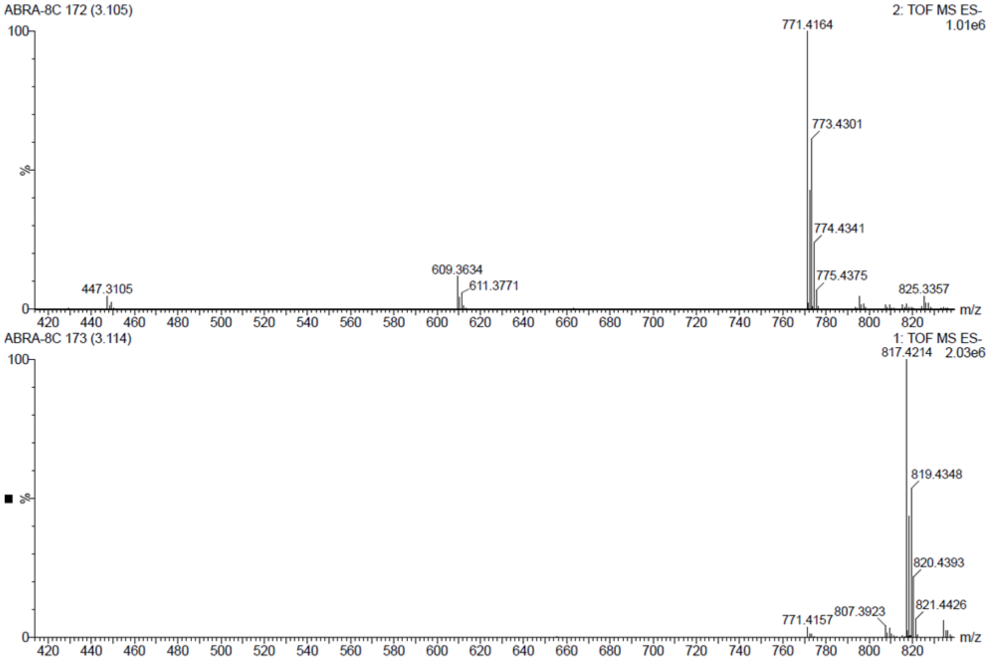

Figure S35. Elemental composition calculated for $C_{40}H_{65}O_{17}$ [M + CH_3COO^-]$^-$ and HRESI MS^E (negative mode) of Bractofuranoside H (11).

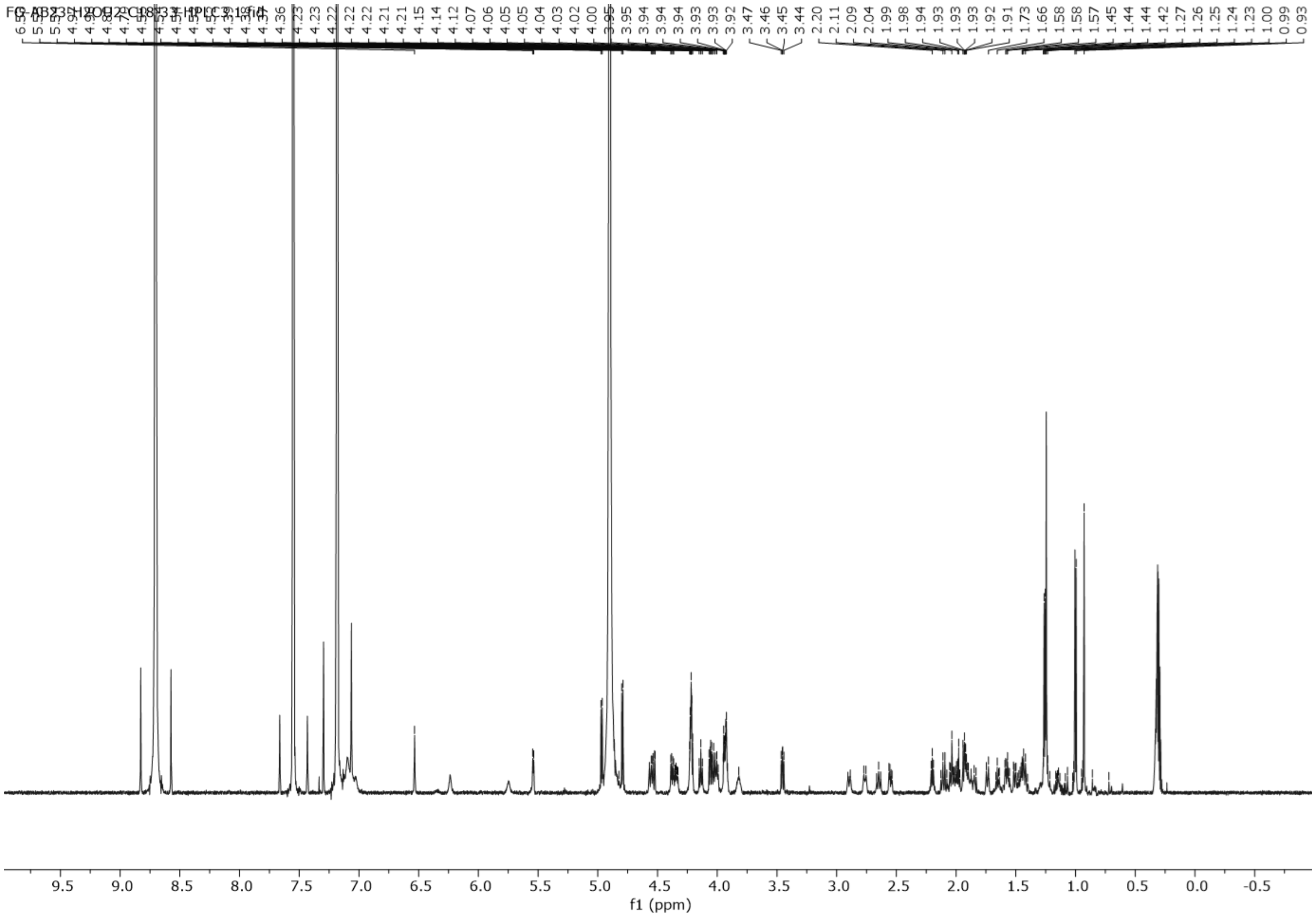

Figure S36. ^{1}H NMR spectrum of Bractofuranoside H (11) (700 MHz, Pyridine-*d*$_5$)

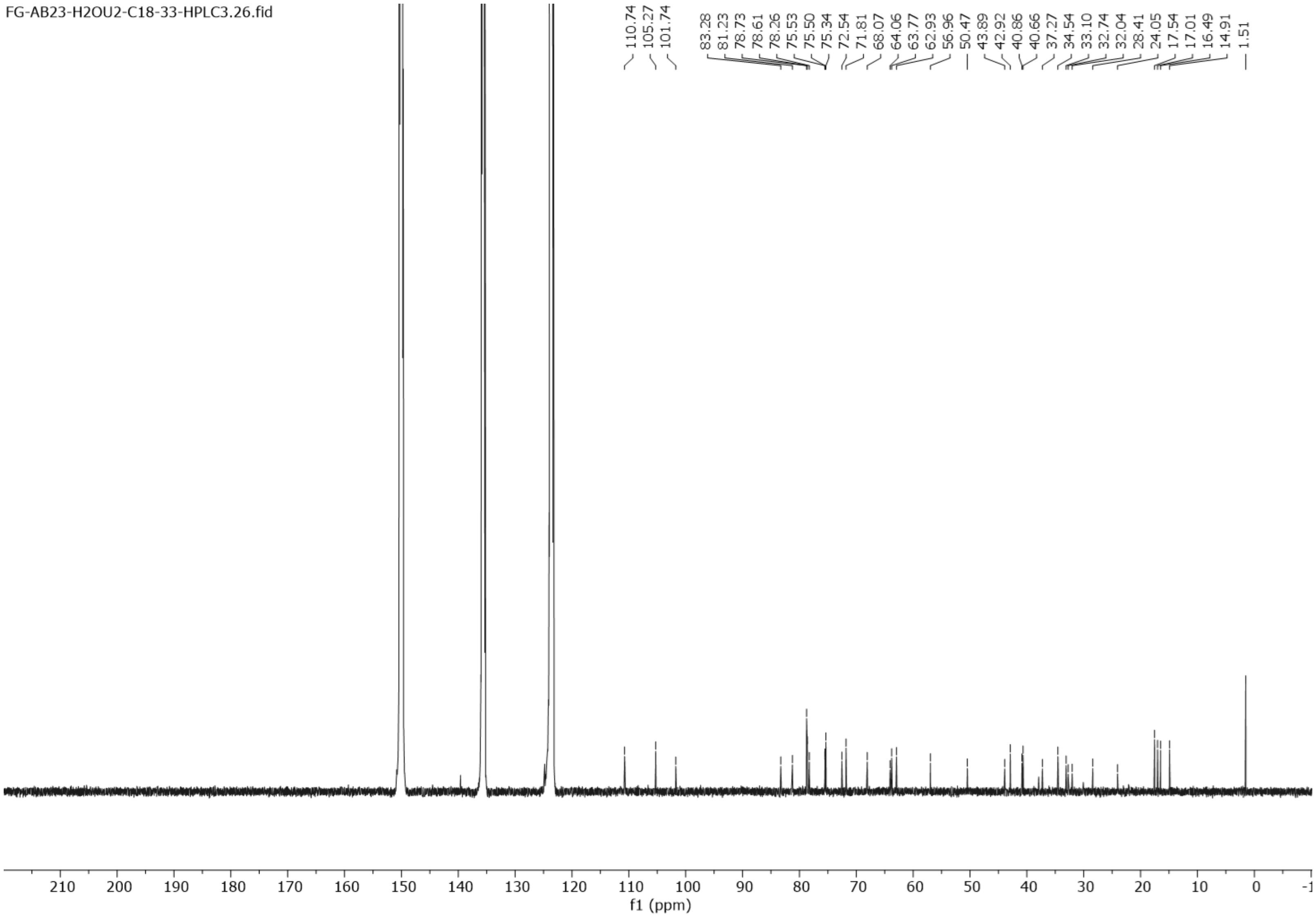

Figure S37. **^{13}C NMR spectrum of Bractofuranoside H (11) (700 MHz, Pyridine-d_5)**

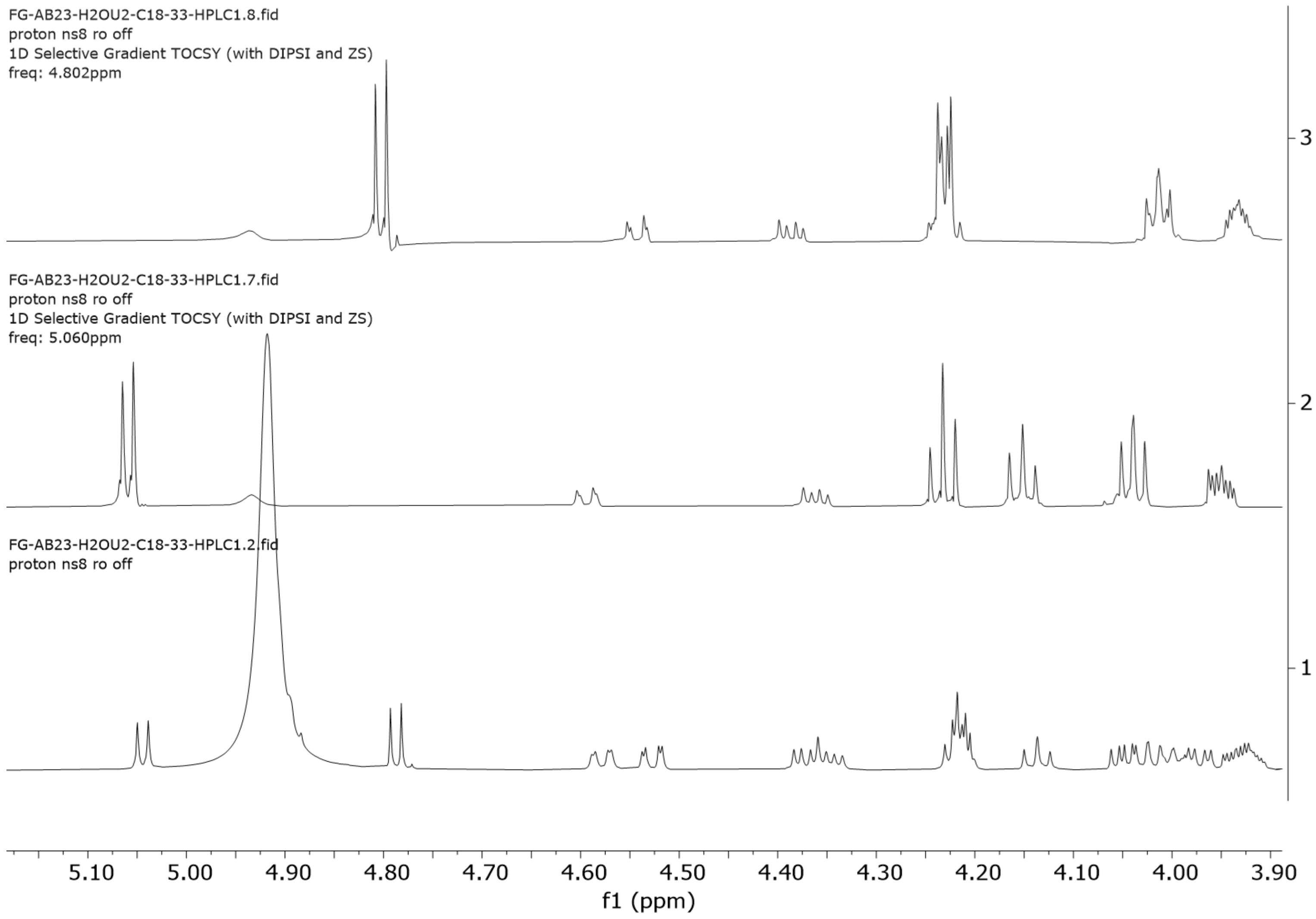

Figure S38. Example of 1D TOCSY spectra (120 ms): Sugar chain of Bractofuranoside G (10). (700 MHz, Pyridine-d_5)

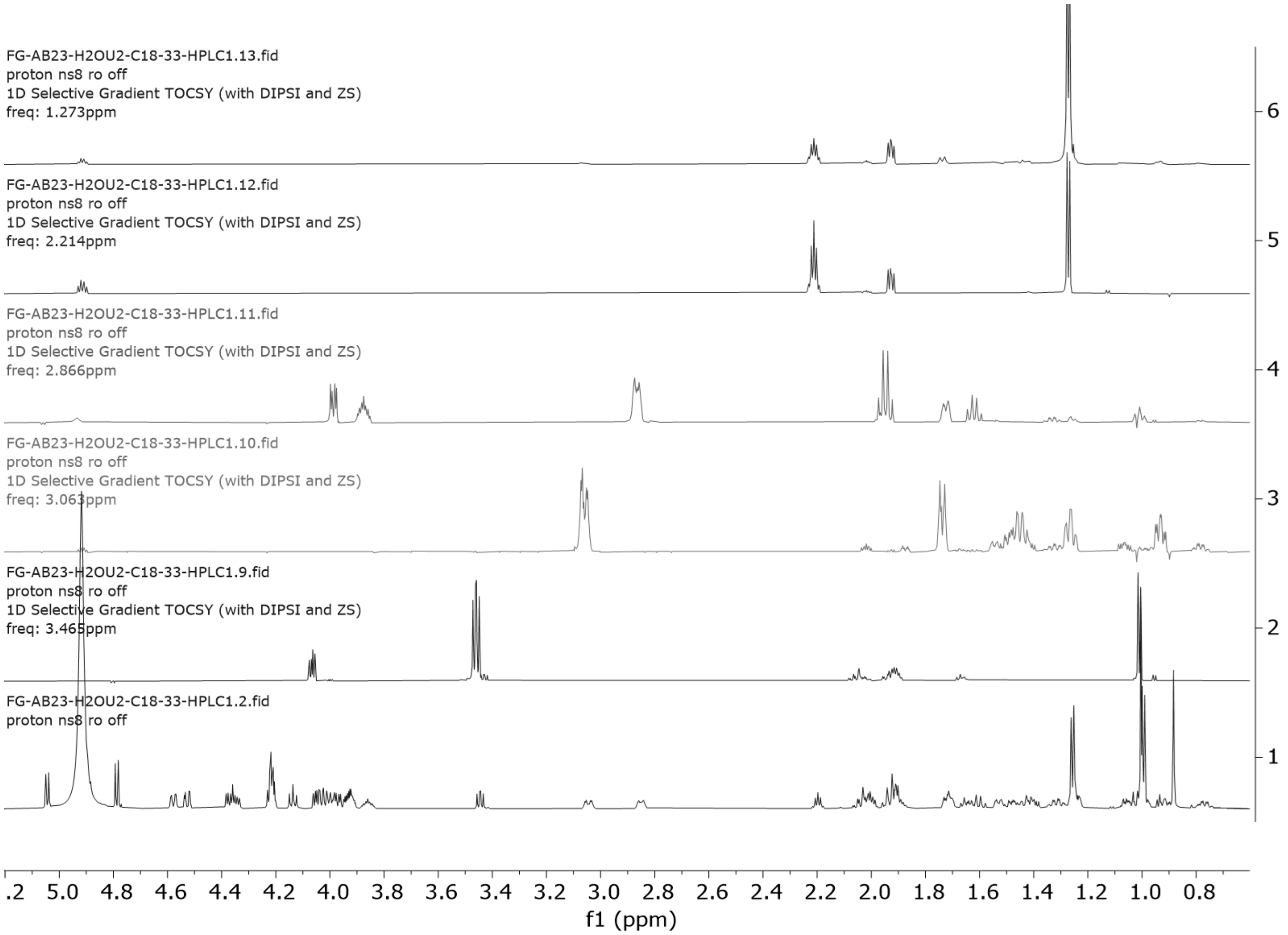

Figure S39. Example of 1D TOCSY spectra (120 ms): Aglycone of Bractofuranoside G (10). (700 MHz, Pyridine-d_5)

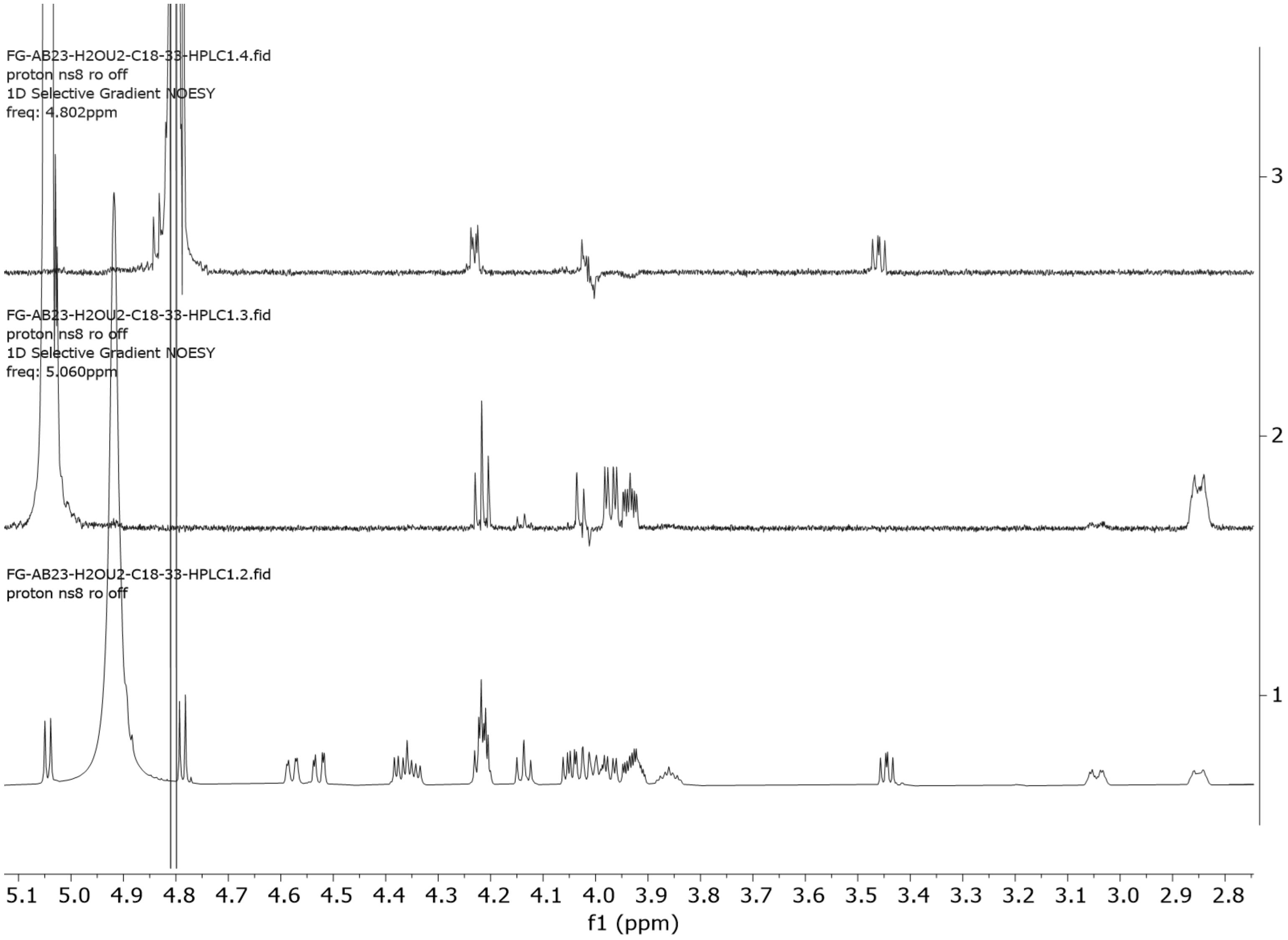

Figure S40. Example of 1D NOESY spectra (300 ms): Sugar chain of Bractofuranoside G (10). (700 MHz, Pyridine-d_5)

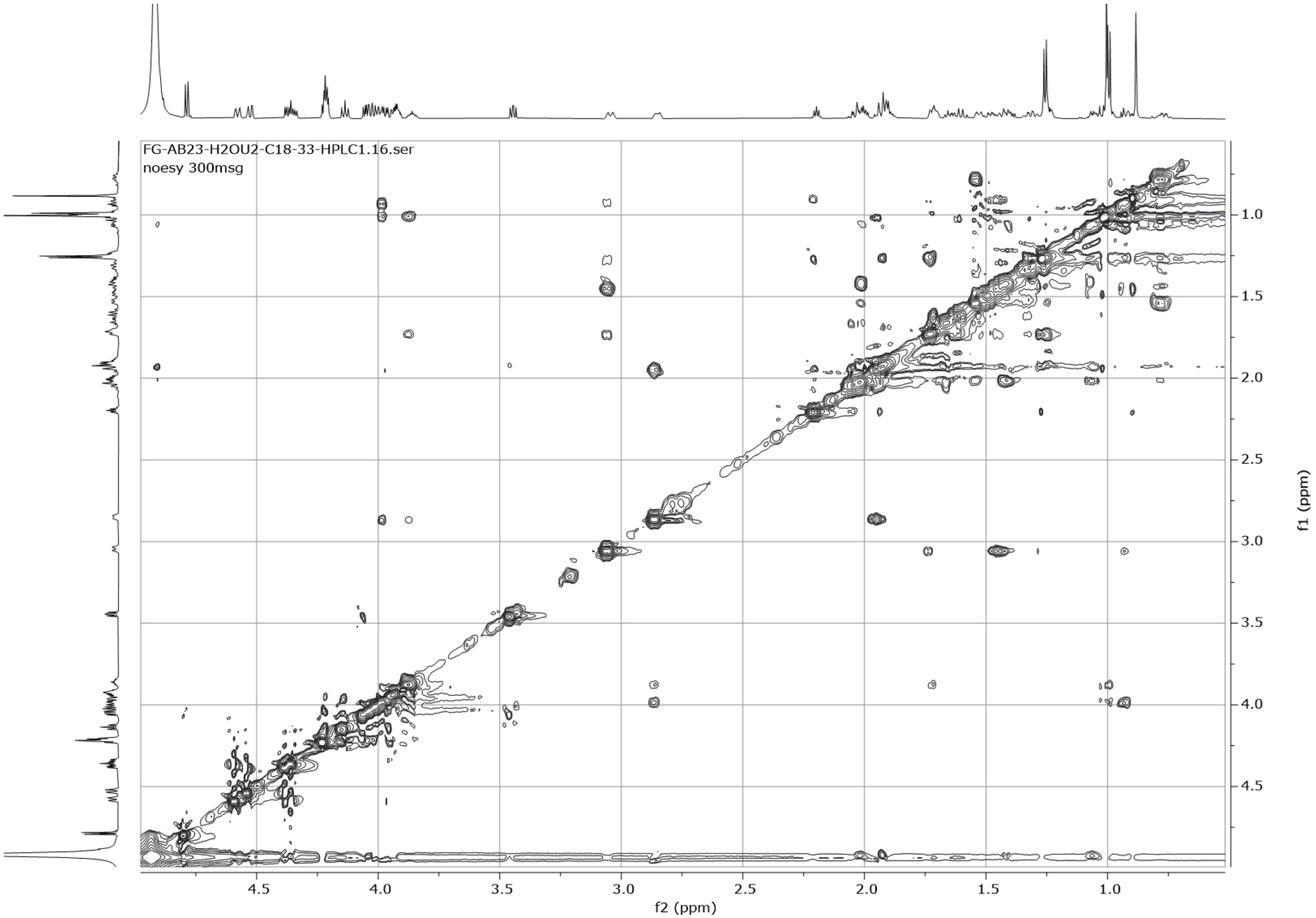

Figure S41. Example of 2D NOESY spectra (300 ms): Aglycone of Bractofuranoside G (10). (700 MHz, Pyridine-d_5)
